# MYO1C is a urinary extracellular vesicle biomarker and mediator of podocyte injury in diabetic nephropathy

**DOI:** 10.1172/jci.insight.194604

**Published:** 2026-01-22

**Authors:** Zihao Zhao, Qianqian Yan, Sijie Zhou, Fengxun Liu, Yong Liu, Jingjing Ren, Shaokang Pan, Zhenjie Liu, Dongwei Liu, Zhangsuo Liu, Jiayu Duan

**Affiliations:** 1 Department of Radiology, Department of Nephrology, and; 2 Nephrology Research Center, The First Affiliated Hospital of Zhengzhou University, Zhengzhou, China.; 3 Key Laboratory of Precision Diagnosis and Treatment for Chronic Kidney Disease in Henan Province, Zhengzhou, China.; 4 Department of Nephrology, Institute of Kidney Diseases, West China Hospital of Sichuan University, Chengdu, China.; 5 Department of Medicine, Brigham and Women’s Hospital and Harvard Medical School, Boston, Massachusetts, USA.; 6 Innovation Center of Basic Research for Metabolic-Associated Fatty Liver Disease, Ministry of Education of China, Zhengzhou, China.; 7 Tianjian Laboratory of Advanced Biomedical Sciences, Academy of Medical Sciences Zhengzhou University, Zhengzhou, China.; 8 The Jockey Club School of Public Health and Primary Care, The Chinese University of Hong Kong, Hong Kong, China.

**Keywords:** Inflammation, Nephrology, Apoptosis, Biomarkers, Urology

## Abstract

Type 2 diabetic nephropathy (T_2_DN) is a major complication of type 2 diabetes and a leading cause of chronic kidney disease. This study aimed to explore Myosin IC (MYO1C) as both a candidate biomarker and elucidate its role as a mechanistic mediator of podocyte injury in T_2_DN. Using urinary extracellular vesicle RNA biomarkers identified from a training and validation cohort of 33 type 2 diabetes and 40 patients with T_2_DN, we developed a machine learning diagnostic model for T_2_DN. The model achieved an AUC of 0.877 in validation and performed well in an independent test cohort with an AUC of 0.824. MYO1C was identified as the most influential feature in the final model. Mechanistic investigations in vitro and in vivo revealed that high glucose and high-fat conditions induced podocyte injury, inflammation, and apoptosis, with increased MYO1C expression. MYO1C knockdown in vitro and in vivo reduced podocyte damage and inflammatory responses. MYO1C overexpression enhanced p38, p-CREB, and TNF-α levels, while p38 inhibition mitigated these effects. These findings support MYO1C not only as a potential urinary biomarker for T_2_DN but also as a key pathogenic driver that promotes podocyte injury via p38 MAPK signaling.

## Abstract 2

<strong>MYO1C as a Urinary Extracellular Vesicles Biomarker and Promotes Podocyte Injury via p38 MAPK Activation in Diabetic Nephropathy</strong>

## Graphical abstract

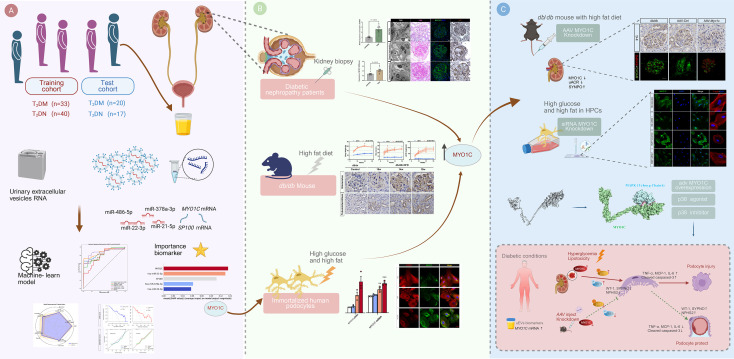

## Introduction

Diabetic nephropathy (DN), a severe microvascular complication of diabetes, has become a major cause of chronic kidney disease globally, imposing a substantial health burden (1). Over the past 3 decades, the prevalence of DN has risen in parallel with the increasing incidence of diabetes, which currently affects an estimated 529 million individuals worldwide, with type 2 diabetes accounting for 96% of cases (2). Despite advancements in diabetes management, type 2 DN (T_2_DN), a major complication of type 2 diabetes, remains challenging to prevent and treat, which is often leads to irreversible kidney function decline, culminating in end-stage kidney disease (3). T_2_DN is characterized by progressive glomerular damage, proteinuria, and renal dysfunction, driven by a complex interplay of hyperglycemia, oxidative stress, and inflammation, ultimately resulting in podocyte injury and basement membrane thickening (4).

Current diagnostic approaches for T_2_DN rely on clinical parameters such as urinary albumin, serum creatinine, and diabetic retinopathy (5). However, kidney biopsy remains the gold standard for definitive diagnosis, although it is invasive and limited in detecting early-stage disease or accurately predicting progression (6). Consequently, there is an urgent need for innovative biomarkers and mechanistic insights to enable effectual diagnosis and the development of targeted therapeutic strategies (7). Urinary extracellular vesicles (uEVs) are nanoscale vesicles secreted by cells of the urinary system and are increasingly recognized as a promising source of noninvasive biomarkers for diseases (8). EVs are classified into several subtypes, including 2 major subsets: exosomes and ectosomes or microvesicles, based on their size, biogenesis, and cargo content (9). Exosomes typically range in size from 40 to 150 nm and are generated through the endocytic pathway, where inward budding of multivesicular bodies forms exosomes. These exosomes are subsequently released into the extracellular space upon fusion of the multivesicular bodies with the plasma membrane (9). Microvesicles, on the other hand, are shed directly from the plasma membrane through outward budding. Both types of EVs contain a wide range of molecular cargo, including proteins, lipids, and RNA, reflecting the molecular characteristics of their parent cells (10). The noninvasive nature of urine collection, combined with the unique protective effect conferred by the EV phospholipid bilayer membrane on its cargo, makes uEVs a highly suitable source for monitoring kidney health (11). Their composition can alter in response to renal pathological changes, potentially mediate crosstalk between glomerular and tubular compartments, and participate in disease progression as injured cells along the nephron release EVs (12). These properties position uEVs as valuable tools not only for disease diagnosis but also for understanding disease mechanisms in chronic kidney conditions such as DN. Evidence suggests that uEVs carry markers of pathogenic processes such as inflammation and fibrosis, which is supported by omics-based analyses identifying specific uEV RNAs as promising diagnostic indicators (13, 14). The enrichment of certain RNA species in uEVs, compared with their source cells, further suggests the presence of selective RNA packaging mechanisms, which may enhance the biomarker specificity of uEVs-derived RNAs. Our previous research contributed to this field by employing microarray and sequencing analysis to construct a comprehensive transcriptomic profile of differentially expressed RNAs in uEVs from patients with biopsy-T_2_DN and type 2 diabetes, providing a data foundation for further investigation of uEVs biomarkers in T_2_DN (15).

Myosin IC (MYO1C) is a member of myosin class I in the myosin superfamily and was the first mammalian single-headed myosin to be purified, cloned and sequenced (16). MYO1C plays a crucial role in various cellular functions — including intracellular transport, adhesion, motility, membrane tension regulation, and signal transduction pathways — and has been implicated in several human diseases (17, 18). In the kidney, MYO1C has been shown to be particularly important for maintaining podocyte structure and function. It interacts with membrane proteins like nephrin and Neph1, facilitating their movement to the podocyte membrane (19). In a unilateral ureteral obstruction mouse model, podocyte-specific KO of MYO1C alleviates glomerular fibrosis, suggesting its involvement in fibrosis via TGF-β signaling regulation (20). In DN, metabolic stressors such as hyperglycemia and dyslipidemia activate key mechanisms including inflammatory responses and the p38 MAPK pathway, leading to renal cell injury, podocyte apoptosis, and the formation of a proinflammatory renal microenvironment (21). These pathological changes are recognized as hallmark features in both clinical and experimental settings (22, 23). However, the pathological role of MYO1C in DN remains largely unexplored, and it is unclear whether MYO1C modulates inflammatory signaling pathways, such as p38 MAPK, to mediate podocyte-specific injury.

In this study, candidate differentially expressed RNAs in uEVs were validated in a training cohort comprising 40 patients with T_2_DN and 33 patients with type 2 diabetes and were used to develop a machine learning diagnostic model. The model was subsequently evaluated in an independent test cohort consisting of 17 patients with T_2_DN and 20 patients with type 2 diabetes. We further focused on MYO1C, which emerged as the most important variable in the model feature selection. By integrating functional studies both in vitro and in vivo, we explored the potential of MYO1C as both a biomarker and a therapeutic target for T_2_DN, bridging the gap between diagnostic utility and its functional relevance in disease pathogenesis. Our results suggest that MYO1C is not merely a passive indicator of disease but a true pathogenic driver, particularly through its interaction with inflammatory pathways, including involvement in the p38 MAPK/p-CREB signaling pathway. These findings not only underscore the clinical relevance of MYO1C but also highlight its promise as a therapeutic target in DN.

## Results

### Validation of differential RNA expression in uEVs from patients with T_2_DN and patients with type 2 diabetes.

In this study, 6 highly dysregulated RNAs identified in our previous work (MYO1C, SP100, hsa-miR-21-5p, hsa-miR-22-3p, hsa-miR-378a-3p, and hsa-miR-486-5p) were selected for initial evaluation. A training and validation cohort was used to evaluate the expression of these 6 RNAs by qPCR and to develop a machine learning classification model through feature selection, parameter optimization, and cross-validation. Following the completion of model training, a time-independent test cohort was employed to independently assess the performance and generalizability of the model.

**Figure 1:**
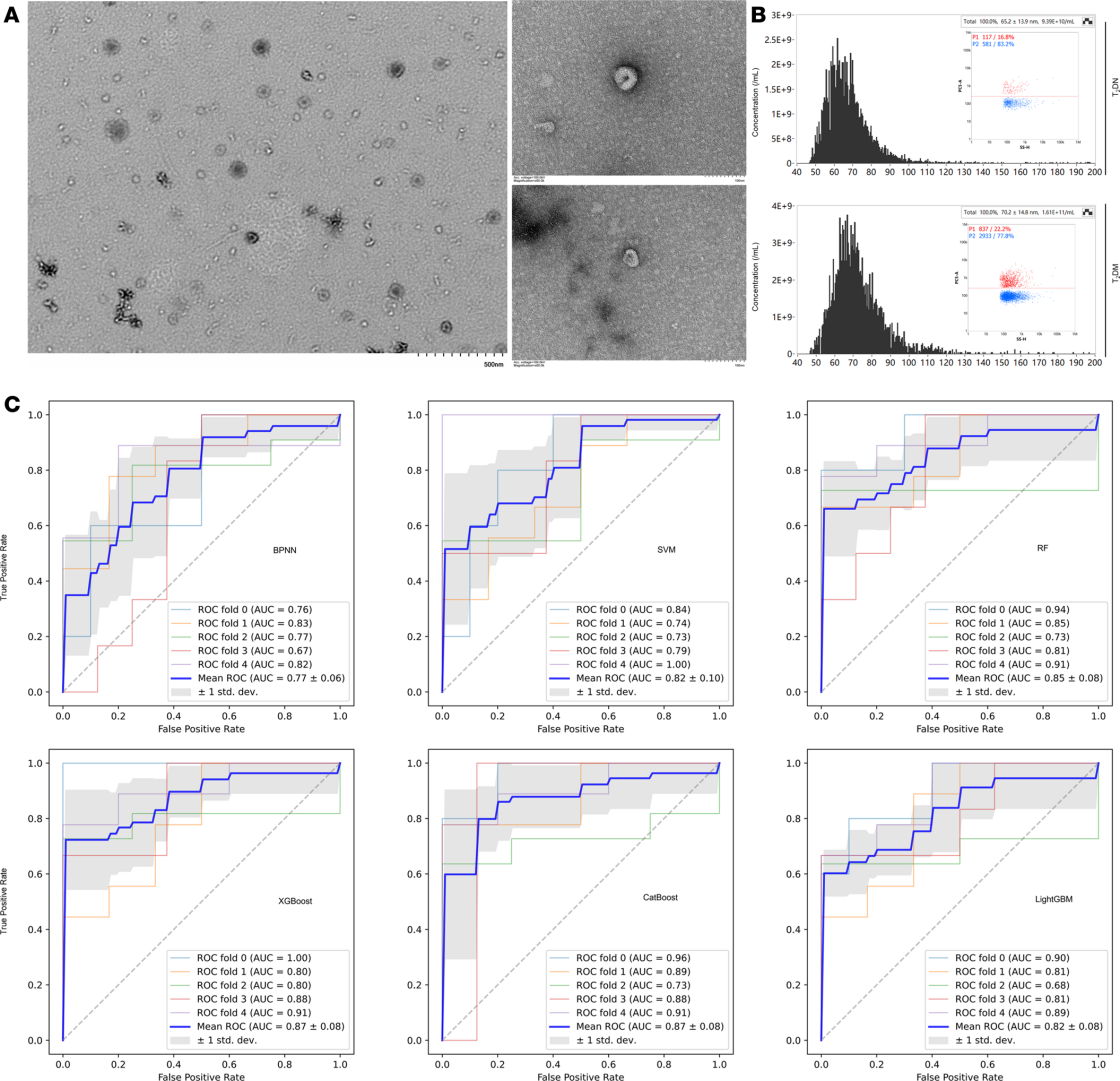
Characterization and validation of uEVs and candidate RNAs in the training cohort. (**A**) Representative TEM images of uEVs, with enlarged views on the right illustrating uEVs from patients with T_2_DN and patients with type 2 diabetes. Scale bar: 100 nm. (**B**) NanoFCM analysis of uEVs particle size distribution histograms and CD 9 fluorescence scatter plots for T_2_DN (top) and T_2_DM (type 2 diabetes, bottom) groups. (**C**) Five-fold cross-validation ROC curves for classification models constructed using BPNN, SVM, RF, XGBoost, CatBoost, and LightGBM, respectively.

The 33 type 2 diabetes and 40 T_2_DN patient groups in the training and validation cohort had no significant differences in age, sex, BMI, or 24-hour urine volume (Supplemental Table 1; supplemental material available online with this article; https://doi.org/10.1172/jci.insight.194604DS1). However, the T_2_DN group exhibited significantly higher 24-hour proteinuria and UACR levels, consistent with clinical features of DN. TEM and NanoFCM analyses confirmed the presence of cup-shaped vesicles (50–120 nm) in both type 2 diabetes and T_2_DN uEVs, with characteristic EV protein marker CD9 expression (Figure 1, A and B). qPCR results revealed significant upregulation of 5 RNAs in T_2_DN uEVs compared with type 2 diabetes, except for hsa-miR-486-5p (*P* = 0.400). Specifically, *MYO1C* mRNA (*P* = 0.004), *SP100* mRNA (*P* = 0.025), hsa-miR-21-5p (*P* < 0.001), hsa-miR-22-3p (*P* = 0.037), and hsa-miR-378a-3p (*P* = 0.018) were significantly elevated in patients with T_2_DN (Supplemental Figure 1A). ROC curve analysis revealed limited discriminatory power of individual RNAs for T_2_DN (AUC, 0.56–0.74; Supplemental Figure 1B), while their correlation with clinical renal function indicators, shown in a heatmap, offered further pathophysiological insights (Supplemental Figure 1C).

### Construction and cross-validation of the machine learning model in the training cohort.

**Figure 2:**
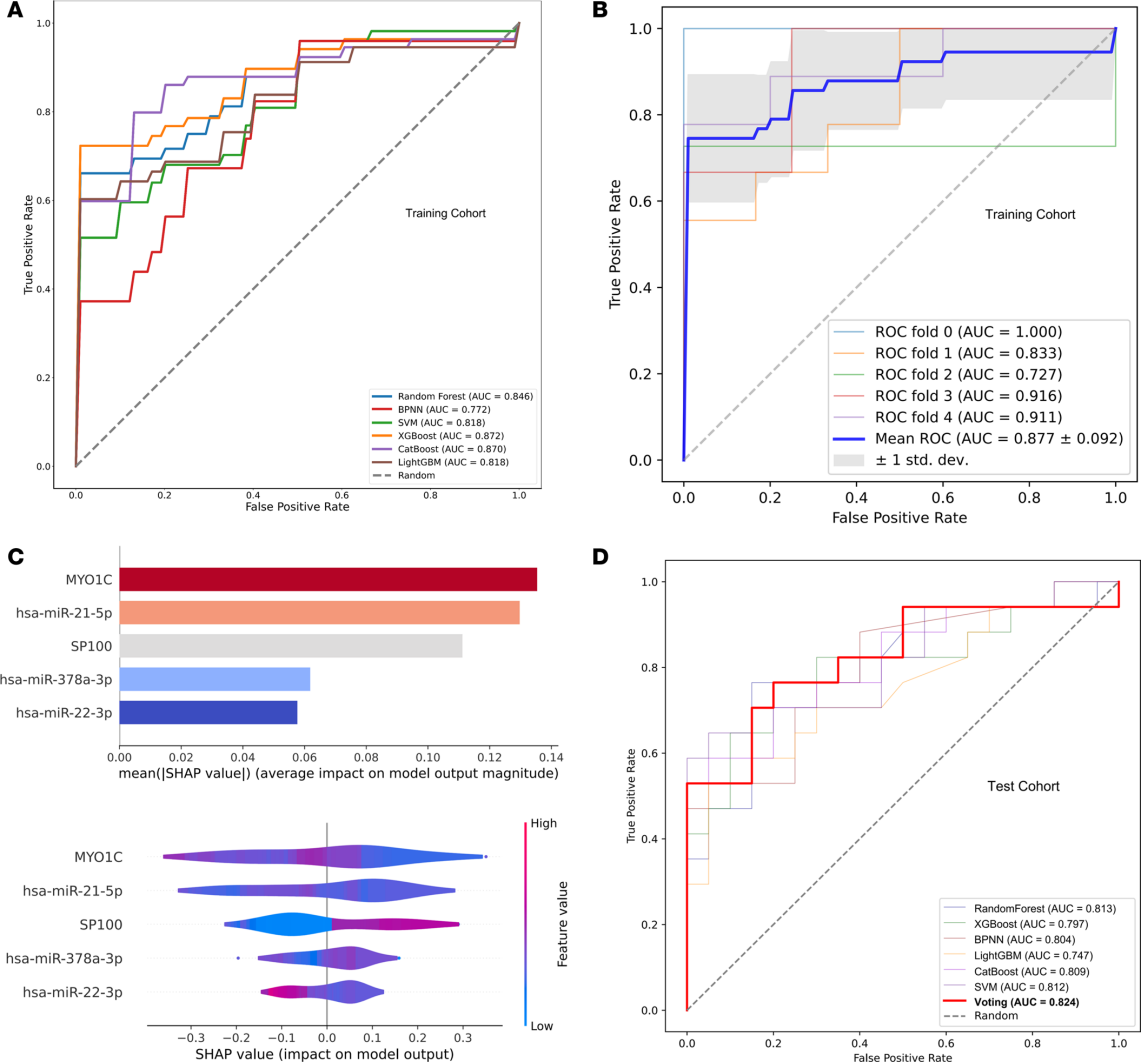
Model construction and feature analysis. (**A**) Summary plot comparing the best ROC curves and average AUC values across the 6 machine learning models in the training cohort. (**B**) ROC curve of the voting ensemble model based on 5-fold cross-validation in the training cohort. (**C**) SHAP analysis of feature importance in the voting ensemble model. The bar plot (top) ranks feature by their average effect on the model output, while the swarm plot (bottom) illustrates the contribution of individual feature values to model predictions. Color intensity (red/blue) indicates the magnitude of influence. (**D**) Summary plot comparing the best ROC curves and average AUC values across the 6 machine learning models in the test cohort.

Five differentially expressed RNAs (*MYO1C*, *SP100*, hsa-miR-21-5p, hsa-miR-22-3p, and hsa-miR-378a-3p) identified by above results in the training cohort were used for further machine learning classification model building steps. Given the importance of avoiding redundancy in feature selection for machine learning, linear regression analysis was used to confirm the absence of significant multicollinearity among the 5 RNA features, thereby supporting their inclusion in the model (Supplemental Figure 1D). Six machine learning algorithms — Random Forest (RF), Support Vector Machine (SVM), Backpropagation Neural Network (BPNN), and 3 gradient boosting methods (XGBoost, LightGBM, and CatBoost) — were employed to develop classification models. The performance of all 6 models is summarized in Figure 1C, with the following AUC values: BPNN, 0.77 (95% CI, 0.68–0.80); SVM, 0.82 (95% CI, 0.73–0.90); RF, 0.85 (95% CI, 0.78–0.91); XGBoost, 0.87 (95% CI, 0.80–0.94); LightGBM, 0.82 (95% CI, 0.75–0.89); and CatBoost, 0.87 (95% CI, 0.80–0.94). Five-fold cross-validation was performed, and SHAP analysis was used to rank feature importance. Details on hyperparameter optimization and feature importance are provided in the Supplemental Figures 2–7. The summary of ROC curves and mean AUC values for all models is presented in Figure 2A and Supplemental Table 2.

To enhance diagnostic accuracy, models with higher AUCs and similar tree-based models (RF, XGBoost, and CatBoost) were integrated using a voting ensemble algorithm. In the training cohort, 5-fold cross-validation demonstrated that the final voting model achieved an AUC of 0.877 (95% CI, 0.794–0.955; Figure 2B), along with an accuracy of 0.741 (95% CI, 0.702-–0.779), precision of 0.784 (95% CI, 0.625–0.944), recall of 0.816 (95% CI, 0.678–0.954), and an F1 score of 0.766 (95% CI, 0.735–0.796; Supplemental Table 2). SHAP analysis identified *MYO1C* mRNA as the most influential feature in the final model (Figure 2C).

### Independent validation and performance evaluation in the time-independent test cohort.

**Figure 3:**
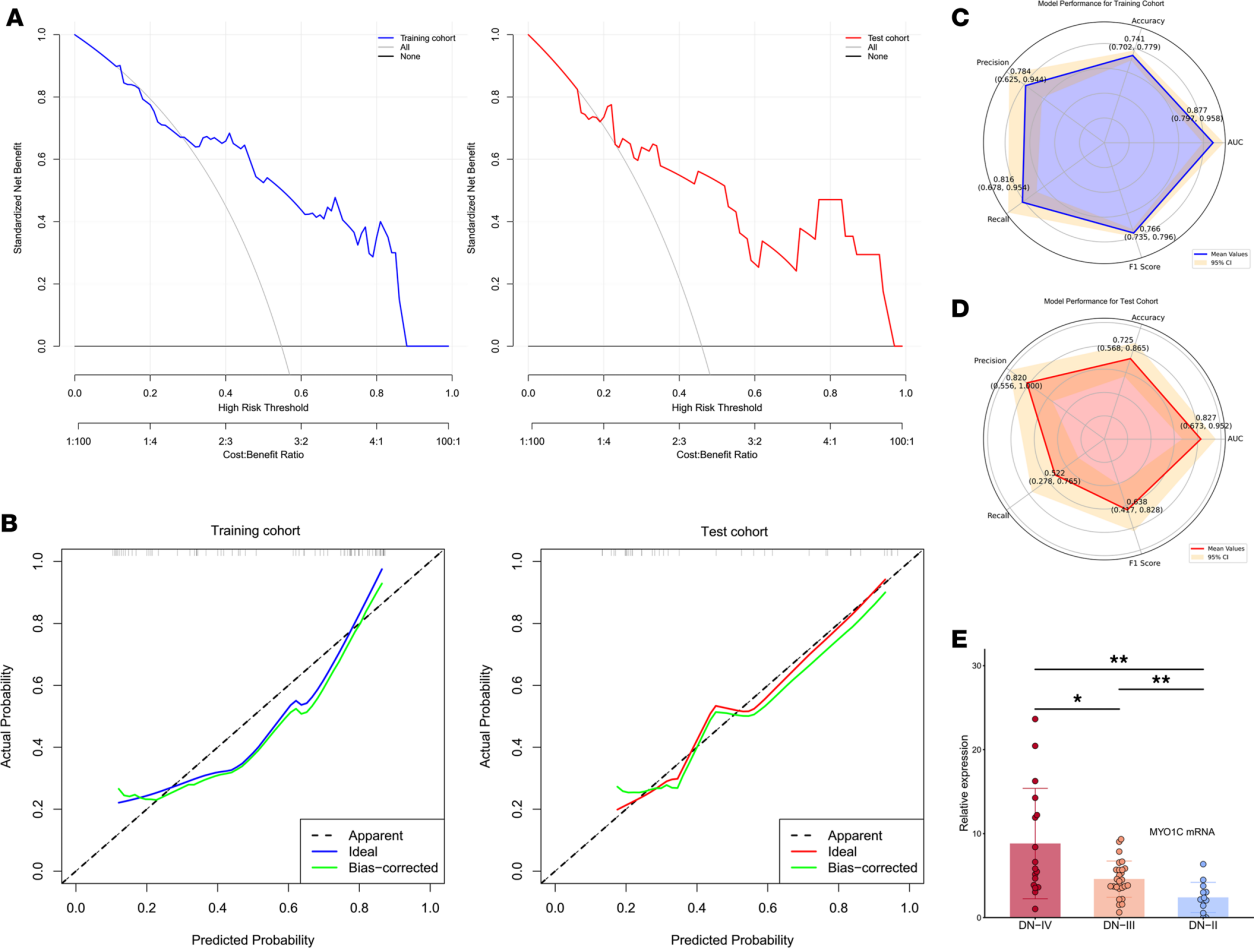
Model performance and analysis across cohorts. (**A**) Decision curve analysis (DCA) for the training cohort and test cohort. (**B**) Calibration curves for the training cohort and test cohort. (**C**) Radar map of model performance for the training cohort. The radar plot illustrates the mean performance metrics (blue line) and their corresponding 95% CI (shaded area in orange) for the accuracy, precision, recall, F1 score, and AUC. (**D**) Radar map model performance for the test cohort. The radar plot illustrates the performance metrics (red line) and their corresponding 95% CI (shaded area in orange) for the accuracy, precision, recall, F1 score, and AUC. (**E**) Bar graph showing *MYO1C* mRNA expression levels across different DN pathological grading groups, presented as mean ± SEM, *n* = 57. Group comparisons were analyzed using 1-way ANOVA with Tukey’s multiple-comparison test. *P < 0.05, **P < 0.01.

The time-independent test cohort consisted of 17 patients with T_2_DN and 20 patients with type 2 diabetes. The baseline characteristics of this cohort are summarized in Supplemental Table 3. Notably, no significant differences were observed between the 2 groups in key clinical parameters, including age, sex, and notably, blood pressure. The expression levels of the 5 candidate RNAs in uEVs were quantified by qPCR (Supplemental Figure 8A) and then applied to the established models. As shown in Figure 2D, the voting model has highest AUC for 0.824 (95% CI, 0.673–0.952) among all models. The calibration and decision curve analyses for both cohorts, shown in Figure 3, A and B, suggest the model’s clinical applicability. The decision curves showed superior net benefit versus default strategies across most thresholds, while the calibration curves indicated well-aligned predictions after bias correction, particularly in the independent test cohort. Compared with the model performance in the training cohort shown in Figure 3C, the model’s performance was further evaluated using test cohort data, yielding an accuracy of 0.725 (95% CI, 0.568–0.865), along with precision, recall, and F1 score, as detailed in Figure 3D and Supplemental Table 4.

Having identified MYO1C mRNA as the most influential feature in the final model, we combined the cohorts to assess its relationship with disease severity. This analysis demonstrated that MYO1C mRNA was significantly upregulated in DN-IV compared with DN-III (*P* = 0.045) and DN-II (*P* < 0.001) stages (Figure 3E), with its expression correlating with UACR (*r* = 0.50, *P* < 0.001; Supplemental Figure 8B) and eGFR (*r* = –0.36, *P* < 0.001; Supplemental Figure 8C). These findings strongly implicate MYO1C in the pathological progression of DN, prompting further investigation into its specific molecular mechanisms.

### Identification of MYO1C as a key candidate for diagnostic and functional validation.

**Figure 4:**
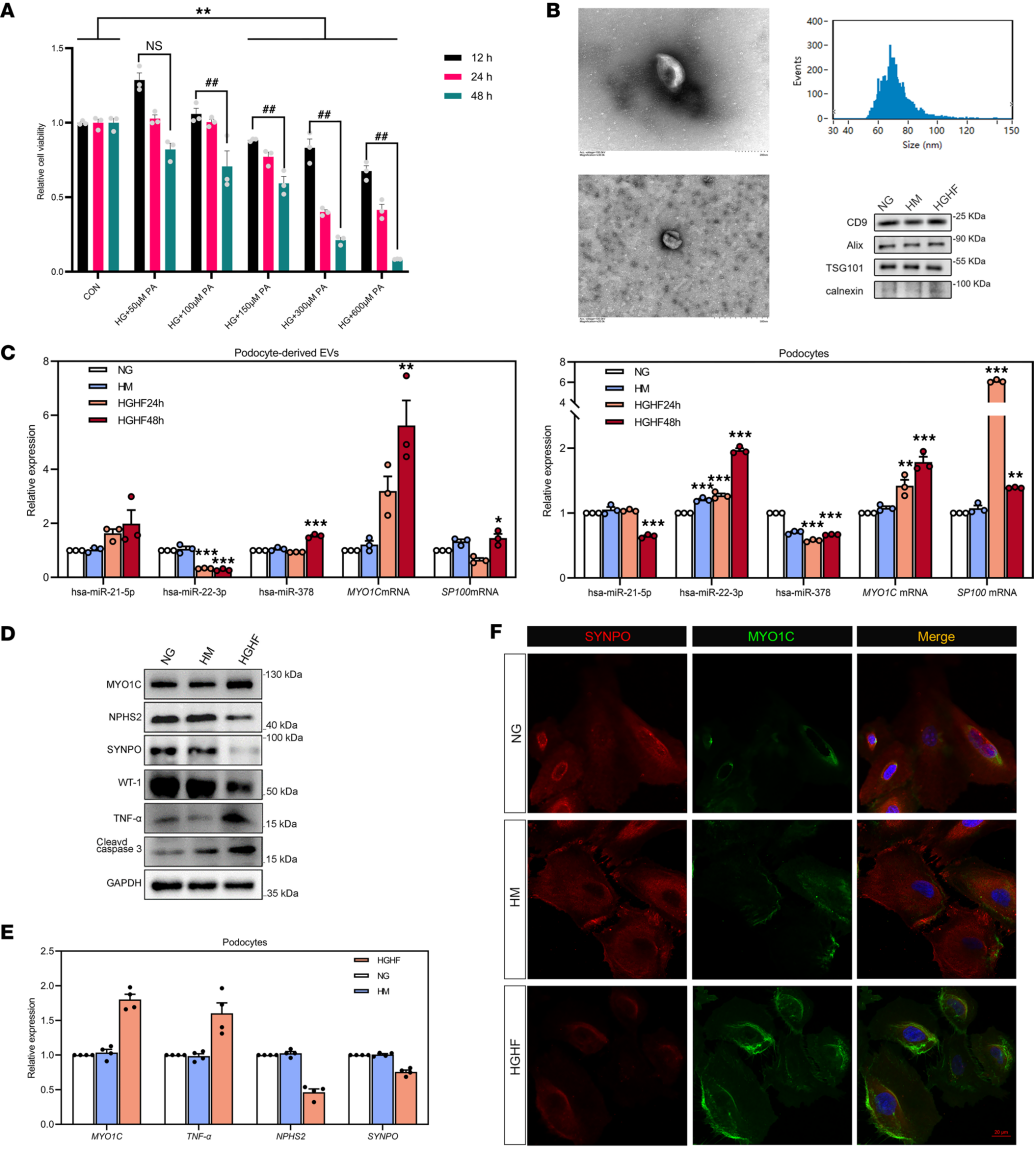
Comprehensive analysis of high glucose and high fat effects on podocyte viability, EVs RNA expression, and molecular expression patterns in HPCs. (**A**) CCK-8 assay evaluating the effect of high glucose and high fat (HGHF) on HPCs cell viability over time. HPCs were treated with BSA (28.8 mM mannitol, control group [CON]) or various concentrations of PA (30 mM glucose, HG+PA group) for different durations. Statistical comparisons: “#” indicates significance between specific groups; “*” indicates significance at 48 hours compared with the CON group. **P < 0.01, ^##^*P* < 0.01. Data are presented as mean ± SEM from 3 independent experiments. (**B**) Characterization of HPCs-derived EVs, including representative TEM images (scale bar: 100 nm), NanoFCM particle size distribution histograms, and immunoblots of EV marker and negative marker proteins. (**C**) qPCR analysis of 5 differential RNAs in HPCs-derived EVs (left) and the corresponding HPCs (right). Data are presented as mean ± SEM from 3 independent experiments. Statistical comparisons were performed using 1-way ANOVA with Tukey’s multiple-comparison test. *P < 0.05, **P < 0.01, ***P < 0.001. (**D**) Immunoblots of specified proteins in HPCs under different treatment conditions. (**E**) qPCR analysis of mRNA expression levels for MYO1C, SYNPO, NPHS2, and TNF-α in HPCs treated with HGHF. Data are from 4 independent experiments. (**F**) Representative confocal images showing SYNPO (red) and MYO1C (green) expression and their colocalization in HPCs under different treatment conditions. Scale bar: 20 μm.

To investigate the role of MYO1C in podocyte injury, we developed a human podocyte (HPCs) model using high-glucose and high-lipid (HGHF) stimulation (Supplemental Figure 9A). CCK-8 assays (Figure 4A) revealed significant reductions in cell viability in HG+150 μM, HG+300 μM, and HG+600 μM groups compared with controls (*P* < 0.01). Due to excessive viability loss at 300 μM and 600 μM concentrations, the condition of 30 mM HG+150 μM for 48 hours was selected for subsequent experiments. TEM and NanoFCM analyses of HPCs-derived extracellular vesicles (hEVs) showed characteristic cup-shaped vesicles (60–90 nm) expressing known EV markers CD 9, Alix, and TSG 101, with low expression of the endoplasmic reticulum protein calnexin (Figure 4B).

qPCR analysis revealed that *MYO1C* mRNA expression in hEVs from the HGHF 48-hour group was significantly upregulated compared with the NG control, consistent with trends observed in uEVs (Figure 4C). Further validation in HPCs confirmed increased *MYO1C* mRNA expression in the HGHF 24-hour and 48-hour groups compared with controls (*P* = 0.0068, *P* = 0.0001; Figure 4C). Immunoblotting analysis demonstrated elevated MYO1C expression in the HGHF group, with concomitant downregulation of podocyte marker proteins NPHS2, SYNPO, and WT-1 and upregulation of inflammatory cytokine TNF-α and apoptosis-related protein cleaved caspase-3 (Figure 4D). qPCR confirmed these findings, showing increased *MYO1C* mRNA (*P* < 0.001), decreased SYNPO and NPHS2 expression, and elevated TNF-α expression in the HGHF group (Figure 4E). Immunofluorescence analysis corroborated these results, showing reduced SYNPO expression and increased MYO1C expression in the HGHF group compared with the NG group (Figure 4F). No significant differences in SYNPO or MYO1C expression were observed between NG and HM groups. These findings indicate that HGHF stimulation induces morphological changes and reduced expression of podocyte marker proteins, along with MYO1C overexpression, suggesting its potential role in podocyte injury.

### Podocyte injury and increased MYO1C expression in high-fat fed db/db mice and patients with T_2_DN.

**Figure 5:**
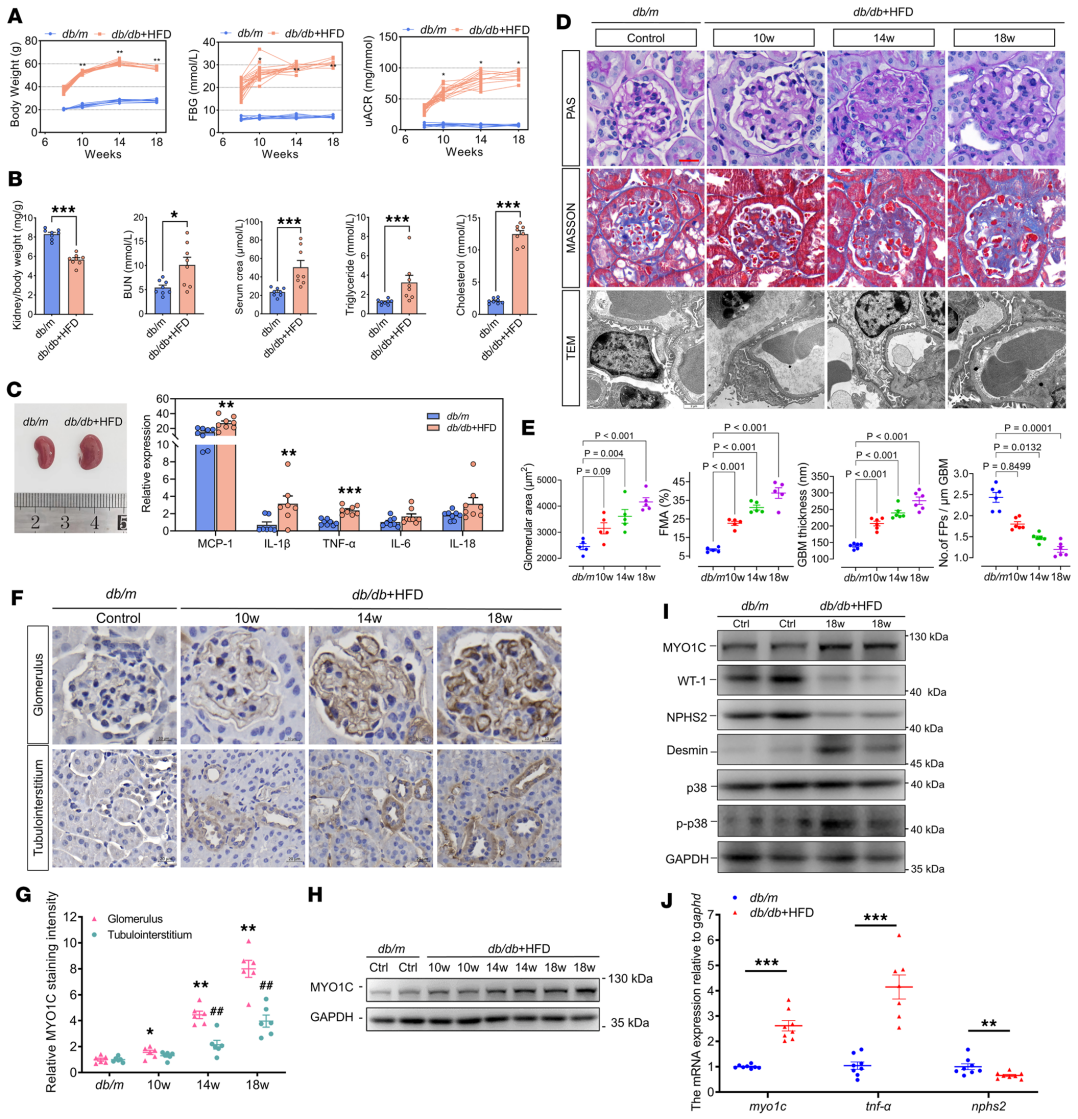
Characterization of renal and systemic changes in *db/m* and *db/db*+HFD mouse models of diabetic nephropathy. (**A**) Body weight, blood glucose levels, and urinary albumin-to-creatinine ratio (UACR) measured at 8, 10, 14, and 18 weeks in *db/m* and *db/db*+HFD mice. (**B**) Postmortem analysis at 18 weeks, including kidney-to-body weight ratio (KW/BW), serum blood urea nitrogen (BUN), serum creatinine, triglycerides, and total cholesterol levels. Data are presented as mean ± SEM, with intergroup comparisons performed using the Mann-Whitney *U* test. *P < 0.05, **P < 0.01, **P < 0.001. (**C**) Macroscopic comparison of kidney size in *db/m* and *db/db*+HFD mice at 18 weeks, along with ELISA measurements of inflammatory cytokines in renal tissue homogenates. Each group: *n* = 8. **P < 0.01, ***P < 0.001. (**D**) Kidney pathology in *db/m* and *db/db*+HFD mice. Representative images of PAS and Masson’s trichrome staining (scale bar: 20 μm), and TEM images (scale bar: 2 μm) of glomerular. (**E**) Quantitative analyses of kidney histology, including mean glomerular tuft area, percentage of PAS-positive area in glomerular tufts, average glomerular basement membrane (GBM) thickness, and number of foot processes per μm of GBM. Each group: *n* = 5–6. Statistical comparisons were performed using 1-way ANOVA with Dunnett’s multiple-comparison test. (**F**) IHC MYO1C staining in kidney tissues from *db/m* and *db/db*+HFD mice. Glomerular regions (scale bar: 10 μm) and tubular interstitial regions (scale bar: 20 μm) are shown. (**G**) Densitometric analysis of MYO1C^+^ staining areas. “*” indicates significant differences in glomerular staining between *db/db*+HFD and *db/m* groups, while “#” indicates differences in tubular interstitial staining. Statistical comparisons were performed using the Mann-Whitney *U* test. *P < 0.05, **P < 0.01, ^##^*P* < 0.01. (**H**) Immunoblots of MYO1C protein expression in kidney tissues of mice at different ages. (**I**) Immunoblots of specified protein expression in kidney tissues of *db/m* and *db/db*+HFD mice at 18 weeks. (**J**) Myo1c, TNF-α, and Nphs2 mRNA levels in kidney tissues of mice, measured by qPCR. Data are presented as mean ± SEM, with intergroup comparisons performed using the Mann-Whitney *U* test. **P < 0.01, ***P < 0.001. Each group: *n* = 6–8.

**Figure 6:**
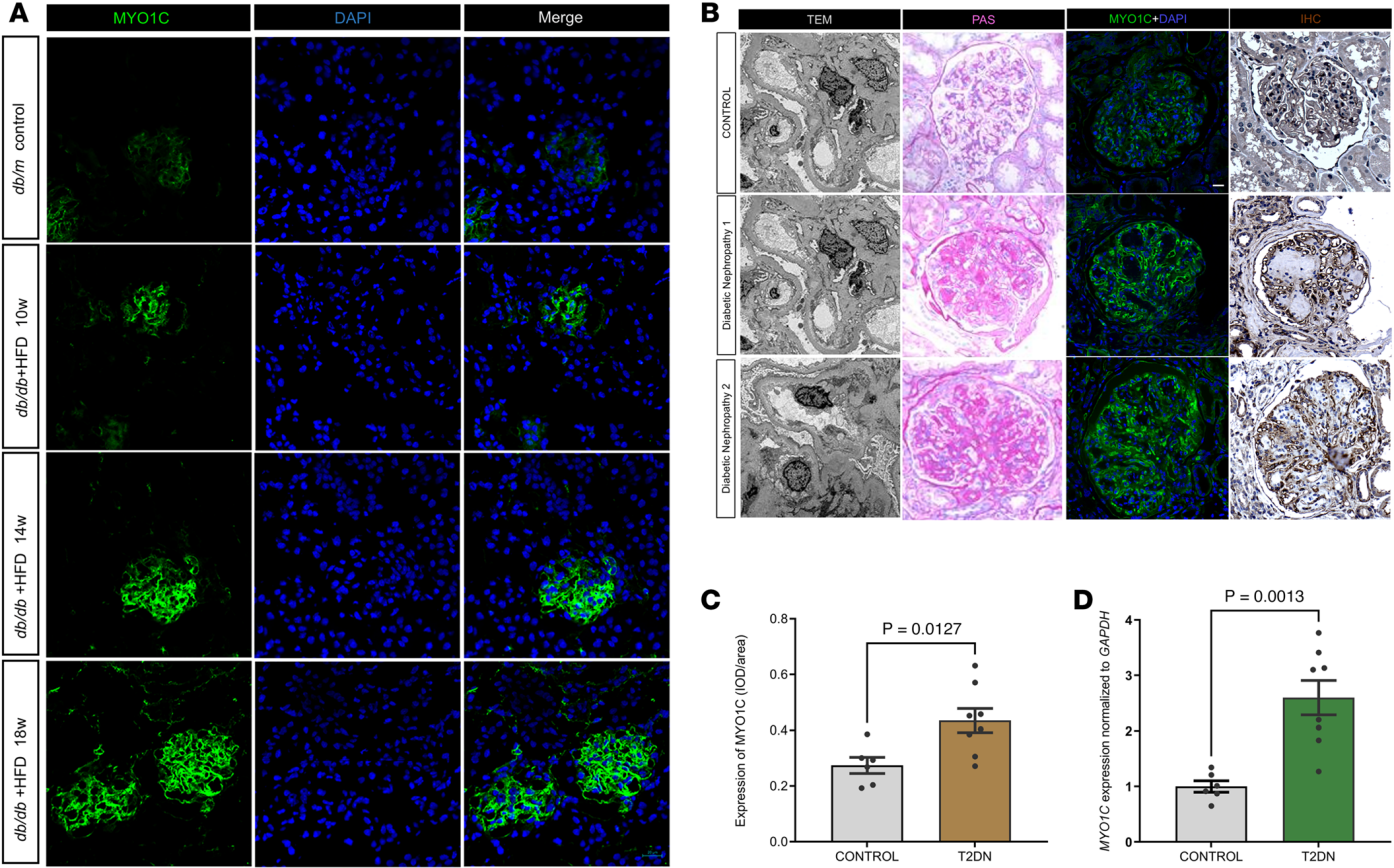
MYO1C expression in mouse and human kidney tissues. (**A**) Representative confocal images of immunofluorescence staining showing MYO1C expression (green) in mouse kidney tissues, with nuclei counterstained using DAPI (blue). Scale bar: 20 μm. (**B**) Kidney pathology in patients with T_2_DN. From left to right: TEM images illustrating podocyte ultrastructural changes (scale bar: 2 μm); PAS staining of kidney sections; immunofluorescence staining of MYO1C on frozen sections (green, Scale bar: 20 μm); and IHC staining of MYO1C on paraffin-embedded sections (scale bar: 20 μm). (**C**) Quantitative analysis of MYO1C IHC staining, presented as the ratio of integrated optical density (IOD) to staining area in kidney tissues. (**D**) *MYO1C* mRNA levels in kidney tissues from control and T_2_DN groups, measured by qPCR. Data are presented as mean ± SEM, with statistical comparisons performed using the Mann-Whitney *U* test (*n* = 6–8).

To examine the expression of MYO1C in vivo, a T_2_DN mouse model was established using high-fat fed *db/db* (*db/db*+HFD) mice. Compared with *db/m* controls, *db/db*+HFD mice exhibited increased body weight at 10, 14, and 18 weeks (*P* < 0.01), along with elevated blood glucose and UACR levels (*P* < 0.05; Figure 5A). At 18 weeks, *db/db*+HFD mice demonstrated reduced kidney-to-body weight ratios (*P* < 0.05), increased serum BUN (*P* < 0.05), Scr (*P* < 0.001), TG, and CHO levels (*P* < 0.001; Figure 5B). ELISA results (Figure 5C) revealed elevated levels of inflammatory cytokines MCP-1 (*P* < 0.01), TNF-α (*P* < 0.001), and IL-1β (*P* < 0.05) in *db/db*+HFD renal homogenates, while IL-18 and IL-6 showed no significant changes. Macroscopically, there were significant gross morphological differences between *db/db*+HFD mice and control kidneys. The *db/db*+HFD mice exhibited glomerular hypertrophy, mesangial expansion, and mild sclerosis in (Figure 5D), and showed significantly larger glomerular tuft areas and increased PAS-positive matrix percentages compared with controls. Electron microscopy demonstrated severe podocyte foot process effacement, reduced podocyte density, and thickened glomerular basement membranes (GBM) in *db/db*+HFD mice (*P* < 0.001; Figure 5E). IHC and immunofluorescence analyses (Figure 5, F and G; and Figure 6A) confirmed elevated MYO1C expression in glomeruli, tubules, and interstitium of *db/db*+HFD mice compared with controls (*P* < 0.05). Immunoblot analysis (Figure 5, H and I) showed a progressive increase in MYO1C protein expression from 10 to 18 weeks, with significant overexpression at 18 weeks (*P* < 0.01). Enhanced p38 MAPK pathway activity was observed, with increased p-p38/p38 ratios (*P* < 0.05), alongside elevated Desmin (*P* < 0.05) and reduced WT-1 and NPHS2 expression (*P* < 0.05). qPCR further validated increased Myo1c and TNF-α expression (*P* < 0.001) and decreased Nphs2 expression (*P* < 0.01, Figure 5J).

To validate MYO1C expression in human DN, renal tissues from 6 nondiabetic controls and 8 patients with T_2_DN were analyzed. PAS and IHC staining showed classical DN pathology in T_2_DN tissues, including glomerular hypertrophy, mesangial expansion, and basement membrane thickening (Figure 6B). Immunofluorescence and qPCR (Figure 6, C and D) confirmed significantly increased MYO1C protein (*P* = 0.013) and mRNA (*P* = 0.001) expression in patients with T_2_DN compared with controls. Together, these findings establish MYO1C as a key molecule associated with podocyte injury and DN pathology in both mouse models and patients.

### MYO1C knockdown alleviates HGHF-induced HPCs injury.

**Figure 7:**
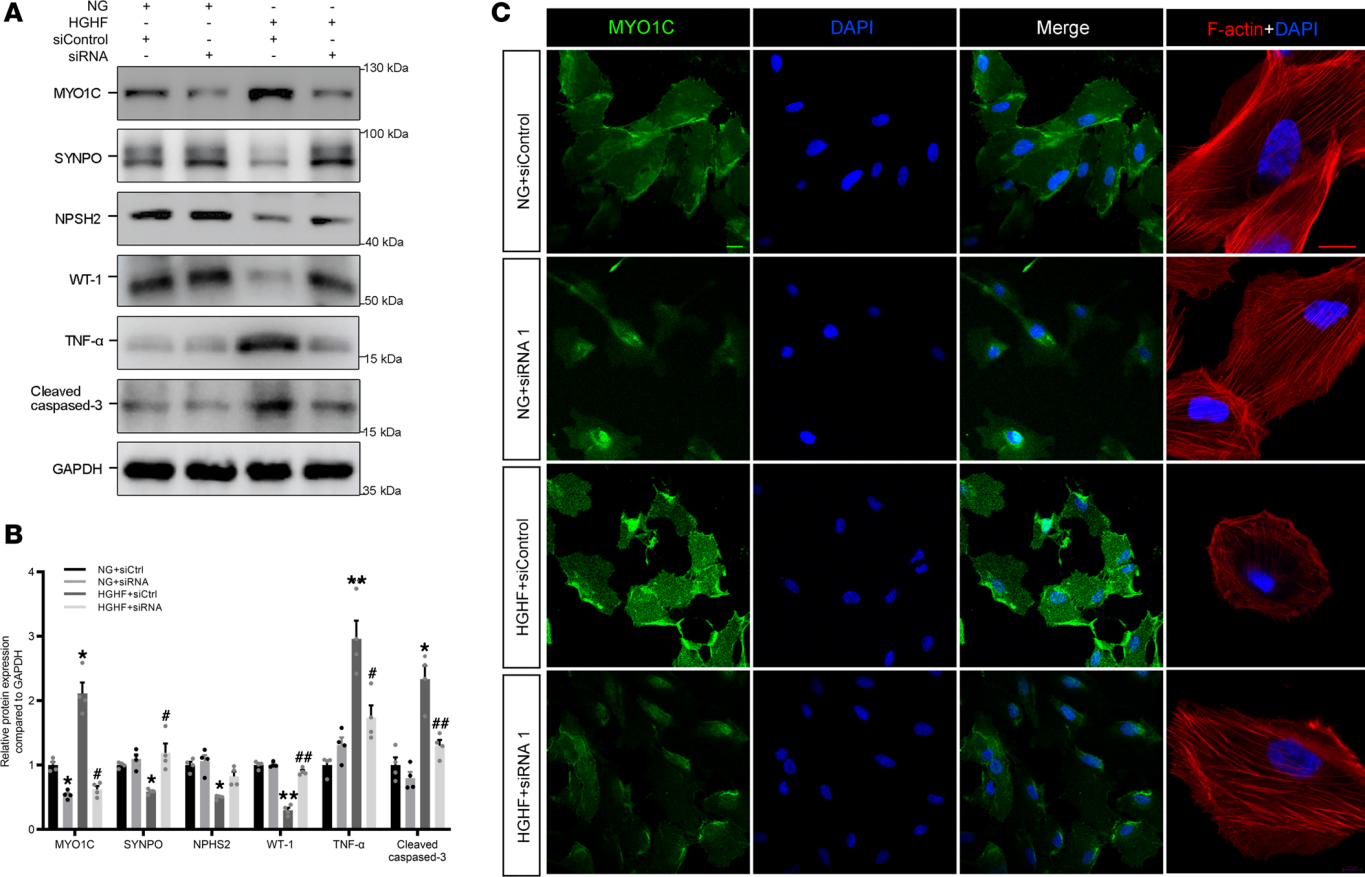
The protective effect of knockdown against HGHF-induced podocyte injury. (**A**) Immunoblots of specified proteins in HPCs following siRNA-mediated knockdown of MYO1C. (**B**) Densitometric analysis of protein expression, normalized to GAPDH from immunoblotting data, presented as mean ± SEM. Statistical comparisons: “*” indicates significance compared with NGs+siControl, and “#” indicates significance compared with HG+siControl. *P < 0.05, **P < 0.01, ^#^*P* < 0.05, ^##^*P* < 0.01. (**C**) Representative confocal images of MYO1C immunofluorescence staining (green) in HPCs, with DAPI (blue) marking nuclei and F-actin (red) showing cytoskeletal structure. Scale bar: 20 μm. Data are from 3 independent experiments.

To investigate the pathogenic role of MYO1C in DN, we attempted to knock down MYO1C expression in HPCs using 3 different MYO1C-specific small interfering RNAs (siRNAs). *MYO1C* mRNA levels were significantly reduced in all 3 siRNA-transfected groups compared with the siControl group (*P* < 0.05), with siRNA-1 showing the greatest knockdown efficiency (Supplemental Figure 9, B and C). Consequently, MYO1C siRNA-1 was selected for further experiments in HPCs under HGHF stimulation. Immunoblot analysis (Figure 7, A and B) demonstrated that MYO1C expression was significantly reduced in NG+siRNA cells compared with NG+siControl cells (*P* < 0.05), without affecting podocyte marker proteins SYNPO, NPHS2, and WT-1 (all *P* > 0.01). In the HGHF+siControl group, MYO1C expression was elevated, while SYNPO, NPHS2, and WT-1 expression were reduced (all *P* < 0.05). Additionally, TNF-α and cleaved caspase-3 levels were significantly increased in the HGHF+siControl group (*P* < 0.05).

Following MYO1C siRNA transfection, the HGHF+siRNA group showed significantly reduced MYO1C expression (*P* < 0.05), increased SYNPO, NPHS2, and WT-1 levels (*P* < 0.05), and decreased TNF-α and cleaved caspase-3 expression (*P* < 0.05) compared with the HGHF+siControl group. These findings indicate that MYO1C knockdown protects against HGHF-induced podocyte injury. Immunofluorescence results (Figure 7C) confirmed reduced MYO1C expression in NG+siRNA and HGHF+siRNA groups compared with their respective siControl groups. In the HGHF+siRNA group, cytoskeletal integrity was better preserved compared with the HGHF+siControl group. These findings demonstrate that MYO1C knockdown alleviates HGHF-induced podocyte injury, highlighting its potential as a therapeutic target for DN.

### Podocyte-specific AAV-mediated MYO1C knockdown alleviates renal podocyte injury in high-fat–fed db/db mice.

**Figure 8:**
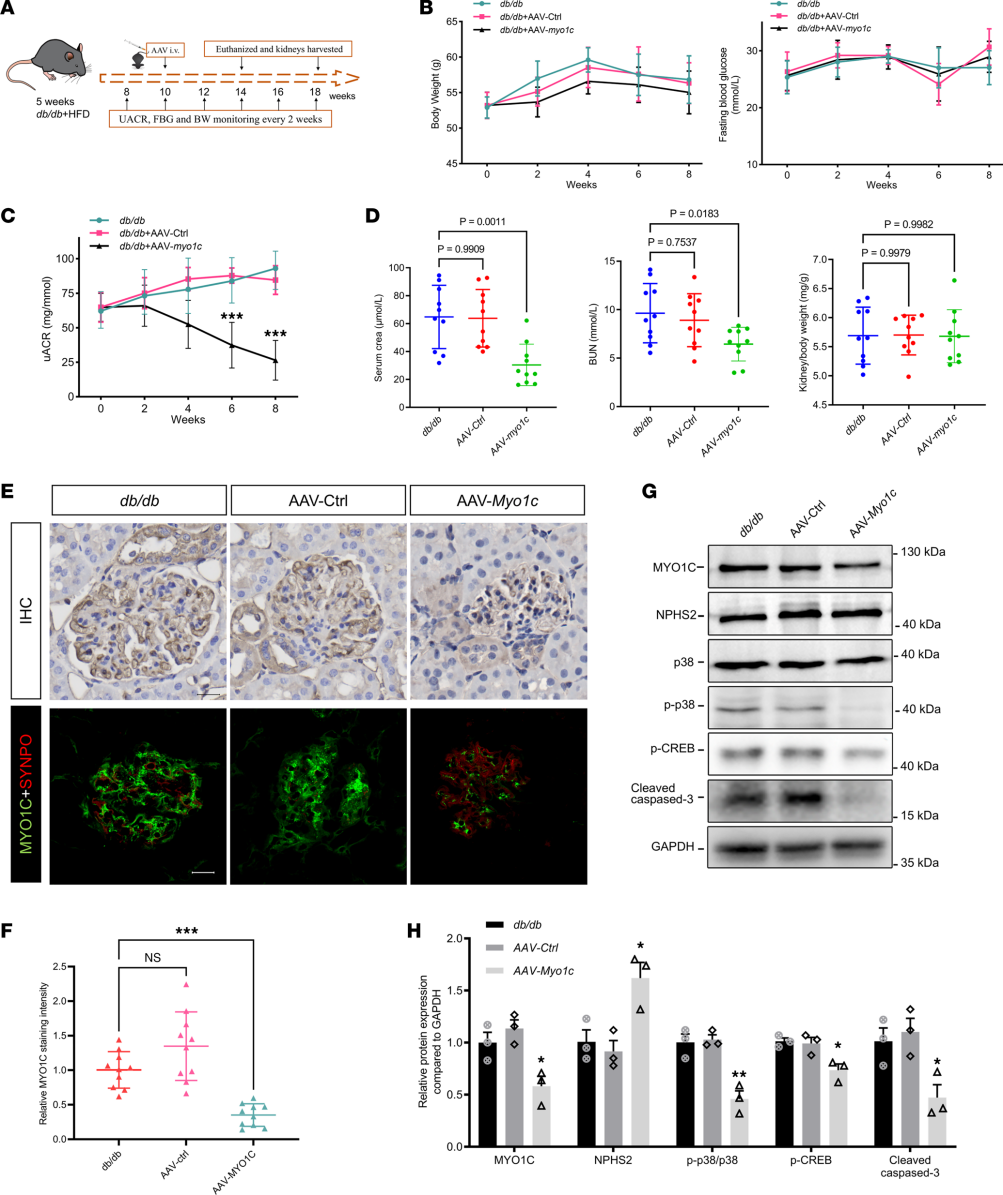
Podocyte-specific knockdown of Myo1c via AAV reduces renal injury in diabetic nephropathy mice. (**A**) Schematic representation of AAV-mediated podocyte-specific knockdown of Myo1c. Ten-week-old *db/db* mice were randomized and received a single i.v. injection of AAV containing a podocyte-specific promoter to knock down MYO1C (AAV-Myo1c) or an empty control vector (AAV-Control). (**B**) Changes in body weight and blood glucose levels at 0, 2, 4, 6, and 8 weeks post-AAV intervention. Data are presented as mean ± SEM (*n* = 10 per group). (**C**) Changes in UACR at 0, 2, 4, 6, and 8 weeks after AAV intervention. Data are presented as mean ± SEM (*n* = 10 per group).***P < 0.001. (**D**) At the 8-week endpoint, serum blood urea nitrogen (BUN), serum creatinine (Scr), and kidney-to-body weight ratio (KW/BW) were measured. Statistical comparisons were performed using 1-way ANOVA with Dunnett’s multiple-comparison test. (**E**) Representative IHC and immunofluorescence images of MYO1C staining in kidney tissues from each group. Scale bar: 20 μm. (**F**) Quantitative analysis of MYO1C IHC staining in glomerular regions. Asterisks indicate significant differences in staining intensity between the AAV-Myo1c group and the *db/db* group. Statistical comparisons were performed using 1-way ANOVA with Dunnett’s multiple-comparison test; ***P < 0.001. (**G**) Immunoblots of MYO1C and other key proteins in kidney tissues from each group. (**H**) Densitometric analysis of protein expression, presented as mean ± SEM (*n* = 6–10 per group). Statistical comparisons were performed using 1-way ANOVA with Dunnett’s multiple-comparison test. *P < 0.05, **P < 0.01.

To investigate the role of MYO1C in renal and podocyte injury in DN, we administered a single tail vein injection of AAV-Myo1c (serotype 2/9) under the podocyte-specific NPHS2 promoter or control AAV (AAV-Ctrl) to 10-week-old high-fat–fed *db/db* mice. After 8 weeks, we assessed metabolic parameters, proteinuria, and renal function (Figure 8A). No significant differences in body weight or blood glucose levels were observed between *db/db*, AAV-Ctrl, and AAV-Myo1c groups throughout the study (*P* > 0.05; Figure 8B). However, UACR levels in the AAV-Myo1c group were significantly reduced at 6 weeks and further decreased by 8 weeks compared with *db/db* and AAV-Ctrl groups (*P* < 0.05; Figure 8C). At the endpoint, BUN (*P* = 0.018) and Scr levels (*P* = 0.001) were significantly lower in the AAV-Myo1c group than in the *db/db* group, while kidney-to-body weight ratios remained unchanged (Figure 8D). These results indicate that podocyte-specific MYO1C knockdown mitigates proteinuria and renal dysfunction independently of glycemic or body weight changes.

Immunofluorescence further demonstrated decreased MYO1C (green) and increased SYNPO (red) expression in the AAV-Myo1c group compared with controls (Figure 8E). There was lower MYO1C expression in glomeruli of AAV-Myo1c mice compared with *db/db* and AAV-Ctrl groups (*P* < 0.001; Figure 8F). Immunoblot analysis corroborated these findings, showing significantly reduced MYO1C protein levels in AAV-Myo1c mice compared with *db/db* and AAV-Ctrl groups (*P* < 0.05; Figure 8, G and H). Additionally, the results indicate a significant decrease in the p-p38/p38 ratio (*P* < 0.01) in the AAV-Myo1c group, accompanied by reduced downstream p-CREB expression (*P* < 0.05), increased NPHS2 levels, and decreased cleaved caspase-3 levels (*P* < 0.05), compared with *db/db* and AAV-Ctrl groups. These findings demonstrate the efficacy of podocyte-specific MYO1C knockdown in alleviating renal podocyte injury and suggest that its protective effects may mediate through the suppression of the p38/CREB pathway and reduction of apoptosis.

### MYO1C mediates podocyte injury via the p38/CREB signaling pathway.

**Figure 9:**
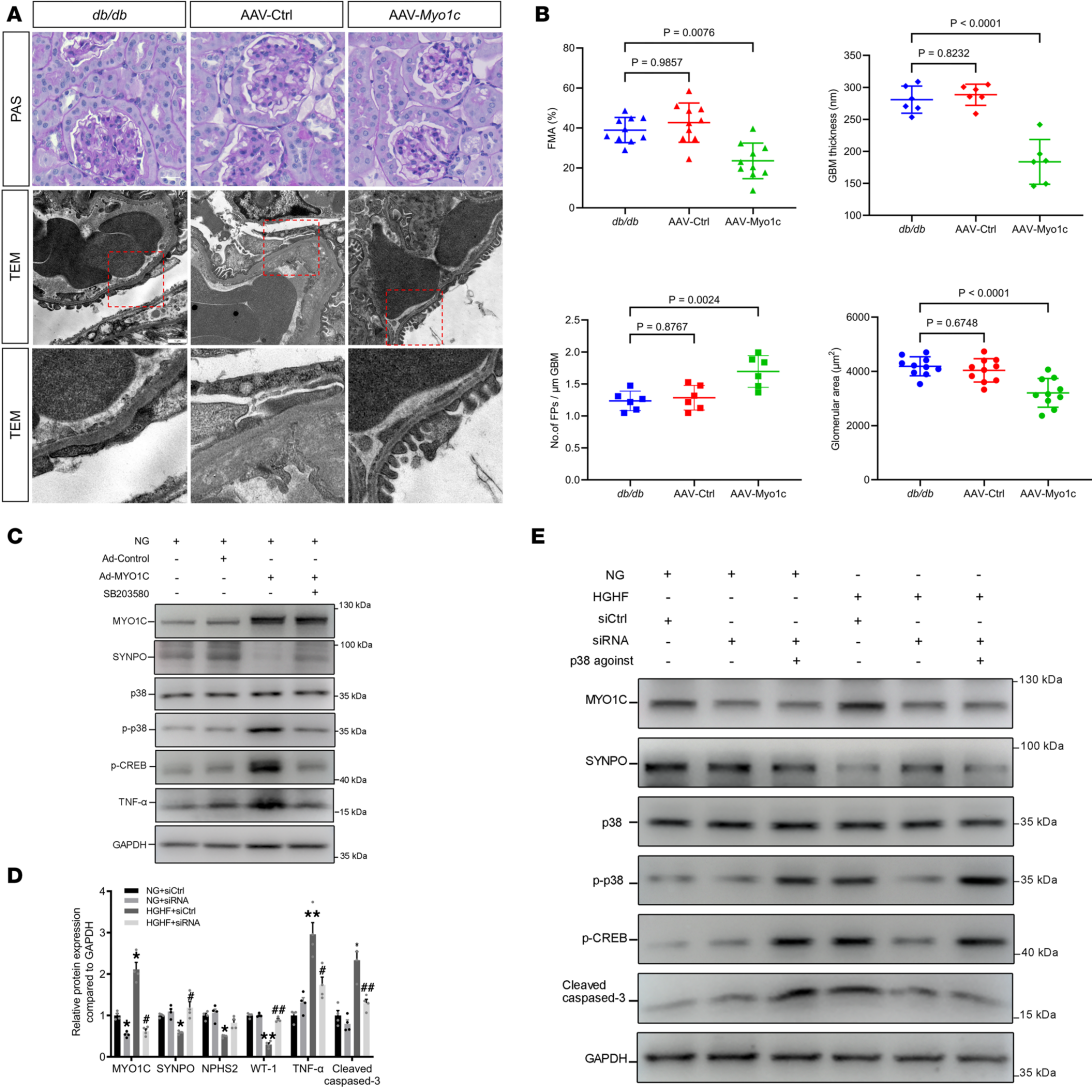
MYO1C mediates HGHF-induced podocyte injury via the p38/p-CREB pathway. (**A**) Representative kidney tissue images showing PAS staining (scale bar: 20 μm) and TEM (scale bar: 2 μm) in different mouse groups. (**B**) Quantitative analysis of kidney pathology, including mean glomerular tuft area, percentage of PAS^+^ area relative to glomerular tuft area, mean glomerular basement membrane (GBM) thickness, and number of foot processes per μm of GBM. Data are presented as mean ± SEM (*n* = 6–10 per group). Statistical comparisons were performed using 1-way ANOVA with Dunnett’s multiple-comparison test. (**C**) Immunoblots of specified proteins in HPCs treated with Ad-MYO1C and the p38 inhibitor SB203580. (**D**) Densitometric analysis of protein expression, normalized to GAPDH from immunoblotting data, presented as mean ± SEM. Statistical comparisons: “*” indicates significance compared with NGs+siControl, and “#” indicates significance compared with HG+siControl. *P < 0.05, **P < 0.01, ^#^*P* < 0.05, ^##^*P* < 0.01. (**E**) Immunoblots of specified proteins in HPCs under HGHF conditions, following MYO1C knockdown via siRNA and treatment with the p38 agonist Dehydrocorydaline.

Inflammatory factors MCP-1, TNF-α, and IL-6 were significantly reduced in the AAV-Myo1c group compared with *db/db* and AAV-Ctrl groups (*P* = 0.035, *P* = 0.004, *P* = 0.018, respectively; Supplemental Figure 10). Pronounced glomerular hypertrophy and mesangial expansion in *db/db* and AAV-Ctrl groups, which were alleviated in AAV-Myo1c mice (Figure 9A). Glomerular tuft areas, PAS-positive matrix, and GBM thickness were significantly reduced in AAV-Myo1c mice (*P* < 0.001; Figure 9B). Electron microscopy further indicated improved podocyte foot process effacement and podocyte density (*P* < 0.001), supporting the role of MYO1C knockdown in mitigating podocyte injury and DN pathology. Additionally, protein docking using AlphaFold-predicted MYO1C and experimentally verified p38 structures suggested a high likelihood of interaction (docking score, –299.79; confidence score, 0.9524; Supplemental Figure 11). Hydrophobic interactions (MYO1C residues 633, 644 with p38 residue 182) and hydrogen bonds (MYO1C residue 636 with p38 residue 180) were identified, corroborated by LigPlot and PyMOL visualization (Supplemental Figure 12, A and B). Coimmunoprecipitation experiments validated MYO1C-p38 binding in vitro (Supplemental Figure 12C).

To investigate whether the p38 MAPK pathway mediates MYO1C-induced podocyte injury, the p38 inhibitor SB203580 was employed. SB203580 inhibits p38 autophosphorylation and substrate phosphorylation without affecting upstream kinase-induced phosphorylation. Immunoblot analysis (Figure 9, C and D) revealed that MYO1C overexpression in NG+Ad-MYO1C cells reduced the expression of podocyte markers NPHS2, SYNPO, and WT-1, while increasing cleaved caspase-3 levels, p-p38/p38 ratios, and p-CREB levels. These effects were partially reversed by SB203580, which reduced p-p38/p38, p-CREB, and TNF-α expression (*P* < 0.05) without altering MYO1C levels.

Building on previous experiments, which showed that MYO1C-siRNA alleviates HGHF-induced podocyte injury by reducing MYO1C expression, we further investigated the role of the p38 pathway using the p38 agonist Dehydrocorydaline. In siRNA-transfected podocytes, MYO1C knockdown mitigated HGHF-induced damage, reducing MYO1C expression and p-p38/p38 ratios, increasing SYNPO levels, and lowering cleaved caspase-3 expression (Supplemental Figure 11, D and E, and Figure 9E). However, treatment with Dehydrocorydaline reversed these protective effects, leading to increased p-p38/p38, p-CREB, and cleaved caspase-3 levels, while reducing SYNPO expression. These findings suggest that the p38/p-CREB signaling pathway plays a crucial role in MYO1C-mediated podocyte injury, with MYO1C knockdown alleviating damage through pathway suppression. Conversely, p38 activation partially negates these protective effects.

## Discussion

DN remains the leading cause of end-stage renal disease, particularly in individuals with T_2_DN represents a major challenge in clinical management. The molecular mechanisms linking metabolic stress to inflammatory activation and podocyte injury in T_2_DN are still not fully understood, despite considerable advances in understanding DN pathogenesis (21). Urinary EVs, originating from the urinary system, can noninvasively reflect kidney status and provide a valuable window for investigating renal pathophysiology (11, 12). Previous studies have reported that, in kidney diseases such as acute kidney injury, uEVs derived from injured kidney cells carry specific molecules, demonstrating their potential as biomarkers (24, 25). In diabetic kidney diseases, several studies have also reported that specific components within uEVs may serve as biomarkers (14, 26, 27). However, these potential biomarkers have not been systematically validated in patients with T_2_DN who have been biopsy diagnosed through pathology, nor have they been supported by functional experiments that demonstrate their direct involvement in key pathological processes of T_2_DN, such as podocyte injury and inflammation. In this study, using a training cohort, we validated the expression of candidate differentially expressed RNAs in uEVs from patients with T_2_DN and developed a machine learning diagnostic model based on these RNAs. The independent test cohort further supports that the final model demonstrates similar good performance and generalizability. Focusing on *MYO1C* mRNA, identified as the most influential feature in the model, we observed its overexpression in both podocyte-derived EVs and podocytes. Moreover, MYO1C was significantly upregulated in both in vitro (high-glucose and high-fat–induced) and in vivo (*db/db* mice with a high-fat diet) T_2_DN models, accompanying podocyte injury, apoptosis, and inflammation. Mechanistically, MYO1C knockdown was shown to alleviate podocyte injury and apoptosis in DN, with evidence suggesting that this effect is mediated through suppression of the p38 MAPK/p-CREB signaling pathway. These findings bridge the gap between diagnostic biomarkers and their mechanistic roles in DN pathology, providing a good foundation for further research into individualized diagnostic and therapeutic strategies for T_2_DN.

Accumulating evidence positions uEVs as an emerging source of noninvasive biomarkers, with their cargo well protected by a lipid bilayer, potentially offering superior predictive value for diseases compared with traditional biomarkers (8, 10). Advances in isolation techniques, omics technologies, and computational methods have improved the sensitivity and specificity of identifying uEV-derived molecules such as proteins and RNAs (10, 14). Proteomic studies have identified promising differentially expressed proteins in uEVs, while transcriptomic analyses have revealed various RNAs as potential diagnostic markers for DN (13, 28–30). Nevertheless, the diagnostic utility of uEV-based markers inT_2_DN requires further validation. In this study, based on prior transcriptomic data (15), we selected 6 candidate RNAs from uEVs (MYO1C, SP100, hsa-miR-21-5p, hsa-miR-22-3p, hsa-miR-378a-3p, and hsa-miR-486-5p) by integrating statistical significance and functional relevance to T_2_DN. MYO1C and SP100 mRNAs were chosen due to their high fold-changes in uEVs from patients with T_2_DN and associations with diabetes and kidney disease pathways identified by KEGG enrichment analysis. The 4 miRNAs were selected based on their marked dysregulation in the ceRNA network and their recognized roles in regulating inflammation and podocyte function. In our training and validation cohort, 5 of these RNAbs, including MYO1C mRNA, SP100 mRNA, hsa-miR-21-5p, hsa-miR-22-3p, and hsa-miR-378a-3p were upregulated in patients with T_2_DN. Although individual RNAs demonstrated only moderate diagnostic accuracy, these findings provided a solid foundation for constructing a multimarker diagnostic model. Recognizing the limitations of single marker approaches, we incorporated machine learning algorithms to enhance model performance. Machine learning methods are increasingly used in biomedical research for their ability to handle high-dimensional data and uncover complex patterns often missed by conventional statistics (31). They facilitate the integration of multi-omics and clinical data, offering insights into disease mechanisms and biomarker discovery (32). In the context of DN, machine learning has been successfully applied to disease diagnosis, progression prediction, and molecular mechanism elucidation (33, 34). We evaluated 6 algorithms, each with distinct strengths: RF was selected for its simplicity and interpretability, SVM for handling high-dimensional data, and gradient boosting models XGBoost, CatBoost, and LightGBM for strong performance on limited sample sizes. Using 5-fold cross-validation and automated hyperparameter tuning, our ensemble voting model achieved an average AUC of 0.877 with balanced performance metrics. This demonstrates the potential of machine learning in diagnostic model development, particularly in small-sample studies. The model also exhibited robust generalizability, maintaining an AUC of 0.824 in a temporally independent test cohort. SHAP analysis identified MYO1C mRNA as the most influential feature in the diagnostic model. Analysis of the pooled cohorts, stratified by pathological stage, confirmed differential expression of MYO1C, with its expression levels also showing statistically significant correlations with the clinical parameters ACR and eGFR. To translate these findings into clinical practice, future work must address technical challenges in standardizing uEVs isolation and marker detection to ensure reproducibility. The noninvasive nature of urine sampling positions uEV-derived MYO1C as a promising candidate for dynamic monitoring in DN management.

Beyond MYO1C, our machine learning approach revealed the synergistic diagnostic value in uEVs RNA comprising MYO1C mRNA, SP100 mRNA, and the miRNAs hsa-miR-21-5p, hsa-miR-22-3p, and hsa-miR-378a-3p. This supports the concept that a combinatorial uEVs RNA panel holds greater diagnostic potential than any single molecule alone. Consistent with previous literature, several miRNAs identified in our cohort have been reported as dysregulated in other studies (34, 35), supporting their potential role as biomarkers for DN. The results also resonate with the general trend in uEV transcriptomic and proteomic research, wherein pathways related to inflammation, immune response, and metabolic stress are consistently enriched (26, 28, 29). However, consistency for individual molecules across different studies remains limited. This variability likely stems from multiple factors, including cohort heterogeneity in disease staging and comorbidities, variables in urinary sample collection and processing protocols, differences in uEVs isolation and normalization methodologies, and variations in sequencing platforms and statistical thresholds (13, 36). These methodological differences may explain why some previous studies failed to detect meaningful changes in these target RNAs. Notably, all T_2_DN cases in our training and test cohorts were biopsy confirmed and stringently screened to exclude other primary or secondary kidney diseases, increasing specificity. The currently used clinical indicators, UACR and eGFR, reflect the functional status of the kidney rather than specific pathological injury mechanisms. These indicators are limited by their delayed response, false-positive/false-negative results due to the presence of normal albuminuric diabetic kidney disease, and are unable to distinguish nondiabetic kidney diseases (37). To further assess disease specificity, future studies should incorporate control groups with nondiabetic kidney diseases and urological conditions, combined with urinary creatinine correction and albuminuria stratification, to distinguish diabetes related signals from background inflammation and proteinuria. From a clinical translation perspective, this 5-marker panel could theoretically complement conventional eGFR and UACR measurements. It might be particularly useful for risk stratification and progression prediction in individuals with preserved eGFR or borderline elevated UACR. Furthermore, the utility may lie more in disease staging, activity assessment, and treatment response monitoring rather than serving as a standalone early diagnostic tool. In our independent test cohort, the panel demonstrated stable discrimination, calibration, and decision curve net benefit; future work should include head-to-head comparisons with eGFR and UACR and quantify clinical utility using reclassification metrics such as net reclassification index. Regarding therapeutic potential, panel components other than MYO1C also show promising pathway associations. For instance, miR-21 promotes fibrosis and inflammation, with antisense oligonucleotides demonstrating renal protective effects in animal model (38). miR-22 is implicated in podocyte inflammatory injury under diabetic conditions, while miR-378 plays a role in modulating renal cell fibrosis, suggesting their potential druggability for kidney injury (39, 40). However, miRNA therapies face challenges including promiscuous targeting, stage-dependent expression, and delivery specificity, requiring advances in tissue targeted delivery systems, dosing optimization, and rigorous toxicological evaluation (41). Nonetheless, a growing body of evidence continues to highlight the multifaceted roles of miRNAs in kidney injury, including their utility as biomarkers and their involvement in pathological mechanisms (42). The other mRNA, *SP100*, is an important transcriptional regulator involved in innate immunity and has been studied primarily in the context of antiviral immunity (43), although its role in kidney disease remains largely unexplored. Collectively, these molecules define a cooperative molecular signature, jointly supporting the diagnostic and translational potential of RNAs in T_2_DN, providing promising directions for future research into combination therapies.

Notably, our study further demonstrated its upregulation in DN models, linking it to podocyte injury, inflammation, and apoptosis. Glucose and lipid metabolism dysregulation induced by hyperglycemia and lipotoxicity are key contributors to podocyte injury in DN (44). In vitro and in vivo models are essential for understanding DN pathophysiology. High glucose is commonly used to replicate diabetes-induced changes, while advanced glycation end products, TGF-β1, angiotensin II, and PA simulate HGHF conditions associated with oxidative stress and lipid toxicity (45). In this study, we explored the effects of combining high glucose with PA to create an HGHF environment for cultured HPCs. This approach aligns with established in vitro models of DN, where 30 mmol/L high glucose and 200 μM PA have been used to induce DN-like conditions in both podocytes and tubular cells (46, 47). PA alone also decreased podocyte viability in a dose- and time-dependent manner, establishing high-fat–stimulated podocytes or tubular cells as useful in vitro models for obesity-associated kidney disease (48). Under HGHF conditions, we observed reduced cell viability and podocyte injury, including decreased expression of marker proteins such as NPHS2, SYNPO, and WT-1 and elevated levels of TNF-α and cleaved caspase-3. Similarly, the *db/db* mouse model combined with a HFD provided an in vivo system to study DN progression. The widely used db/db mouse model, which exhibits progressive DN pathologies such as glomerular hypertrophy and podocyte loss, lacks advanced DN features like severe nodular sclerosis or interstitial fibrosis, limiting its direct relevance to human disease (49, 50). To address this, modifications such as eNOS-deficient strains (db/db eNOS^–/–^ and STZ-eNOS^–/–^), or combining db/db or STZ-induced models with HFD or high-protein diets (51), have been utilized to better mimic human DN. Studies show that db/db+HFD mice exhibit aggravated type 2 diabetes complications, including cardiomyopathy and lipid deposition (52, 53), along with marked increases in renal dysfunction markers and histopathological abnormalities (51, 54). Similarly, study demonstrated enhanced tubular injury and renal damage using *db/db*+HFD mice and validated the findings in an HGHF-induced tubular cell model (55). Consistent with these reports, our study observed that *db/db*+HFD mice displayed marked increases in body weight, UACR, and renal injury, with ultrastructural analysis revealing podocyte depletion and foot process effacement. Immunoblotting confirmed decreased expression of podocyte markers, highlighting the utility of the *db/db*+HFD model as a translational platform for investigating DN pathophysiology.

Focusing on MYO1C, we observed upregulated in the HPCs by HGHF and in the kidneys of *db/db*+HFD mice, correlating with inflammation and apoptosis. Myosins are actin-based motor proteins that constitute a diverse superfamily essential for muscle contraction and numerous cellular functions; they are categorized into distinct classes based on motor-domain homology and tail-domain architecture (56). MYO1C, an unconventional class I myosin, is the first mammalian single-headed myosin to be purified and sequenced. Unlike conventional myosins, MYO1C has a single-headed structure and does not form bipolar filaments. Structurally, it comprises 3 main domains: the motor domain, which converts ATP hydrolysis into mechanical force for actin interaction; the neck domain, containing calmodulin-binding sites that amplify motor-generated movement and regulate activity; and the tail domain, featuring a Pleckstrin Homology domain that mediates lipid membrane interactions, enabling MYO1C’s role in diverse cellular processes (16, 57, 58). The structural and functional characteristics of MYO1C highlight its pivotal role in cellular dynamics and its potential involvement in human diseases. Recent evidence has revealed multifaceted functions of MYO1C in various diseases, implicating its role in cellular processes potentially relevant to DN. MYO1C interacts with insulin-stimulated GLUT4, and its siRNA-mediated knockdown has been shown to inhibit GLUT4 translocation in adipocytes via CaMKII-dependent phosphorylation (59). Additionally, MYO1C is involved in autophagy regulation; loss of functional MYO1C disrupts cellular cholesterol distribution and impairs selective autophagic pathways (60). In vascular endothelial cells, MYO1C enhances the secretion of von Willebrand factor, thereby modulating hemostasis and inflammatory responses (61). Within podocytes, MYO1C helps maintain structural integrity by interacting with slit diaphragm proteins such as nephrin and Neph1 (19). Furthermore, recessive variants in MYO1C have been identified as a novel genetic cause of nephrotic syndrome in children (62). Recent work also indicates that MYO1C modulates TGF-β signaling, with podocyte-specific KO ameliorating glomerular fibrosis in multiple mouse models (20). Despite these advances, the direct role of MYO1C in DN remains unexplored. Our study demonstrated MYO1C overexpression in hEVs and kidney tissues under DN conditions, including HGHF-treated podocytes and *db/db*+HFD mice. Elevated MYO1C levels were associated with podocyte injury, characterized by reduced marker proteins, increased apoptosis, and heightened inflammatory responses. Moreover, biopsy samples from patients with T_2_DN revealed MYO1C upregulation, suggesting its potential as a biomarker for DN diagnosis. In DN, p38 MAPK activation is closely associated with inflammation, apoptosis, and renal injury (23). High glucose and advanced glycation end products trigger its activation in renal cells, engaging downstream effector CREB to regulate transcription and inflammatory responses, and its modulation has demonstrated protective effects against DN pathology (63, 64). A recent study on brown adipose tissue reported that MYO1C inhibition in vitro led to the activation of protein kinase A and its downstream substrate p38 (65). In contrast, our findings demonstrate that overexpression in podocytes induces injury, increases p38 phosphorylation, and upregulates p-CREB and TNF-α. Conversely, MYO1C knockdown in HGHF-stimulated HPCs restores podocyte markers, reduces apoptosis, and attenuates TNF-α expression, with decreased p38 activity and a reduced p-p38/p38 ratio. Similar protective effects were observed in vivo, where MYO1C knockdown improved renal function, reduced UACR, and alleviated renal pathology, inflammation, and podocyte injury, alongside reduced p-p38 and p-CREB expression. However, it is important to note that prior reports indicate MYO1C depletion can alter short actin arrays in certain cell types and may affect intracellular organelles and other cellular processes (16, 58). In zebrafish, genetic loss of MYO1C can lead to developmental abnormalities and impaired glomerular filtration function (66). It has also been suggested that MYO1C participates in autophagy-lysosomal fusion, as reduced colocalization of MYO1C and F-actin with LC3 or LAMP1 following its KO can disrupt autophagosome-lysosome fusion (67). These functional differences may reflect variations in cellular context and experimental sensitivity. Given the pleiotropic roles of MYO1C in organelle trafficking and membrane-cytoskeleton linkage, any future attempts to modulate its function will require careful assessment of its effects on cytoskeletal organization, organelle dynamics, and cell viability in podocytes and other relevant cell types. To functionally validate the role of p38 MAPK signaling in MYO1C mediated podocyte injury, we employed the inhibitor SB203580 and the agonist Dehydrocorydaline. It has been reported that SB203580 maintains the integrity of podocyte slit diaphragms under HG conditions and reduce proteinuria in STZ-induced diabetic mice (68). Dehydrocorydaline, a natural alkaloid from Corydalis, is known to enhance the p38 phosphorylation and pathway activity (69). Consistent with these findings, in our study, MYO1C overexpression combined with SB203580 treatment resulted in decreased expression of downstream transcription factors p-CREB and TNF-α, while MYO1C expression remained unaffected. Conversely, MYO1C knockdown followed by treatment with Dehydrocorydaline led to increased p-p38 phosphorylation, elevated p-CREB and cleaved caspase-3 levels, and exacerbated podocyte injury. This intervention partially reversed the protective effects of MYO1C knockdown against podocyte damage. These results collectively suggest that the p38 MAPK pathway plays a mediating role in the relationship between MYO1C expression and podocyte injury. Taken together, these findings indicate that MYO1C acts beyond structural roles and actively engages inflammatory signaling. This functional distinction delineates a more precise therapeutic window, indicating that selective modulation of disease-associated MYO1C signaling may provide efficacy while minimizing adverse effects on normal cellular functions.

In total, this study helps bridge the gap between the diagnostic potential of uEVs RNA biomarkers and their functional relevance in DN pathogenesis. By validating candidate RNAs and elucidating the role of MYO1C, this research lays the foundation for future advancements in both diagnostics and mechanistic research. MYO1C may serve as a valuable biomarker and a subject for further investigation into the molecular pathways underlying T_2_DN. Given its involvement in podocyte injury and p38 MAPK signaling, future studies that modulate MYO1C using genetic or pharmacological approaches may help clarify its contribution to podocyte dysfunction and disease progression. Nevertheless, since MYO1C participates in diverse cellular processes, additional work is required to define its broader biological effects and the implications for cellular homeostasis. It should be noted that the diagnostic model presented here was validated in a single-center cohort with a limited sample size. Larger, multicenter studies are essential to confirm its clinical applicability. Further investigation into the relationship between MYO1C expression and different stages of DN, as well as its association with disease progression and outcomes, will be crucial to fully assess its role as a biomarker and its pathophysiological relevance.

## Methods

### Sex as a biological variable.

Male mice were selected in the animal experiment part of this study. The applicability of these findings to female rodents is unknown. In analyses involving human samples, sex was not considered a biological variable and stratification was not considered due to sample size limitations.

### Selection of candidate RNA biomarkers.

Key RNA biomarkers were selected based on our previous transcriptomic analysis of uEVs from 12 patients with T_2_DN and 12 controls with type 2 diabetes (15). The transcriptomic analysis was conducted prior to all validation and mechanistic experiments in this study. Additional details are available in the Supplemental Methods.

### Patient cohorts, uEVs isolation and characterization.

Patient enrollment, sample collection, and uEVs isolation followed our previously established protocols (15). Briefly, the training cohort included biopsy-confirmed patients with T_2_DN and matched type 2 diabetes patient controls, enrolled between October 2021 and April 2023, from whom morning urine samples (100–200 mL) were collected prospectively. The independent test cohort consisted of patients enrolled between April 2024 and April 2025, with urine samples retrieved from the hospital biobank and the National Human Genetic Resources Sharing Service Platform (2005DKA21300). Additional details and uEV isolation and characterization are available in the Supplemental Methods.

### EVs RNA extraction and qPCR.

Total RNA was extracted from PBS-resuspended EVs samples using the miRNeasy Serum/Plasma Kit (217184, QIAGEN, Germany) following the manufacturer’s instructions, including an DNase digestion using the RNase-Free DNase Set (79254, QIAGEN, Germany) to minimize genomic DNA contamination. RNA concentrations were quantified using the Qubit 4.0 fluorometer with Qubit RNA Assay Kits (Q32852, ThermoFisher). For mRNA, complementary DNA (cDNA) was synthesized via reverse transcription using the PrimeScript RT Master Mix (RR036A, Takara, Japan). miRNA cDNA was prepared using the miReute Enhanced miRNA First-Strand Synthesis Kit (KR211, TIANGEN, China). qPCR was performed using the Premix Ex Taq ROX plus (RR39LR,Takara, Japan) to measure the relative expression levels of target genes. Primers and probes for mRNA and miRNA were designed and synthesized by Generalbiol (Chuzhou, China) and are detailed in Supplemental Table 5. Positive and negative controls were included in every run for quality assurance. *GAPDH* mRNA served as the internal control for mRNA, *U6* for miRNA, while cel-mir-39 was used as the external reference for miRNA. Based on sequencing stability, hsa-miR-125b-5p was selected as the internal control for miRNA analysis. Relative expression levels were calculated using the 2^–ΔΔCt^ method.

### Machine learning model development and feature analysis.

Data preprocessing was conducted using the StandardScaler() function to standardize features. Spearman’s correlation coefficients were calculated, and a correlation matrix for all features was created. To identify and exclude highly collinear features, pairwise relationships between variables were visualized using the sns.pairplot() function. A predefined threshold of Spearman’s correlation coefficient (*r* > 0.8) was used to flag and remove strongly collinear features. Additional details are available in the Supplemental Methods.

### External validation with the time-independent test cohort.

To assess the generalizability and real-world applicability of the model, an external validation was conducted on a time-independent test cohort. The model, fixed in its parameters after fitting on the training cohort, was applied to predict outcomes for this new dataset. Performance was comprehensively evaluated, and the results were presented in a radar plot. Additionally, the calibration curve was used to assess the agreement between predicted probabilities and actual outcomes in R using the *rms* package, which is essential for risk stratification. Decision curve analysis was also conducted to quantify the clinical net benefit of using the model across various probability thresholds, supporting its utility in informed decision-making, implemented in R using the *rmda* package.​

### Cell culture.

Conditionally immortalized HPCs, provided by Jinling Clinical Medical College of Nanjing Medical University (Najing City, Jiangsu Province, China), were cultured following previously established protocols (63, 70). HPCs were cultured in RPMI 1640 medium (Gibco, ThermoFisher, USA) with 10% FBS (Exosome Depleted Fetal Bovine Serum,VivaCell, XP Biomed, China) at 33°C for 10–14 days to induce differentiation. After differentiation, cells were transferred to a 37°C incubator, and the medium was replaced with the appropriate culture conditions for further experiments. The palmitic acid (PA) stock solution (10 mM, SYSJ-KJ002, Xi’an Kunchuang Biotechnology, China) was diluted in high-glucose medium to generate high-glucose and high-fat (HGHF) conditions (71). Additional details are available in the Supplemental Methods.

### siRNA, adenovirus transfection and chemical modulation in HPCs.

To identify the most effective siRNA for MYO1C knockdown, HPCs were randomly divided into 5 groups: Control (normal culture medium without antibiotics), siRNA+CY3 (fluorescent siRNA control), siRNA-1, siRNA-2, and siRNA-3. The siRNAs used in this study (detailed sequences provided in Supplemental Table 6) were constructed by Hanbio Biotechnology Co. Ltd. (Shanghai, China). Additional details and uEV isolation and characterization are available in the Supplemental Methods.

### Western immunoblot analysis.

Protein extraction from cultured HPCs or mouse kidney tissues was performed using the Minute Total Protein Extraction Kit for Animal Cells/Tissues (SN-002, Invent Biotechnologies, CHINA), following the manufacturer’s protocol. The standard immunoblotting procedure included preparation of polyacrylamide gels, sample loading, electrophoresis, membrane transfer, blocking, primary and secondary antibody incubation, and visualization of protein bands. Additional details are available in the Supplemental Methods. Protein markers (26617, Thermo Fisher Scientific) were used for validation. The following primary antibodies were employed: MYO1C (ab194828, Abcam), CD 9 (ab263019, Abcam), Alix (ab275377, Abcam), TSG 101 (ab125011, Abcam), Calnexin (ab133615, Abcam), NPHS2(BS6597R, Bioss), SYNPO(21064-1-AP, Proteintech), WT-1(sc7385, Santa), TNF-α(A0277, ABclonal), cleaved caspased-3 (9661S, CST), desmin (sc23879, Santa), p38(8690S, CST), p-p38(4511S, CST), and p-CREB(9198S, CST). GAPDH (Goodhere Biotechnology) was used as the loading control. Secondary antibodies were purchased from Abbkine Biotechnology (A21020, Wuhan, China). The relative intensity of protein bands was quantified using Image *J* software.

### Mouse model and measurements.

Five-week-old male SPF-grade *db/db* mice (*n* = 54) and *db/m* control mice (*n* = 10) were obtained from Jiangsu Jicui Yaokang Technology Co. Ltd. (Nanjing, China). All mice were housed in SPF individually ventilated cages (3–4 mice per cage) at the Zhengzhou University Animal Experiment Center. The environment was maintained at a temperature of 20°C–22°C with a 12-hour natural light-dark cycle. The *db/db* mice were fed a high-fat diet (HFD, 60 kcal% fat content), referred to as the *db/db*+HFD group. The *db/m* mice were fed a standard diet. Mice had free access to water, except during blood glucose measurements, and bedding was changed regularly. Additional details are available in the Supplemental Methods.

### ELISA.

Mouse kidney tissue was accurately weighed and homogenized on ice with 0.9% saline containing a protease inhibitor cocktail (P6730, Solarbio, China) to prepare a 10% homogenate (1 mg tissue per 9 μL saline). The homogenate was centrifuged at 1,200–1,500*g* for 10 minutes, and the supernatant was collected for further analysis. ELISA measurements were conducted according to the protocols provided in the respective assay kit manuals (MultiSciences, Hangzhou, China).

### Cell and kidney tissue samples RNA extraction and qPCR.

Total RNA from HPCs was isolated using the RNeasy Mini Kit (QIAGEN, USA), while RNA from kidney tissue was extracted with the FastPure Cell/Tissue Total RNA Isolation Kit (VAZYME, China), following the manufacturer’s protocols. Additional details are available in the Supplemental Methods.

### Human renal biopsy specimens.

Human renal biopsy specimens were obtained from the hospital biobank and the National Human Genetic Resources Sharing Service Platform (2005DKA21300). Samples included adjacent nontumor kidney tissues from 6 patients undergoing nephrectomy for renal parenchymal tumors and renal biopsy tissues from 8 patients with T_2_DN.

### Histological analysis, IHC staining, immunofluorescence analysis, and protein interaction prediction.

Detailed protocols are provided in the Supplemental Methods.

### Statistics.

Statistical comparisons between 2 groups were conducted using the Mann-Whitney *U* test (2-tailed) with GraphPad Prism 8.0 (GraphPad Soft-ware, USA). A post hoc power analysis indicated that, with a sample size of 74 in the training cohort, the study had 80% power to detect a mean difference in uEV MYO1C mRNA levels between T_2_DN and type 2 diabetes groups (Cohen’s *d* = 0.67) at a 2-sided α of 0.05. The 95% CI for the machine learning model metrics were derived from 1,000 bootstrap samples of the training and testing sets. One-way ANOVA followed by Tukey’s test was employed for statistical comparisons between 3 or more groups. All quantitative data are expressed as the mean ± SD. *P* < 0.05 was considered to be statistically significant.

### Study approval.

The cohort study adhered to the Declaration of Helsinki and received approval from the Institutional Ethics Committee of The First Affliated Hospital of Zhengzhou University (no. KY-2018-LW-66). All animal experiments and methods were performed following the relevant guidelines and regulations approved by the Animal Experimentation Committee of Zhengzhou University [no. ZZU-LAC20231201(05)]. The use of renal biopsy tissues in this study was approved by the Institutional Ethics Committee of The First Affliated Hospital of Zhengzhou University, with a waiver of informed consent (no. 2023-KY-0656-002).

### Data availability.

Values for all data points in graphs are reported in the Supporting Data Values file. Additional information or clarification is available from the corresponding author upon reasonable request.

## Author contributions

ZZ, JD, and ZL designed this study, performed the experiments, and made major contributions in writing the manuscript. ZZ, SZ, QY, FL, YL, JR, SP, Zhenjie Liu, and DL participated in collecting data and performing the statistical analysis. JD and Zhangsuo Liu made substantial contributions to the design and critical revision of the manuscript. All authors have read and agreed to the published version of the manuscript.

## Funding support

- Natural Science Foundation of China (joint project no. U21A20348).
- National Natural Science Young Scientists Foundation of China (nos. 82103916 and 82200798).
- Natural Science Foundation of Henan Province Excellent Young Scientists Fund Program (no. 202300410363).
- Henan Province Medical Science and Technology Joint Project (LHGJ20250295)

## Supplementary Material

Supplemental data

Unedited blot and gel images

Supporting data values

## Version 1 01/22/2026

In-Press Preview

## Version 2 03/09/2026

Electronic publication
