# Supplementary material for: MYO1C is a urinary extracellular vesicle biomarker and mediator of podocyte injury in diabetic nephropathy
Source: JCI Insight. 2026 Jan 22;11(5):e194604. doi: 10.1172/jci.insight.194604 (PMC13041675; doi:10.1172/jci.insight.194604)
Supplement: Supplemental data [file jciinsight-11-194604-s265.pdf]

Supplementary Method Materials

1. Primer sequence of probe qRT-PCR

Gene	Primer sequence (5'--3')
GAPDH-F	TCAGCCGCATCTTCTTTTGC
GAPDH-P	TCGCCAGCCGAGCCACATC
GAPDH-R1	GCCCAATACGACCAAATCCG
U6-RT	AACGCTTCACGAATTTGCGT
U6-S	CTCGCTTCGGCAGCACA
U6-A	AACGCTTCACGAATTTGCGT
U6 probe	AGAAGATTAGCATGGCCCCTGCGCA
cel-mir-39-RT	CAGTGCAGGGTCCGAGGTCAGAGCCACCTGGGCAATTTTTTTTT TCAAGCTGATT
cel-mir-39-F	CGCTCACCGGGTGTAAATC
MYO1C-F	CCCCTTCTTTCTGCCTTAAGA
MYO1C-R	GGGTTACAGGCGTGAGC
MYO1C-P	CACCATGCCCAGCCTAAAAGGAC
SP100-F	GGTGATGAAGTACTCTGTACAACA
SP100-R	AAAAAACTTTTAGGTTCAGGGGTAT
SP100-P	TTTGTTATATAGGGAAACTCTTGTCACGGG
hsa-miR-21-5p-RT	CAGTGCAGGGTCCGAGGTCAGAGCCACCTGGGCAATTTTTTTTT TTCAACA
hsa-miR-21-5p-F	CTCCACGTAGCTTATCAGACTGA
hsa-miR-22-3p-RT	CAGTGCAGGGTCCGAGGTCAGAGCCACCTGGGCAATTTTTTTTT TACAGTT
hsa-miR-22-3p-F	CACCAAGCTGCCAGTTGAAG
hsa-miR-125b-5p-RT	CAGTGCAGGGTCCGAGGTCAGAGCCACCTGGGCAATTTTTTTTT TTCACAA
hsa-miR-125b-5p-F	ACACCTCCCTGAGACCCTAAC
hsa-miR-378a-3p-RT	CAGTGCAGGGTCCGAGGTCAGAGCCACCTGGGCAATTTTTTTTT TGCCTTC
hsa-miR-378a-3p -F	CACCACTGGACTTGGAGTCA
hsa-miR-486-5p-RT	CAGTGCAGGGTCCGAGGTCAGAGCCACCTGGGCAATTTTTTTTT TCTCGGG
hsa-miR-486-5p-F	CTCCTCCTGTACTGAGCTGC

2. Primer sequence of SYBR qRT-PCR

Gene	Species	Primer sequence 5'→3'
MYO1C	Human	F：TGGAAATGCCAAGACCCTCC<br>R：TTCCGCTCCCCATGATTCTG

NPHS2	Human	F：AAGTCACAATGCACCATCC<br>R：GACCAAAGCTGTTTCCCA
SYNPO	Human	qH03A154921（Sangon Biotech, Shanghai）
TNF-α	Human	F：AGCAAGGACAGCAGAGGA<br>R：GGGGAGAGAGGGTGGAG
GAPDH	Human	B661104-0001 （Sangon Biotech, Shanghai）
Myo1c	Mouse	F：TATGGAACGCTACCGTGGTG<br>R：CAGTCTCTTGGTGGCCTCTG
Tnf-α	Mouse	F：AGACAGAGGCAACCTGACCAC<br>R：GCACCACCATCAAGGACTCAA
Nphs2	Mouse	F：GATGCGGAGTACGAGTGCC<br>R：GGGGAACTAGGACGGAGAGG
Gapdh	Mouse	B661304-0001（Sangon Biotech, Shanghai）

3. The sequence of siRNAs for MYO1C knockdown

Name	Sense strand	Antisense strand
MYO1C-si-1	CCCAUUAUGAGCCAGUGCU<br>UUTT	AAAGCACUGGCUCAUAAUG<br>GGTT
MYO1C-si-2	GCAGAGGAUUGAUUACGCC<br>AATT	UUGGCGUAAUCAAUCCUCU<br>GCTT
MYO1C-si-3	UGUAGCUCAAAGAAUCCCA<br>UUTT	AAUGGGAUUCUUUGAGCUA<br>CATT
GAPDH	UAAAGUACCCUGUGCUCAA<br>TT	UUGAGCACAGGGUACUUUA<br>TT
siRNA NC	UUCUCCGAACGUGUCACGU<br>TT	ACGUGACACGUUCGGAGAA<br>TT
CY3 siRNA NC	UUCUCCGAACGUGUCACGU<br>TT	ACGUGACACGUUCGGAGAA<br>TT

4. The sequence for MYO1C overexpression adenovirus and the target vector map were as follows.

Sequence:

CCAACTGCACCTCGGTTCTAAGCTTCTGCAGGTCGACCGCCACCATGGCGCTGCAAGT
GGAGCTGGTACCCACCGGGGAGATCATCCGCGTGGTTCATCCCCACAGGCCCTGCAAG
CTTGCCCTGGGCAGTGACGGGGTTCGGGTGACCATGGAGAGTGCGCTCACCGCCCGT
GACCGGGTGGGGGTGCAGGATTTCGTGCTGCTGGAGAACTTCACCAGCGAGGCCGCC
TTCATCGAGAACCTGCGGCGGCGATTTCGGGAGAATCTCATCTACACCTACATTGGCCC
CGTCCTGGTCTCTGTCAATCCCTACCGGGACCTGCAGATCTACAGCCGGCAGCATATGG
AGCGTTACCGTGGCGTCAGCTTCTATGAAGTGCCCCCTCACCTGTTTGCCGTGGCGGA
CACTGTGTACCGAGCACTGCGCACGGAGCGTCGGGACCAGGCTGTGATGATCTCTGGG
GAGAGCGGGGCAGGCAAGACCGAGGCCACCAAGAGGCTGCTGCAGTTCTATGCAGAG
ACCTGCCCAGCCCCCGAGCGCGGAGGTGCCGTGCGGGACCGGCTGCTACAGAGCAAC

CCGGTGCTGGAGGCCTTTGGAAATGCCAAGACCCTCCGGAACGATAACTCCAGCAGGT
TCGGGAAGTACATGGATGTGCAGTTTGACTTCAAGGGTGCCCCCGTGGGTGGCCACAT
CCTCAGTTACCTCCTGGAAAAGTCACGAGTGGTGCACCAGAATCATGGGGAGCGGAA
CTTCCACATCTTCTACCAGCTGCTGGAGGGGGGCGAGGAGGAGACTCTTCGCAGGCTG
GGCTTGGAACGGAACCCCCAGAGCTACCTGTACCTGGTGAAGGGCCAGTGTGCCAAA
GTCTCCTCCATCAACGACAAGAGTGACTGGAAGGTCGTCAGGAAGGCTCTGACAGTC
ATTGATTTCACCGAGGATGAAGTGGAGGACCTGCTGAGCATCGTGGCCAGCGTCCTTC
ATTTGGGCAACATCCACTTTGCTGCCAACGAGGAGAGCAATGCCCAGGTCACCACCGA
GAACCAGCTCAAGTATCTGACCAGGCTCCTCAGCGTGGAAGGCTCGACGCTGCGAGA
AGCCCTGACACACAGGAAGATCATCGCCAAGGGGGAGGAGCTCCTGAGCCCGCTGAA
CCTGGAGCAGGCCGCGTACGCACGAGACGCCCTCGCCAAGGCTGTGTACAGCCGCAC
TTTTACCTGGCTCGTCGGGAAGATCAACAGGTCGCTGGCCTCCAAGGACGTGGAGAGC
CCCAGCTGGCGGAGCACCACGGTTCTCGGGCTCCTGGATATTTATGGCTTTGAAGTGTT
TCAGCATAACAGCTTTGAGCAGTTCTGCATCAATTACTGCAACGAGAAGCTGCAGCAG
CTCTTCATCGAGCTCACGCTCAAGTCGGAGCAGGAGGAGTACGAGGCAGAGGGCATC
GCGTGGGAGCCCGTCCAGTATTTCAACAACAAAATCATCTGTGATCTGGTGGAGGAGA
AGTTTAAGGGCATCATCTCGATTTTGGATGAGGAGTGTCTGCGCCCCGGGGAGGCCAC
AGACCTGACCTTCCTGGAGAAGCTGGAGGATACTGTCAAGCACCATCCACACTTCCTG
ACGCACAAGCTGGCTGACCAGCGGACCAGGAAATCTCTGGGCCGAGGGGAATTCCGC
CTTCTGCACTATGCGGGGGAGGTGACCTACAGCGTGACCGGGTTTCTGGACAAAAACA
ATGACCTTCTCTTCCGGAACCTTAAGGAGACCATGTGTAGCTCAAAGAATCCCATTATG
AGCCAGTGCTTTGACCGGAGCGAGCTCAGTGACAAGAAGCGGCCAGAGACGGTCGCC
ACCCAGTTCAAGATGAGCCTCCTGCAGCTGGTGGAGATCCTGCAGTCTAAGGAGCCCG
CCTACGTCCGCTGCATCAAACCCAATGATGCCAAACAGCCCGGCCGCTTTGACGAGGT
GCTGATCCGCCACCAGGTGAAGTACCTGGGGCTGTTGGAAAACCTGCGCGTGCGCAG
AGCCGGCTTTGCCTATCGCCGCAAATACGAAGCTTTCCTGCAAAGGTACAAGTCACTG
TGCCCAGAGACGTGGCCCACGTGGGCAGGACGGCCGCAGGATGGGGTGGCTGTGCTG
GTCCGACACCTGGGCTACAAGCCAGAAGAGTACAAGATGGGCAGGACCAAGATCTTC
ATCCGCTTCCCCAAGACCCTGTTTGCCACAGAGGATGCCCTGGAGGTCCGGCGGCAGA
GCCTGGCCACAAAGATCCAAGCTGCCTGGAGGGGCTTTCACTGGCGGCAGAAATTCCT
CCGGGTGAAGAGATCAGCCATCTGCATCCAGTCGTGGTGGCGTGGAACACTGGGCCG
GAGGAAGGCAGCCAAGAGGAAGTGGGCGGCACAGACCATCCGGCGGCTCATCCGAG
GCTTCGTCCTGCGCCACGCCCCCCGCTGCCCCGAGAACGCCTTCTTCCTGGACCATGT
GCGCACCTCTTTTTTGCTAAACCTGAGGCGGCAGCTGCCCCAGAATGTCCTGGACACC
TCGTGGCCCACGCCCCCACCTGCCCTGCGTGAGGCCTCAGAGCTTCTGCGGGAGTTGT
GCATAAAGAACATGGTGTGGAAATACTGCCGGAGTATCAGCCCTGAGTGGAAGCAGCA
GCTGCAGCAGAAGGCCGTGGCTAGTGAGATCTTCAAGGGCAAGAAGGATAATTACCCT
CAGAGTGTACCCAGGCTCTTCATCAGCACTCGGCTTGGTACAGATGAGATCAGCCCCC
GAGTGCTGCAGGCCTTGGGCTCTGAGCCCATTCAGTATGCGGTGCCTGTTGTGAAATA
CGACCGCAAGGGCTACAAGCCTCGCTCCCGGCAGCTGCTGCTCACGCCCAACGCCGT
CGTCATCGTGGAGGACGCCAAAGTCAAGCAGAGGATTGATTACGCCAACCTGACCGG
AATCTCTGTCAGCAGCCTGAGCGACAGTCTTTTTGTGCTTCATGTACAGCGTGCGGAC
AATAAGCAAAAGGGAGATGTGGTGCTGCAGAGTGACCACGTGATTGAGACGCTGACC
AAGACAGCCCTCAGTGCCAACCGCGTGAACAGCATCAACATCAACCAGGGCAGCATC

ACGTTTGCAGGGGGCCCCGGCAGGGATGGCACCATTGACTTCACACCCGGCTCGGAG
CTGCTCATCACCAAGGCCAAGAACGGGCACCTGGCTGTGGTCGCCCCACGGCTGAATT
CTCGGGGATCCATGGACTACAAGGATGACGATGACAAGGATTACAAAGACGACGATGA
TAAGGACTATAAGGATGATGACGACAAATCTAGATGACTCGAGTCCATCGATACTAGTA
AGGATCTGCGATCGCTCCGGTGCCCGTCAGTGGGCAGAGCGCACATCGCCCACAGTCC
CCGAGAAGTTGGGGGGAGGGGTCG

Vector Map:

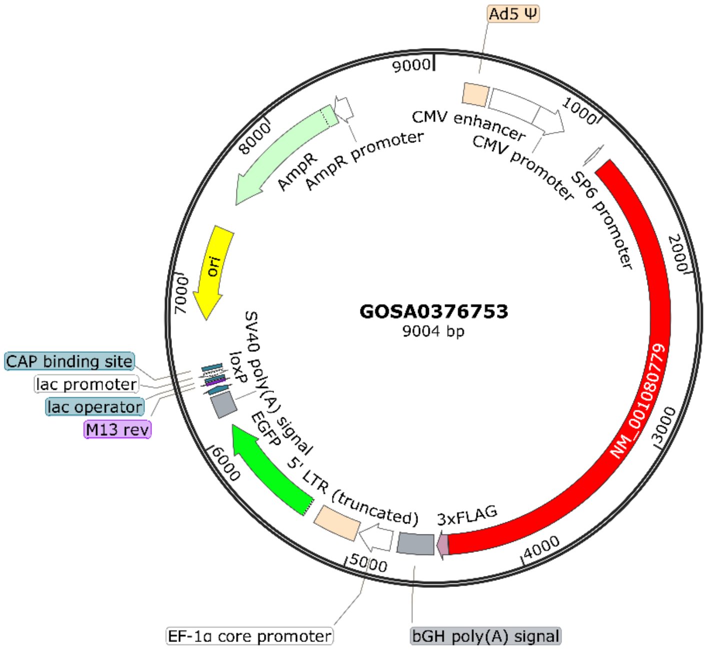

The transfection of MYO1C overexpressing adenovirus was observed by immunofluorescence assay.

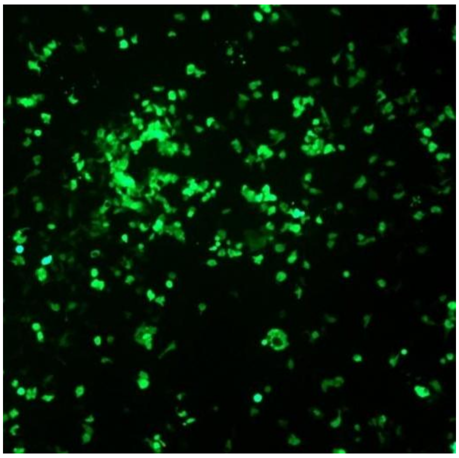

5. The sequence for MYO1C knockdown adeno-associated viral and the target vector map were as follows.

mir30-m-1-MYO1C:GACAACAAGCAGAAGGGAGAT

Vector Map: pHBAAV-nphs2-EGFP vector

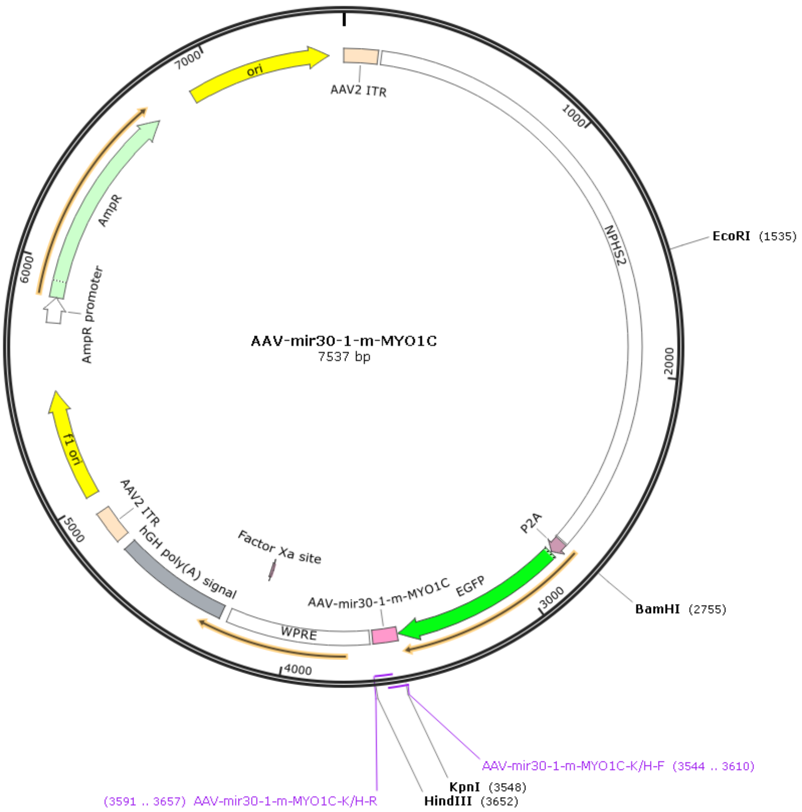
AAV-mir30-1-m-MYO1C
7537 bp
AAV2 ITR
NPHS2
EcoRI (1535)
P2A
BamHI (2755)
EGFP
AAV-mir30-1-m-MYO1C
WPRE
Factor Xa site
hGH poly(A) signal
AAV2 ITR
f1 ori
AmpR promoter
AmpR
ori
AAV-mir30-1-m-MYO1C-K/H-F (3544 .. 3610)
KpnI (3548)
HindIII (3652)
(3591 .. 3657) AAV-mir30-1-m-MYO1C-K/H-R
1000
2000
3000
4000
5000
6000
7000

Supplemental Tables and Figures

Supplemental Table S1. Summary of differential expression analysis for the six candidate RNA biomarkers

Gene Symbol	log2(Fold Change)	Adjusted p-value (FDR)	KEGG pathways*	Rank**
MYO1C mRNA	3.045833333	0.032319732	Apoptosis	2
SP100 mRNA	4.093333333	0.006727558	p53 signaling pathway	1
hsa-miR-21-5p	1.085192161	0.000856025	/	3
hsa-miR-22-3p	1.144319097	0.004849052	/	2
hsa-miR-378a-3p	1.06745446	0.013839294	/	4
hsa-miR-486-5p	2.163360096	0.003829015	/	1

* KEGG pathways related to diabetes and kidney disease in the ceRNA network

** Fold Change Rank in ceRNA network

Supplemental Table S2. Training and validation cohort main clinical characteristics of patients in both groups

	T_2DM (n=33)	T_2DN (n=40)	*p*
Age, years	57.52±10.45	53.23±8.53	0.057
Sex, male (%)	20, 60.6%	25, 62.5%	1.000
Diabetes duration, years	6.00 [2.00, 14.00]	10.50 [8.00, 17.25]	0.006
Body mass index, kg/m^2	26.06±3.13	24.71±3.19	0.073
Systolic blood pressure, mm Hg	122.91±11.08	136.93±20.03	0.001
Diastolic blood pressure, mm Hg	77.27±6.73	82.15±9.91	0.019
Fasting glucose, mg/dL	7.15±2.26	5.84±2.35	0.018
Glycohemoglobin, %	8.66±1.94	6.61±1.06	<0.001
Hemoglobin, g/dL	134.91±15.96	99.92±17.76	<0.001
Serum Albumin, g/dL	44.01±3.75	33.72±6.86	<0.001
ALT， U/L	22.67±13.15	16.12±11.61	0.027
AST， U/L	22.06±18.80	20.15±15.02	0.631
Urea nitrogen, mg/dL	19.10±2.91	44.69±23.1	<0.001
Serum creatinine, mg/dL	0.84±0.29	3.18±2.81	<0.001
Uric acid, mg/dL	336.39±76.01	379.28±94.15	0.038
eGFR， mL/min*1.73m^2	91.74±21.96	33.92±26.95	<0.001
Total cholesterol, mg/dL	4.29 [3.63, 4.99]	4.56 [3.69, 5.44]	0.280
Triglycerides, mg/dL	1.98±1.28	2.26±2.03	0.479
HDL, mg/dL	1.13±0.36	1.11±0.35	0.812
LDL, mg/dL	2.24 [1.84, 2.85]	2.66 [1.92, 3.04]	0.258
24h urine volume, L	2.20±0.57	2.15±0.82	0.798
24h urine total protein, g	0.26 [0.24, 0.33]	4.88 [3.04, 7.02]	<0.001
UACR, mg/g	43.02±70.41	3882.41±2497.91	<0.001

The data were expressed as mean ± SD or quartile. The t test, Mann-Whitney U test and chi-square test were used to compare the data between the two groups. The p value <0.05 had statistical difference. Abbreviations: ALT: alanine transaminase; AST: aspartate transaminase; eGFR: estimated glomerular filtration rate; h: hour; UACR: urine albumin creatinine ratio.

Supplemental Table S3. Detailed performance evaluation of six machine learning models and ensemble models in the training cohort.

Model_names	AUC	Accuracy	Precision	Recall	F1 score
Random forest	0.846 (0.783, 0.914)	0.767 (0.743, 0.793)	0.800 (0.644, 0.956)	0.834 (0.712, 0.957)	0.786 (0.750, 0.823)
SVM	0.818 (0.503, 0.846)	0.507 (0.408, 0.607)	0.530 (0.419, 0.683)	0.875 (0.750, 1.000)	0.66 (0.563, 0.743)
BPNN	0.772 (0.705, 0.844)	0.659 (0.611, 0.702)	0.694 (0.573, 0.83)	0.743 (0.572, 0.824)	0.695 (0.607, 0.74)
XGBoost	0.872 (0.808, 0.943)	0.767 (0.708, 0.827)	0.775 (0.600, 0.950)	0.867 (0.769, 0.967)	0.791 (0.717, 0.865)
CatBoost	0.870 (0.804, 0.941)	0.714 (0.663, 0.765)	0.748 (0.596, 0.900)	0.816 (0.678, 0.954)	0.748 (0.705, 0.791)
LightGBM	0.818 (0.751, 0.888)	0.712 (0.654, 0.771)	0.696 (0.570, 0.822)	0.828 (0.746, 0.909)	0.745 (0.665, 0.825)
**VotingClassifier**	**0.877 (0.797, 0.958)**	**0.741 (0.702, 0.779)**	**0.784 (0.625, 0.944)**	**0.816 (0.678, 0.954)**	**0.766 (0.735, 0.796)**

Supplemental Table S4. Test cohort main clinical characteristics of patients in both groups

	T_2DM (n=20)	T_2DN (n=17)	*p*
Age, y	52.85±13.49	59.59±11.65	0.116
Gender, male (%)	11, 55%	10, 58.8%	1.000
Diabetes duration, y	9.50 [7.75, 11.00]	10.00 [10.00, 11.00]	0.259
Body mass index, kg/m^2	22.77±3.52	21.82±1.75	0.320
Systolic BP, mm Hg	141.05±25.94	142.59±16.67	0.835
Diastolic BP, mm Hg	82.1±12.14	84.59±9.37	0.496
Fasting glucose, mg/dL	9.55±4.24	6.99±2.37	0.034
Glycated hemoglobin, %	8.85±3.56	7.19±1.66	0.086
Hemoglobin, g/dL	124.85±20.6	102.71±24.69	0.005
Serum albumin, g/dL	39.34±4.98	35.12±7.2	0.043
ALT, U/L	20.75±8.04	27.24±12.12	0.060
AST, U/L	26.47±11.50	28.25±10.95	0.633
BUN, mg/dL	11.70±4.41	23.88±17.23	0.004
Serum creatinine, mg/dL	0.58±0.17	1.63±1.54	0.004
Uric acid, μmol/L	237±115.28	277.82±79.94	0.227
eGFR, mL/min*1.73m^2	114.1±14.5	57.1±38.11	<0.001
Total cholesterol, mmol/L	4.41 [3.55, 5.13]	4.61 [3.51, 5.02]	0.831
Triglycerides, mmol/L	2.12±2.63	2.18±2.91	0.948
HDL, mmol/L	1.09±0.32	1.12±0.37	0.738
LDL, mmol/L	2.16 [1.59, 2.88]	2.64 [1.86, 2.94]	0.681
24h urine volume, L	2.58±0.55	2.32±0.73	0.230
24h urine total protein, g	0.02 [0.01, 0.06]	4.10 [2.90, 8.00]	<0.001
UACR, mg/g	27.81±61.26	3222.98±1473.33	<0.001

The data were expressed as mean ± SD or quartile. The t test, Mann-Whitney U test and chi-square test were used to compare the data between the two groups. The p value <0.05 had statistical difference. Abbreviations: ALT: alanine transaminase; AST: aspartate transaminase; eGFR: estimated glomerular filtration rate; h: hour; UACR: urine albumin creatinine ratio.

Supplemental Table 5. Detailed performance evaluation of six machine learning models and ensemble models in the test cohort.

Model_names	AUC	Accuracy	Precision	Recall	F1 score
Random forest	0.813 (0.643, 0.942)	0.703 (0.541, 0.839)	0.871 (0.599, 0.954)	0.407 (0.167, 0.667)	0.548 (0.300, 0.765)
SVM	0.812 (0.661, 0.947)	0.463 (0.297, 0.622)	0.457 (0.297, 0.622)	1.000 (1.000, 1.000)	0.627 (0.458, 0.767)
BPNN	0.804 (0.556, 0.899)	0.728 (0.568, 0.865)	0.821 (0.556, 0.962)	0.535 (0.278, 0.778)	0.636 (0.400, 0.828)
XGBoost	0.797 (0.616, 0.938)	0.697 (0.541, 0.838)	0.804 (0.500, 0.907)	0.471 (0.231, 0.714)	0.586 (0.348, 0.774)
CatBoost	0.809 (0.655, 0.941)	0.759 (0.622, 0.892)	0.899 (0.667, 1.000)	0.535 (0.278, 0.765)	0.658 (0.421, 0.839)
LightGBM	0.747 (0.520, 0.867)	0.756 (0.622, 0.892)	0.832 (0.571, 0.887)	0.584 (0.333, 0.824)	0.682 (0.462, 0.864)
**VotingClassifier**	**0.824 (0.673, 0.952)**	**0.725 (0.568, 0.865)**	**0.820 (0.556, 1.000)**	**0.522 (0.278, 0.765)**	**0.638 (0.417, 0.828)**

Supplemental Figure 1. (A) RT-qPCR validation of candidate RNA expression levels in uEVs from T_2DN and type 2 diabetes patients in the training cohort. Statistical comparisons between groups were performed using the Mann-Whitney *U* test, with p-values indicated on the bar graphs. n=73. **(B)** ROC curve analysis for RNA expression distinguishing T_2DN from type 2 diabetes. AUC: Area Under the ROC Curve. **(C)** Heatmap depicting the correlation between expression levels of candidate RNAs and clinical features. **(D)** Correlation analysis of the five features included in the classification model. Diagonal subplots display distribution curves for individual features, while off-diagonal subplots show scatterplots of correlation between feature pairs with fitted regression lines. The control group represents type 2 diabetes patients, and the disease group represents T_2DN patients.

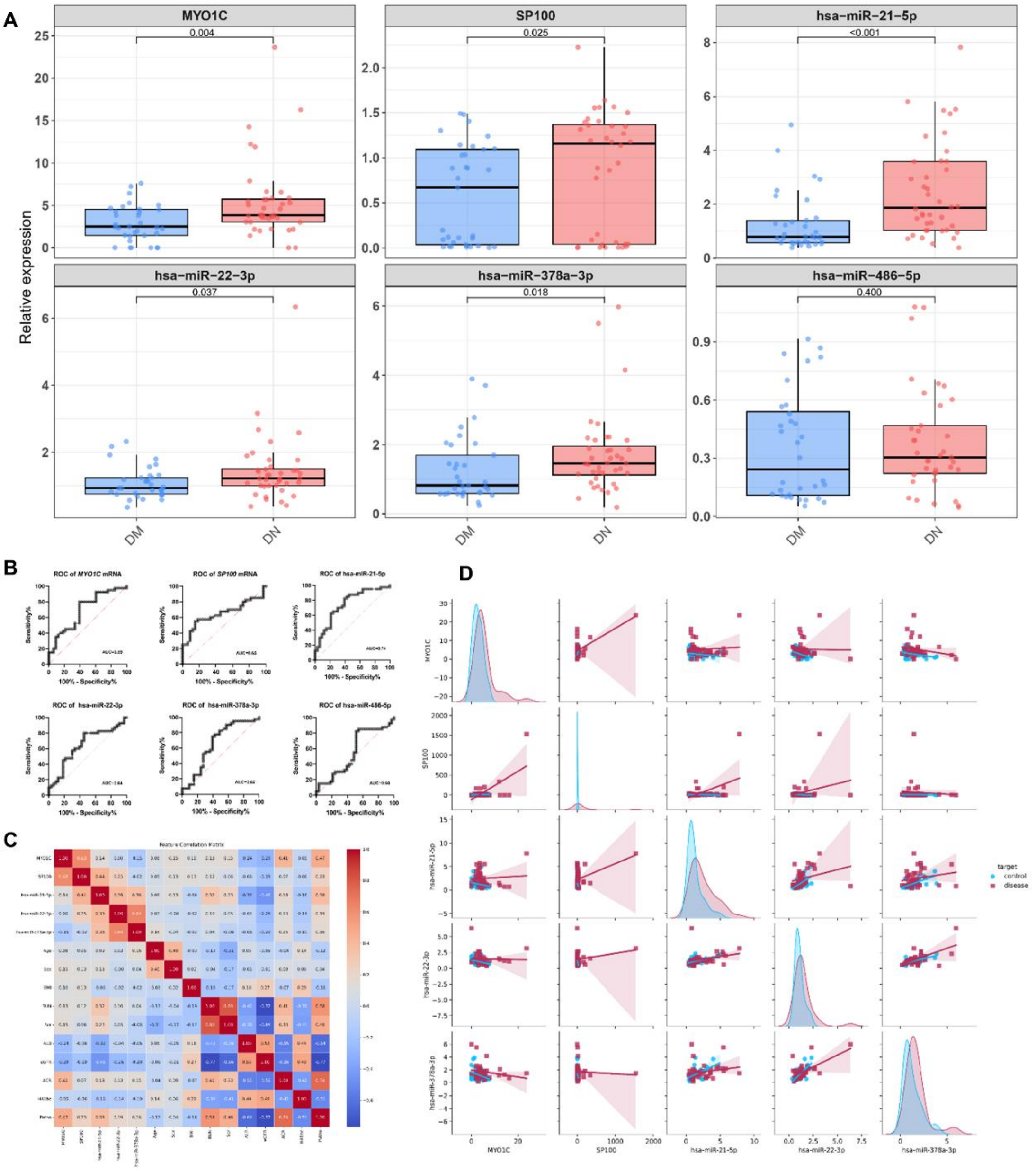

Supplemental Figure 2. RF model hyperparameter adjustment and model evaluation visualization

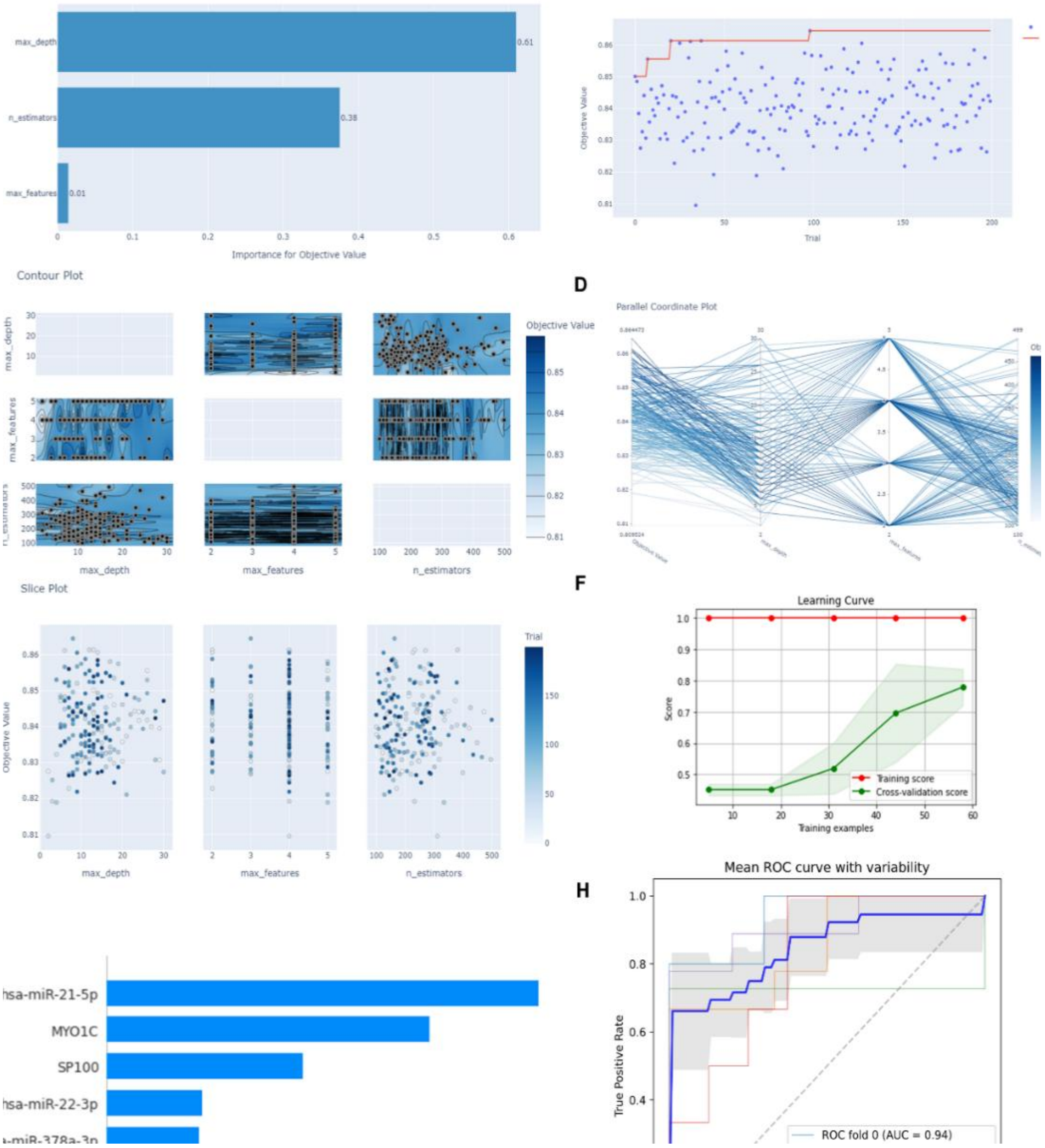

Supplemental Figure 3. SVM model hyperparameter adjustment and model evaluation visualization

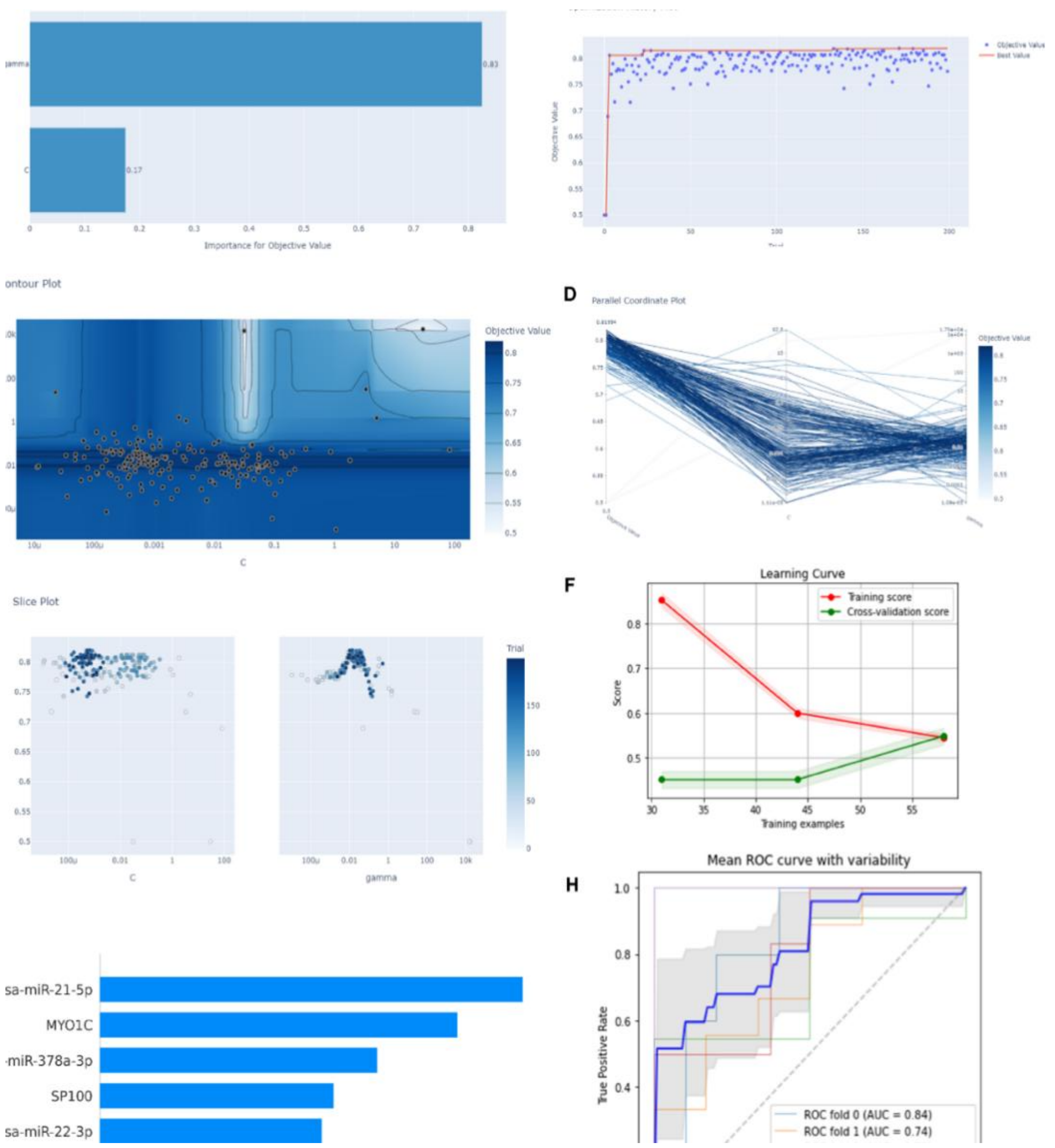

Supplemental Figure 4. BPNN model hyperparameter adjustment and model evaluation visualization

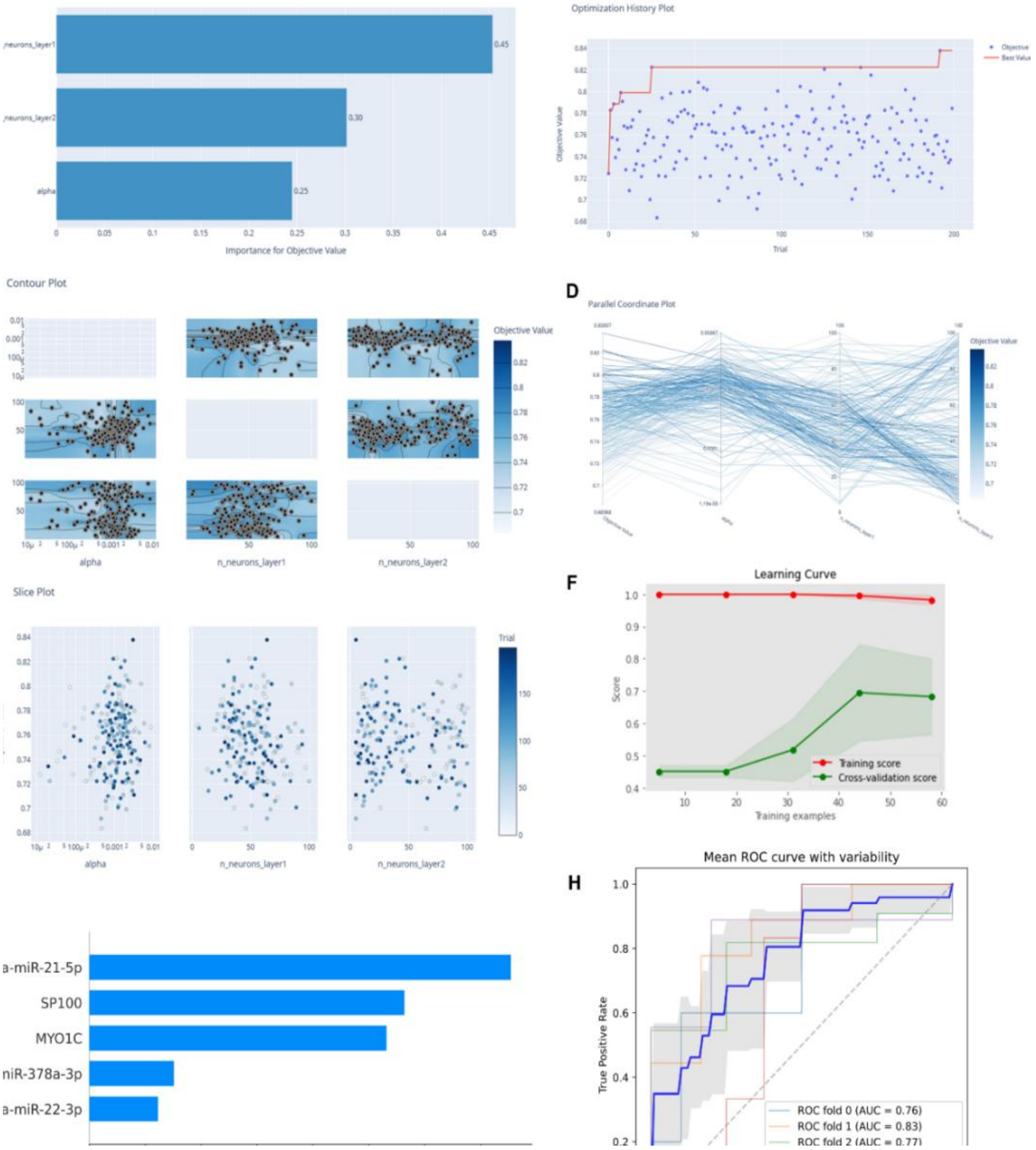

Supplemental Figure 5. XGBoost model hyperparameter adjustment and model evaluation visualization

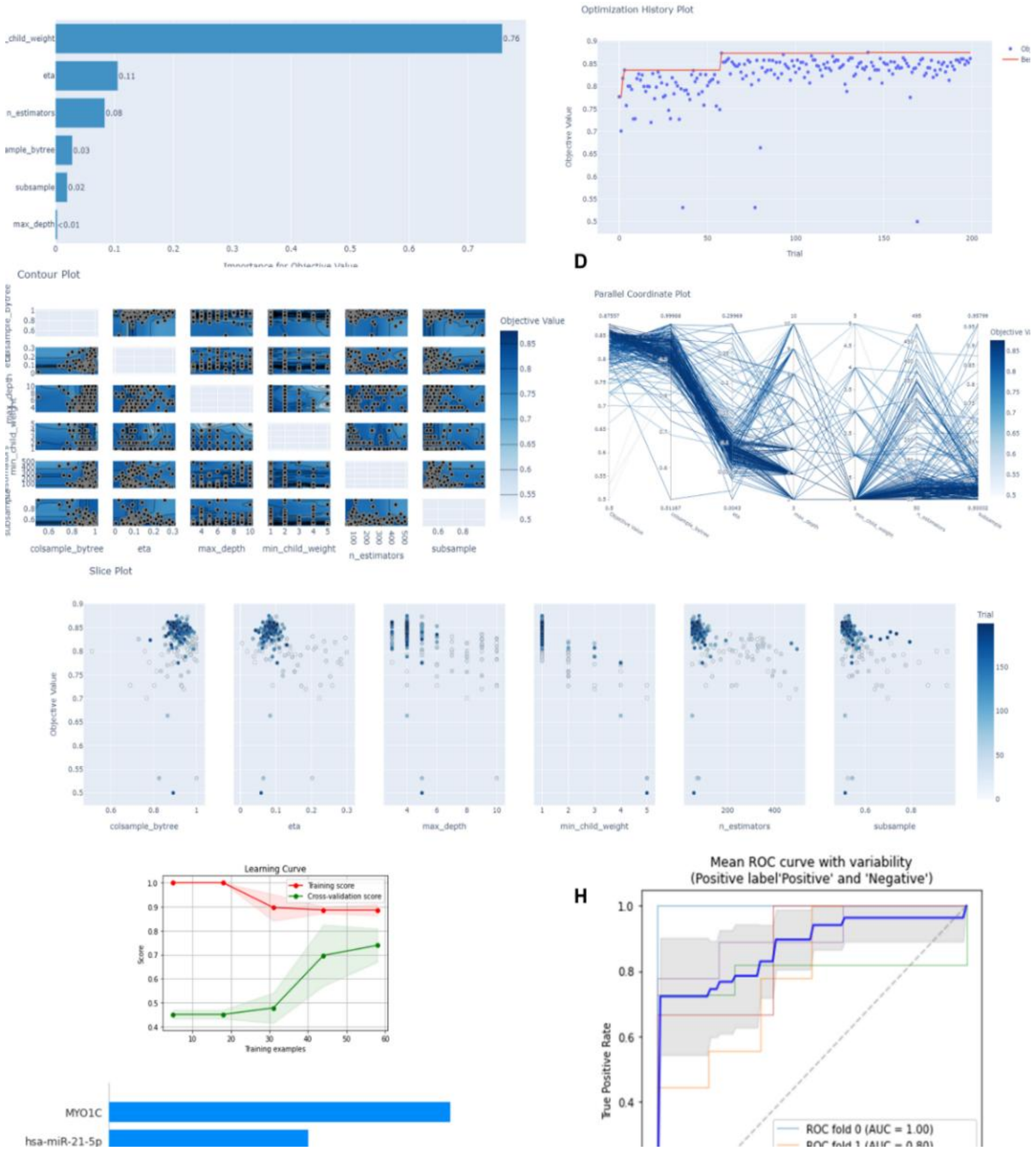

Supplemental Figure 6. LightGBM model hyperparameter adjustment and model evaluation visualization

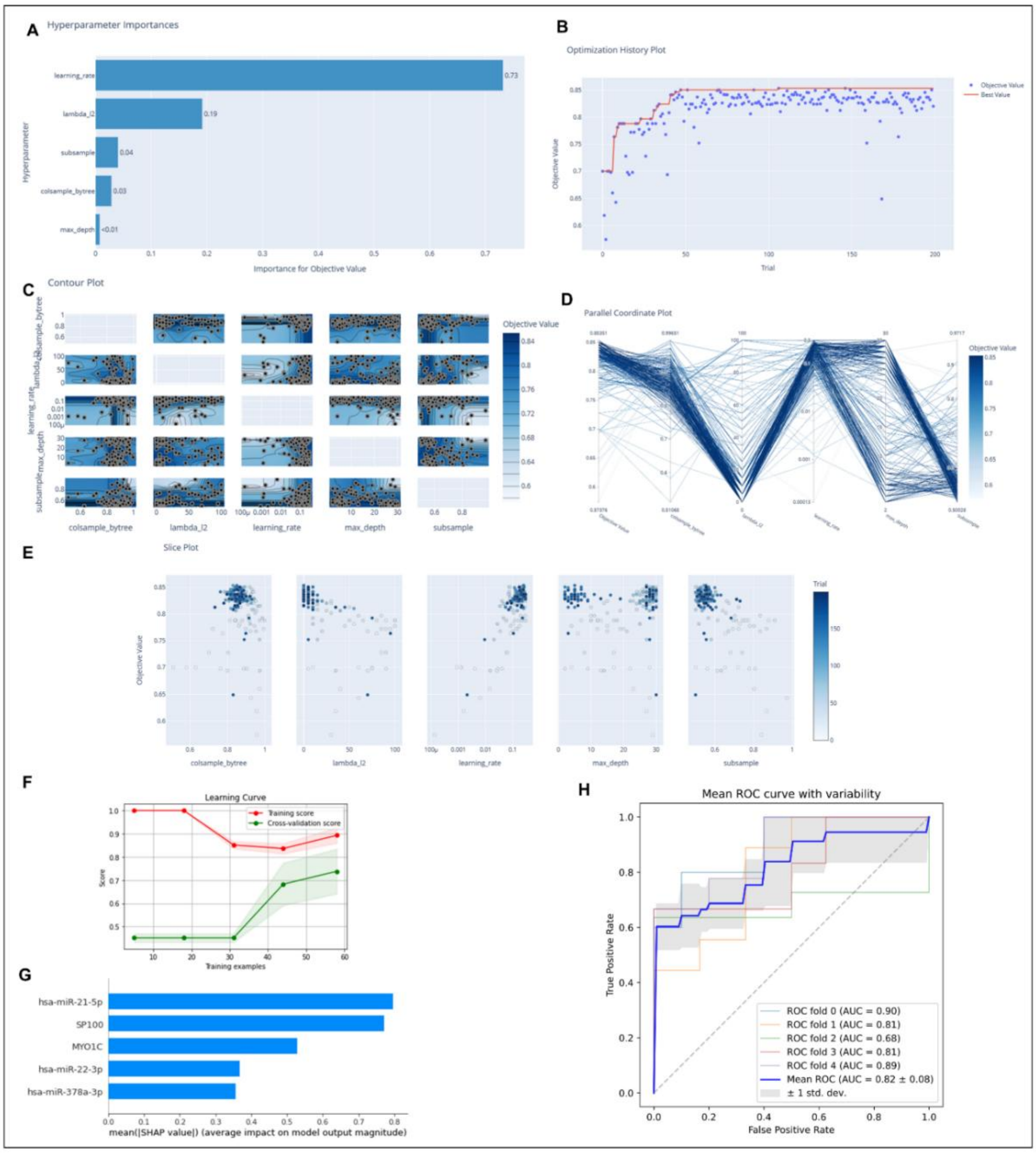

Supplemental Figure 7. CatBoost model hyperparameter adjustment and model evaluation visualization

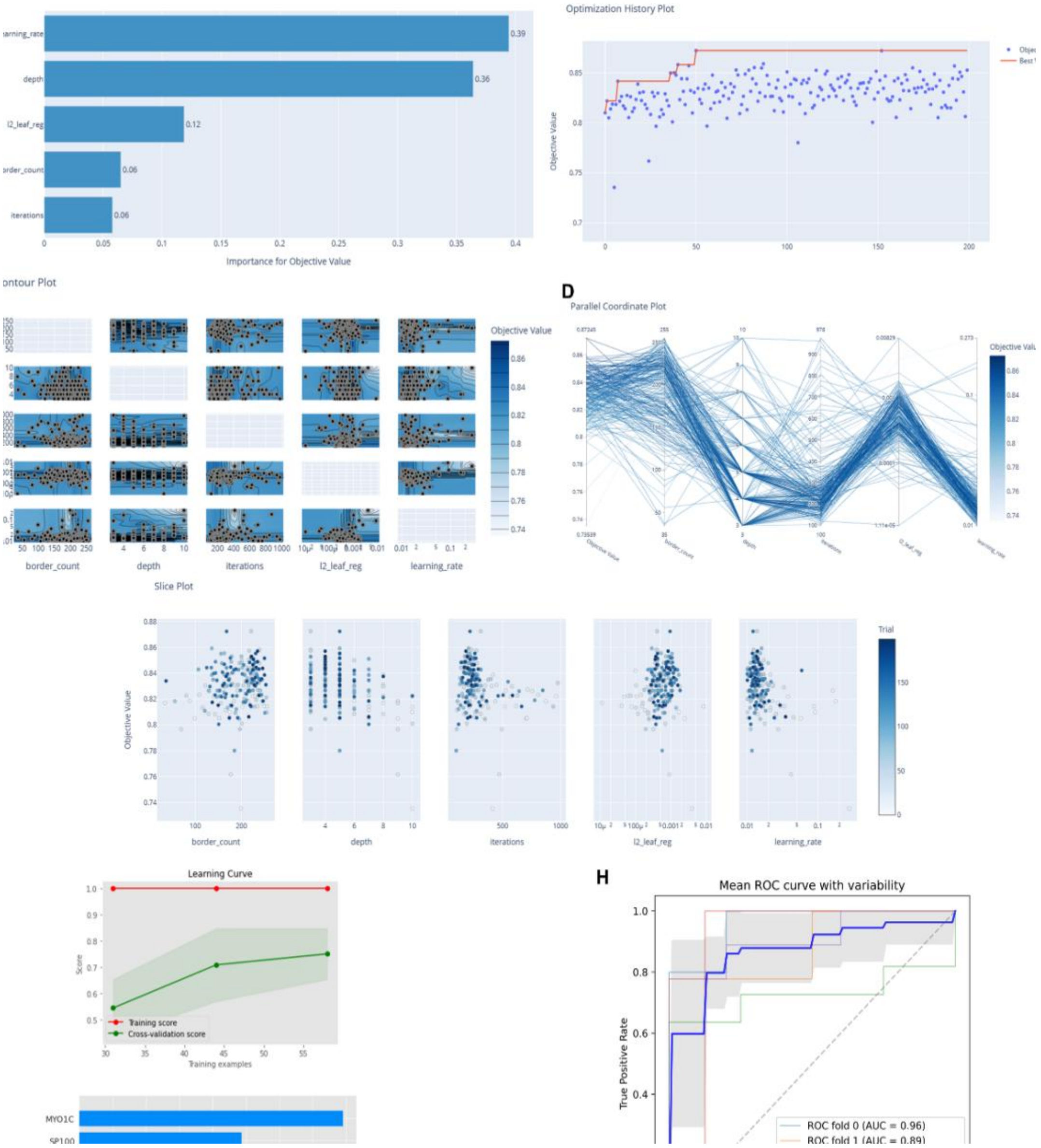

Supplemental Figure 8. (A) Heatmap of relative expression of *MYO1C* mRNA, *SP100* mRNA, hsa-miR-21-5p, has-miR-22-3p, and has-miR-378a-3p in test cohort. Data were normalized using Z-scores for visualization; **(B, C)** Correlation scatterplots depicting *MYO1C* mRNA expression in relation to UACR (orange) and eGFR (blue) after combining the training and test cohort.

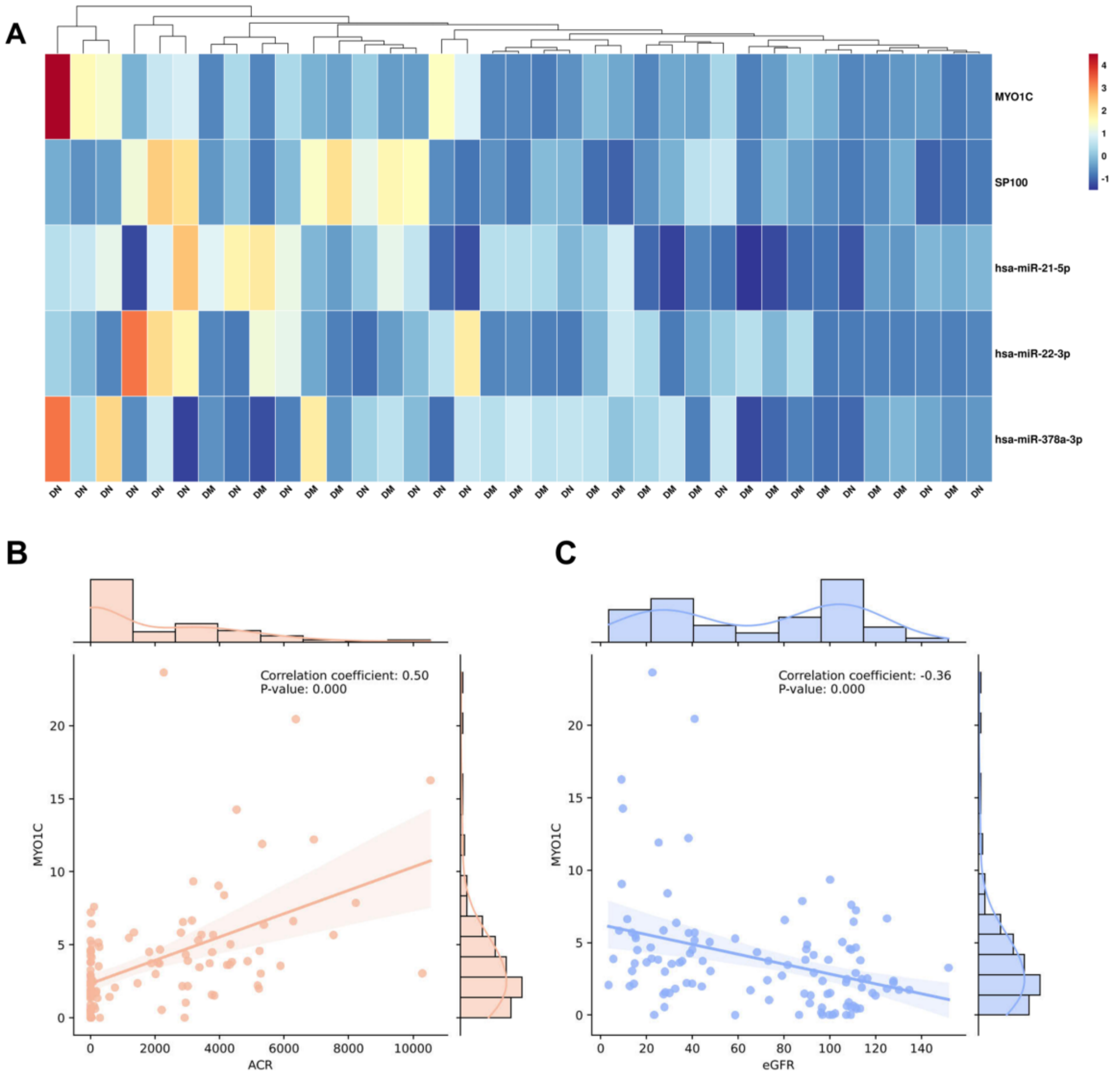

Supplemental Figure 9. (A) Common light microscope bright field diagram of three groups of HPCs, bar=50 μm. NG: normal culture group, HM: hypertonic control group, HGHF: high glucose and high fat group. **(B)** *MYO1C* mRNA expression in HPCs transfected with siRNA, measured by RT-qPCR. Data are presented as mean ± SEM, with statistical comparisons performed using one-way ANOVA with Dunnett's multiple comparison test. **(C)** Immunoblots of MYO1C protein levels in HPCs transfected with siRNA. Data represent four independent experiments.

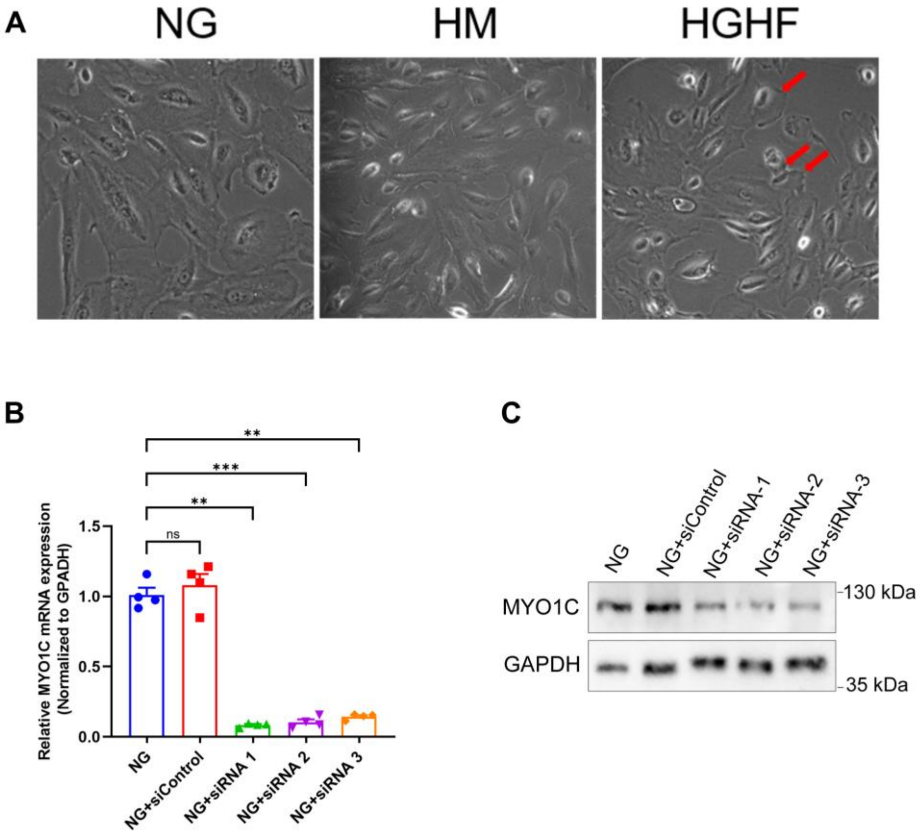

Supplemental Figure 10. ELISA detection of inflammatory factors in kidney tissue homogenate of mice in each group. The expression levels of MCP-1, TNF-α, IL-1β, IL-6 and IL-18 in tissue homogenate of mice were detected by ELISA. Data were expressed as mean ± standard error and comparisons between groups were made using Dunnett's one-way test of variance for multiple comparisons. n=10 in each group.

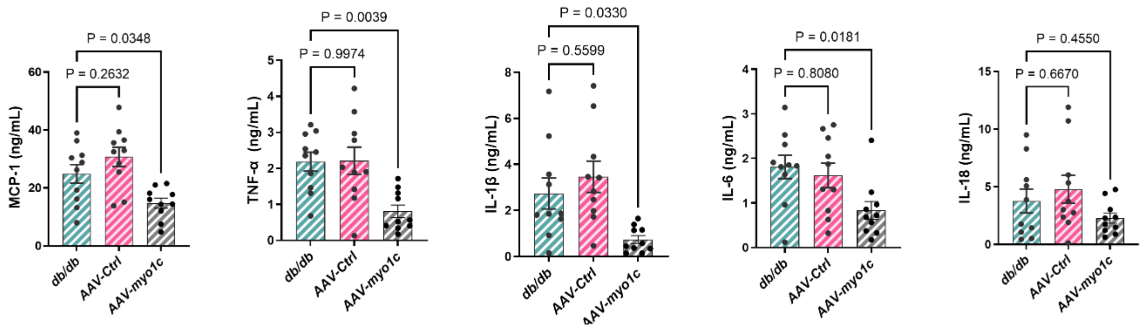

Supplemental Figure 11. Model of simulated protein-protein docking for MYO1C and p38. A. Summary table of Docking Score and Confidence_score for the top 10 models; B. 3-D protein structure binding prediction diagram of model_1 model with the best binding results; C-D. The protein binding process is shown in Surface form.

A

Summary of the Top 10 Models

Rank	1	2	3	4	5	6	7	8	9	10
Docking Score	-299.79	-277.14	-269.48	-262.81	-254.15	-253.34	-246.68	-245.39	-244.84	-236.03
Confidence Score	0.9524	0.9271	0.916	0.9052	0.8892	0.8876	0.8736	0.8708	0.8695	0.8482
Interface residues	model_1	model_2	model_3	model_4	model_5	model_6	model_7	model_8	model_9	model_10

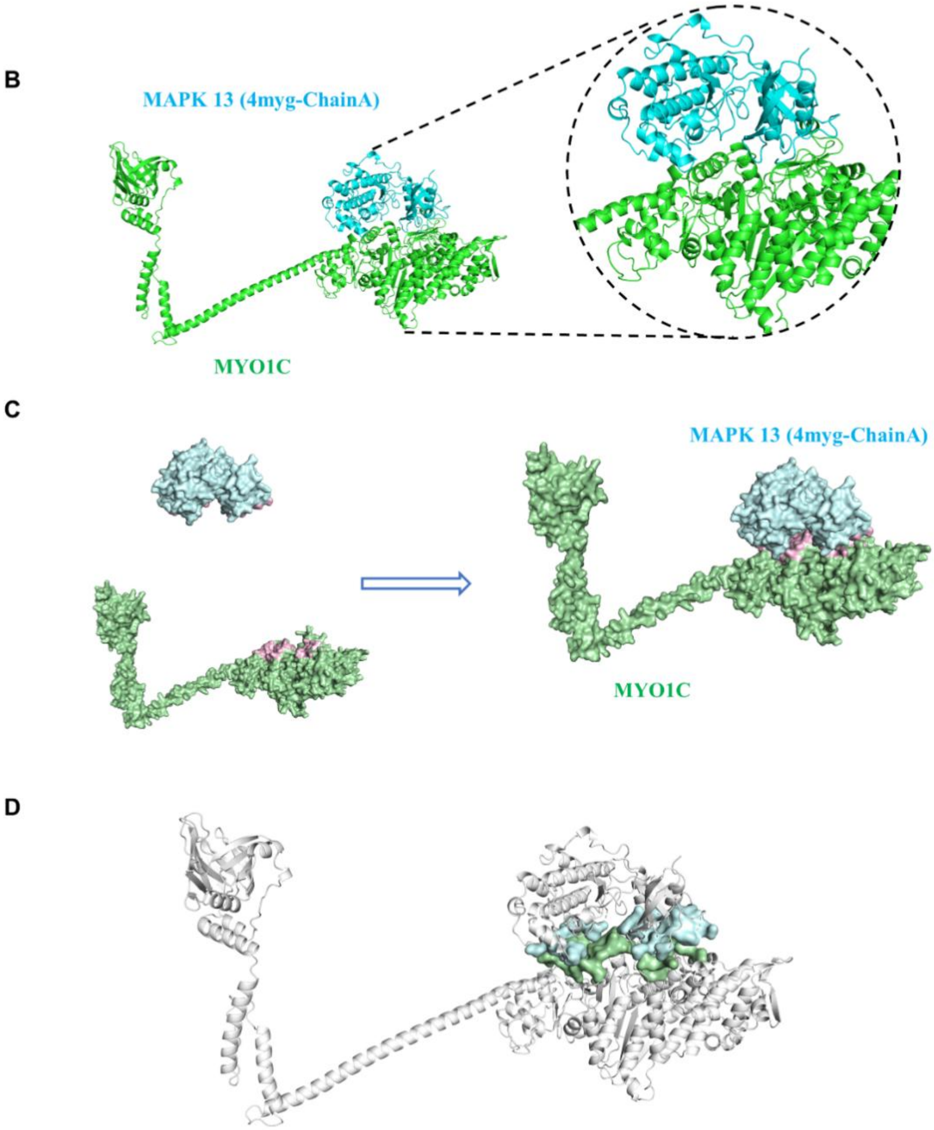

Supplemental Figure 12. MYO1C-p38 expression was detected by co-immunoprecipitation assay after 48h high glucose and high lipid stimulation of HPCs. **(A)** 2D interaction map of the simulated MYO1C-p38 protein-protein complex. **(B)** 3D interaction model of the MYO1C-p38 complex. MYO1C is located below the black dashed line, and p38 is above. Carbon atoms are shown in black, oxygen atoms in red, nitrogen atoms in blue, green dashed lines represent hydrogen bonds (with bond lengths labeled), red dashed lines indicate salt bridges, and semicircles represent hydrophobic interaction residues. **(C)** Immunoblots of specified proteins in HPCs transfected with MYO1C-overexpressing adenovirus (Ad-MYO1C) and controls. **(D)** Adenoviral transfection was used to overexpress MYO1C in HPCs, with fluorescence microscopy confirming appropriate transfection efficiency at the selected viral titer. **(E)** Immunoblots of specified proteins in HPCs transfected with MYO1C-overexpressing adenovirus (Ad-MYO1C) and controls.

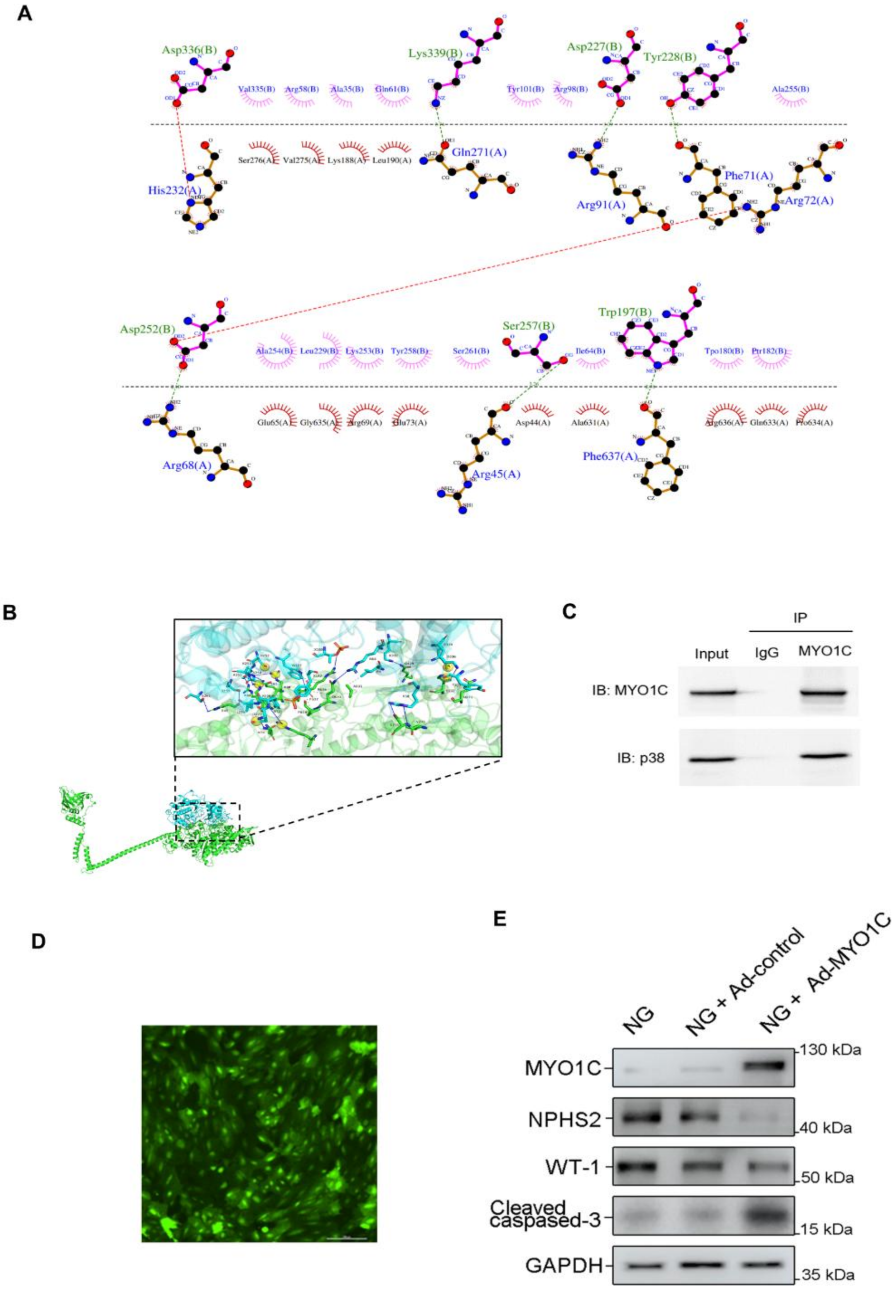

Supplemental Methods

Selection of candidate RNA biomarkers. Candidate selection followed a multi-step process: a) Statistical screening: mRNA data from the Gene Expression Omnibus (GSE218929) were used, requiring at least one probe marked "Detected" with a 100% detection rate. For miRNA, differential expression was analyzed using DESeq2 from raw reads. Transcripts with adjusted p-value (Benjamini-Hochberg FDR) < 0.05 and | log2 fold change | > 1.0 were considered significantly dysregulated. b) Interaction network and functional enrichment: ENCORI predicted lncRNA/circRNA-miRNA- mRNA interactions were refined using TargetScan and miRanda. Pearson correlation (r>0.9) was applied to construct the ceRNA network, and KEGG pathway enrichment (p < 0.05) identified disease-related pathways. c) Candidate prioritization: Based on the steps outlined in a) and b) from the previous study, candidates that were highly dysregulated and involved in relevant pathways were prioritized. Considering their potential for clinical biomarker development and supporting literature, the six RNA candidates selected for this study were: the four core miRNAs (hsa-miR-21-5p, hsa-miR-22-3p, hsa-miR-378a-3p, and hsa-miR-486-5p), highly expressed in the ceRNA network, and MYO1C and SP100 mRNA, the two mRNAs with the highest fold changes, associated with diabetes and kidney disease pathways in KEGG analysis (Supplemental Table 1).

Patient Cohorts, uEVs isolation and characterization. All type 2 diabetes patients met WHO diagnostic criteria, while T_2DN patients had biopsy-confirmed diabetic nephropathy with UACR >30 mg/g or 24-hour urinary microalbumin >30 mg/24 h, excluding other primary or secondary kidney diseases. Morning urine samples (100–200 mL) were collected and subjected to differential centrifugation, with a final ultracentrifugation step at 200,000 ×g for 2 hours at 4°C, repeated with PBS resuspension to obtain uEVs. Written informed consent was obtained from all participants before sample and data collection.

Transmission electron microscopy (TEM, HT7700, Hitachi, Tokyo, Japan) was used to observe uEV morphology by imaging copper grid-mounted, negatively stained EV samples at 100 or 80 kV. For fluorescent labeling and nano-flow cytometry, uEVs were incubated with fluorescently labeled anti-CD9 antibodies, followed by centrifugation and resuspension, and analyzed using NanoFCM (NanoFCM Inc, Xiamen, China).

Machine learning model development and feature analysis. Model development employed

Python's Scikit-learn library, implementing five-fold cross-validation and constructing six machine learning models: Random Forest (RF), Support Vector Machine (SVM), Back Propagation Neural Network (BPNN), Extreme Gradient Boosting (XGBoost), Light Gradient Boosting Machine (LightGBM), and CatBoost. Automated hyperparameter tuning for each model was conducted using Optuna, an open-source hyperparameter optimization framework. A voting ensemble classifier was created using the VotingClassifier module from Scikit-learn's ensemble library to integrate individual models. Model performance was evaluated using several key metrics [True positives (TP), True Negatives (TN), False positives (FP), False Negatives (FN)]: Area under the ROC curve (AUC): Measures overall model discrimination. Accuracy: The proportion of correctly classified instances in the dataset, calculated as Accuracy= $(TP+TN)/(FP+FN+TP+TN)$. Precision: The proportion of true positive predictions out of all positive predictions, calculated as Precision= $TP/(TP+FP)$. Recall: The proportion of actual positive cases correctly identified by the model, calculated as Recall= $TP/(TP+FN)$. F1 Score: The harmonic mean of Precision and Recall, calculated as F1= $2\times[(Precision\times Recall)/(Precision+Recall)]$. Feature importance and contributions to predictions were analyzed using SHapley Additive exPlanations (SHAP). SHAP values quantified each feature's contribution to model predictions, providing interpretability and insights into model behavior.

Cell culture. Before siRNA transfection, cells were serum-starved for 12 hours. Transfections were performed using Lipofectamine 3000 (L3000015, Thermofisher) at a final siRNA concentration of 75 nM, following the manufacturer's protocol. The medium was replaced with fresh culture medium 4-6 hours post-transfection. Cells were exposed to the following media: normal glucose (NG, 5.6 mM D-glucose) with 28.8 mM mannitol as an osmotic control (HM); or high-glucose (30 mM) medium supplemented with 50-600 μM PA. Cell viability was assessed by Cell Counting Kit-8 (CCK-8, Dojindo Laboratories, Japan) at 12, 24, and 48 h. For subsequent experiments, HPCs were maintained in NG, HM, or HGHF medium (30 mM glucose + 150 μM PA).

siRNA, adenovirus transfection and chemical modulation in HPCs. To achieve MYO1C overexpression, HPCs were infected with adenoviruses in three experimental groups: normal glucose control (NG), adenovirus-empty vector control (NG+Ad-Control), and MYO1C-overexpressing adenovirus (NG+Ad-MYO1C). Differentiated HPCs at 37°C were seeded into six-well plates at 60-70% confluence and infected with adenoviruses (4×10^6 cells; MOI=100) according

to the manufacturer's protocol. Fresh medium was added 6-8 hours post-infection. Control adenoviruses carried an EGFP tag, and the transfection efficiency was confirmed (as shown in Supplementary Method Materials).

For p38 inhibition, SB203580 (HY-10256, MedChemExpress) was used at a final concentration of 30 μM. HPCs were divided into four groups: normal glucose control (NG), adenovirus-empty vector control (NG+Ad-Control), MYO1C-overexpressing adenovirus (NG+Ad-MYO1C), and MYO1C-overexpressing adenovirus with SB203580 treatment (NG+Ad-MYO1C+SB203580).

For p38 activation, Dehydrocorydaline (HY-N0674, MedChemExpress) was used at a final concentration of 10 μM. HPCs were assigned to six groups: normal glucose control with siRNA control (NG+siCtrl), normal glucose with MYO1C knockdown siRNA (NG+siRNA), normal glucose with MYO1C knockdown siRNA and Dehydrocorydaline (NG+siRNA+p38 agonist), HGHF with siRNA control (HGHF+siCtrl), HGHF with MYO1C knockdown siRNA (HGHF+siRNA), and HGHF with MYO1C knockdown siRNA and Dehydrocorydaline (HGHF+siRNA+p38 agonist).

Western immunoblot analysis. For immunoblotting, proteins were separated using SDS-PAGE gels. A separating gel of 10% or 15% was chosen based on the molecular weight of the target protein, while a 4% stacking gel was used for all samples. After electrophoresis, proteins were transferred to a membrane using a wet transfer system. The transfer conditions, including constant current and transfer time, were adjusted according to the molecular weight of the target protein.

Mouse model and measurements. To knockdown MYO1C in *vivo*, an adeno-associated viral empty vector (AAV-Ctrl) and an AAV-2/9 vector carrying MYO1C knockdown shRNA under the control of the podocyte makrer NPHS2 promoter were designed and synthesized, both tagged with EGFP. These vectors were custom synthesized by Hanbio Biotechnology (Shanghai, China), with detailed information provided in the Supplementary Method Materials. Ten-week-old male *db/db* mice were randomly divided into three groups: an untreated control group (*db/db*, n=10), an empty vector control group (AAV-Ctrl, n=10), and a MYO1C knockdown group (AAV-MYO1C, n=10). Mice were maintained under consistent feeding conditions. The *db/db* control group received no intervention, while the AAV-Ctrl group received a single tail vein injection of 120 μL of empty vector AAV (1.5×10^{6} vg/mL). The AAV-MYO1C group was administered a single tail vein injection of 100 μL AAV-MYO1C (1.8×10^{6} vg/mL).

Mouse urine samples were collected every two weeks and centrifuged at 10,000 rpm for 10 minutes at 4°C to collect the supernatant. Urine protein and creatinine levels were measured using an automated analyzer in the Nephrology Laboratory of the First Affiliated Hospital of Zhengzhou University. The urine albumin-to-creatinine ratio (UACR) was calculated. After 6–9 hours of fasting, mice were weighed on a milligram-precision balance. Tail blood was collected using a sterile lancet, and glucose levels were measured with a glucometer using fresh blood absorbed by glucose test strips. Measurements were repeated biweekly. After anesthesia, mice were secured, and thoracic exposure was performed. Blood was drawn from the right ventricle using a 1 mL syringe, followed by right atrial incision. PBS was perfused through the left ventricle until the kidneys turned pale. The kidneys were rapidly dissected, weighed, and processed as follows: 2–3 mm thick kidney cortex sections were fixed in glutaraldehyde for electron microscopy. Remaining kidney tissue portions were embedded in OCT compound, placed in light microscopy fixative, or stored in sterile, enzyme-free cryogenic tubes. These were immediately flash-frozen in liquid nitrogen and stored at -80°C. Blood samples were allowed to clot at room temperature and centrifuged to obtain serum. Serum biochemical parameters were analyzed using an automated biochemical analyzer.

Cell and kidney tissue samples RNA extraction and qRT-PCR. For mRNA reverse transcription, the RevertAid First Strand cDNA Synthesis Kit (K1622, ThermoFisher) was used according to the kit's instructions to synthesize cDNA. qRT-PCR was conducted using PowerUp™ SYBR™ Green Master Mix (A25742, ThermoFisher) to measure target gene expression levels. Primers used for qPCR analysis were custom-designed and synthesized by Sangon Bio (Shanghai, CHINA), with details provided in the Supplementary Method Materials. Experiments were performed in triplicate, using GAPDH mRNA amplification signals as the internal reference for normalization.

Histological analysis. For PAS staining, paraffin sections were deparaffinized, rehydrated through graded ethanol, and treated with periodic acid followed by Schiff reagent for visualization. After hematoxylin staining and differentiation with acid ethanol, sections were blued with running water, dehydrated, and mounted with neutral resin. Masson staining involved similar deparaffinization and rehydration, followed by Weigert's hematoxylin staining, acid ethanol differentiation, and rinsing. Sections were then treated with Masson's blue solution, stained with Ponceau red, counterstained with aniline blue, dehydrated, cleared in xylene, and mounted with neutral resin for microscopic evaluation. Kidney cortex specimens were fixed with glutaraldehyde,

post-fixed with osmium tetroxide, dehydrated in graded ethanol and acetone, embedded in epoxy resin, and imaged using TEM ((JEOL JEM-1400 Plus, USA) at 80 kV to assess ultrastructural features. Two independent observers blinded to the experimental groups evaluated all sections, with pathological features confirmed by pathologists.

Immunohistochemistry staining. Paraffin-embedded sections were deparaffinized and rehydrated through graded ethanol to distilled water. Antigen retrieval was performed in 1× sodium citrate buffer heated to 94°C, followed by cooling to room temperature. Endogenous peroxidase activity was blocked using a specific reagent for 5 minutes. Sections were incubated overnight at 4°C with a primary antibody against MYO1C (Abcam), followed by a 30-minute incubation at room temperature with an enzyme-labeled polymer secondary antibody (SD3101, Sinotech Bio). DAB substrate was applied for chromogenic detection, and sections were counterstained with hematoxylin, blued, dehydrated through graded ethanol, cleared in xylene, and mounted with neutral resin for microscopic observation.

Immunofluorescence Analysis. Kidney tissue specimens embedded in OCT or fixed in formalin and paraffin, as well as cultured HPCs, were subjected to immunofluorescence staining. Samples were fixed in cold methanol for 30 minutes, followed by PBS washes. Blocking was performed with 3% BSA solution for 1 hour. Specimens were incubated overnight at 4°C with primary antibodies against MYO1C (Abcam) and SYNPO (Proteintech). Subsequently, samples were incubated at 37°C for 1 hour with secondary antibodies conjugated to AlexaFluor 488 or 594 (Invitrogen, USA). F-actin staining was performed using Rhodamine Phalloidin (PHDR1, Cytoskeleton), and nuclei were counterstained with DAPI (Vector Laboratories, USA). Stained samples were visualized using a Zeiss LSM 880 confocal microscope (Carl Zeiss, Germany).

Protein Interaction Prediction. Protein-protein docking experiments were conducted to predict potential MYO1C and MAPK13 interactions. The AlphaFold-predicted structure of human MYO1C (UniProt ID: O00159) was retrieved, while MAPK13 (PDB ID: 4MYG) was used as the ligand. Docking was performed with optimized parameters, evaluating binding affinity via docking scores (lower scores indicate stronger binding) and confidence scores (high reliability if >0.7). LigPlot and PyMOL were employed for 2D/3D interaction visualization, detailing interface residues and interaction types. Using the Protein A/G Magnetic Co-IP kit (k1309, APExBIO), MYO1C and MAPK13 interactions were validated.
