# Supplementary material for: MYO1C is a urinary extracellular vesicle biomarker and mediator of podocyte injury in diabetic nephropathy
Source: JCI Insight. 2026 Jan 22;11(5):e194604. doi: 10.1172/jci.insight.194604 (PMC13041675; doi:10.1172/jci.insight.194604)

Figure 4B

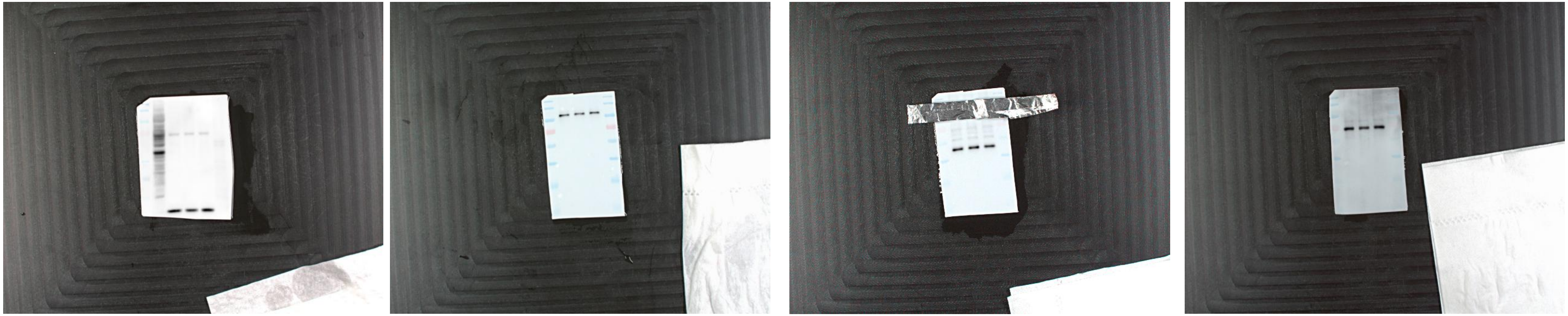

Figure 4D

MYO1C

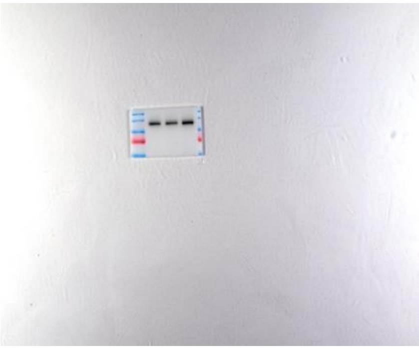

NPHS2

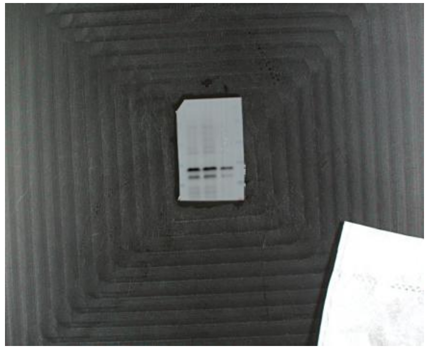

SYPNO

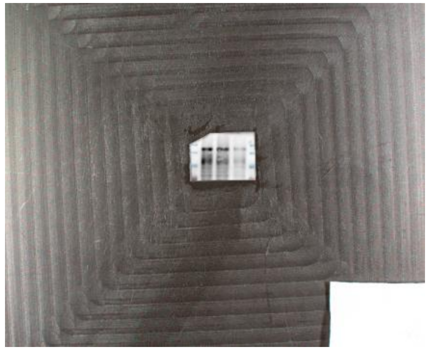

WT-1

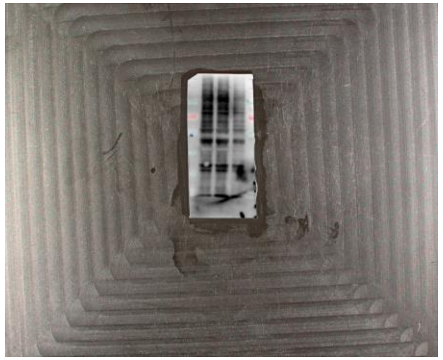

TNF-α

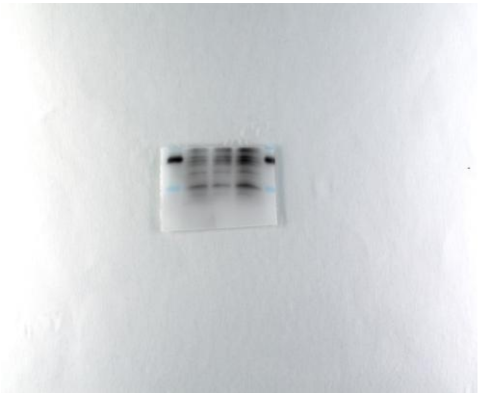

Cleaved caspased-3

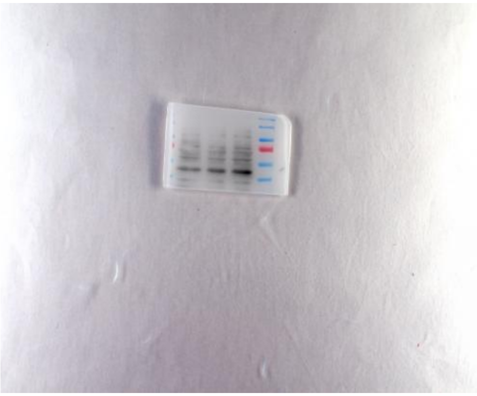

GAPDH

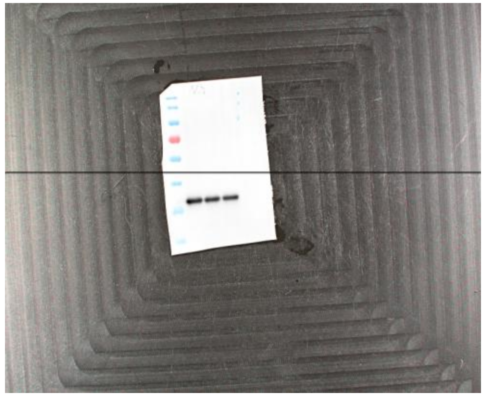

Figure 5H

MYO1C

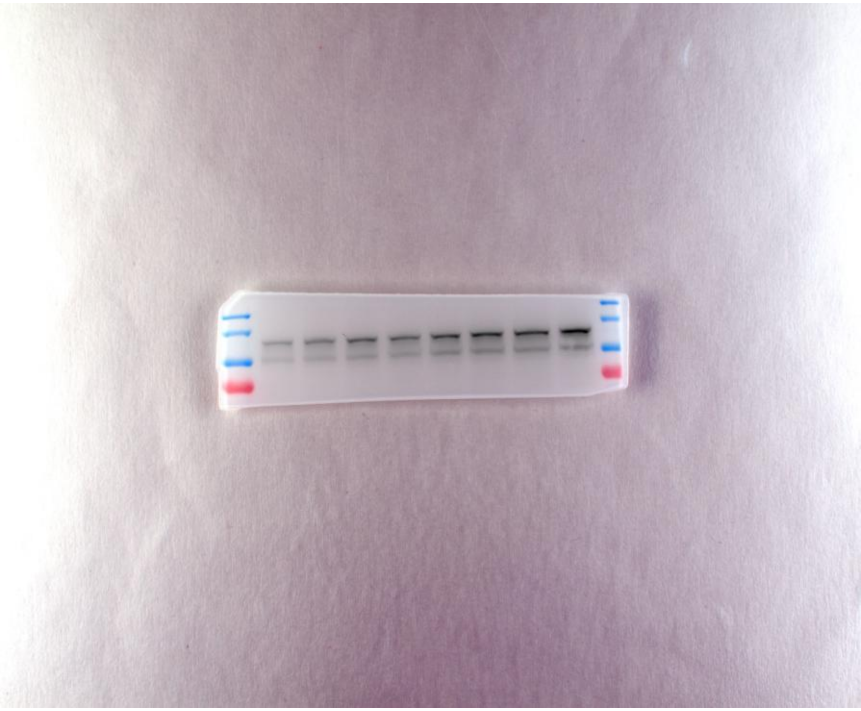

GAPDH

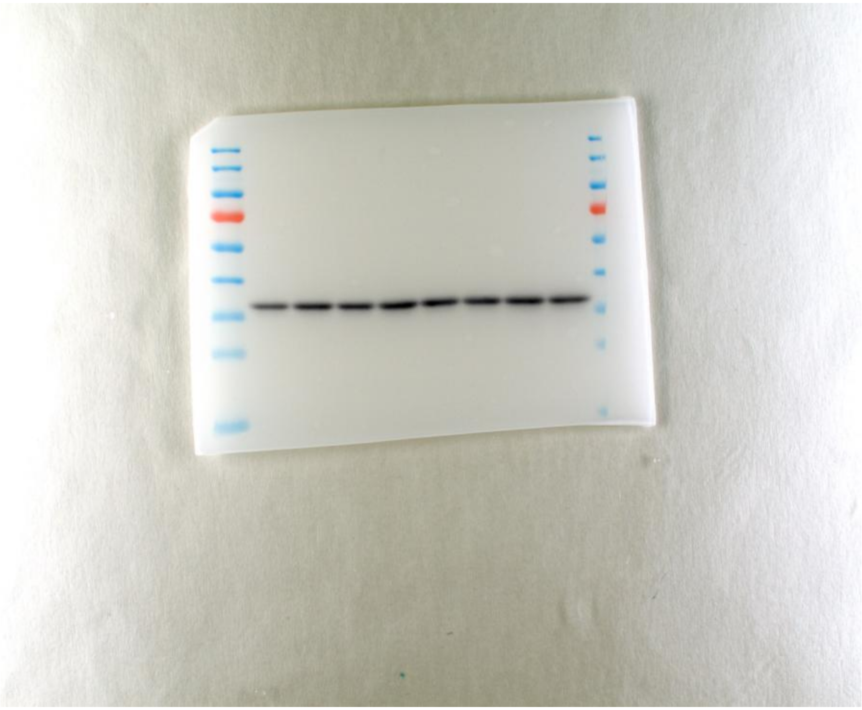

Figure 5I

MYO1C

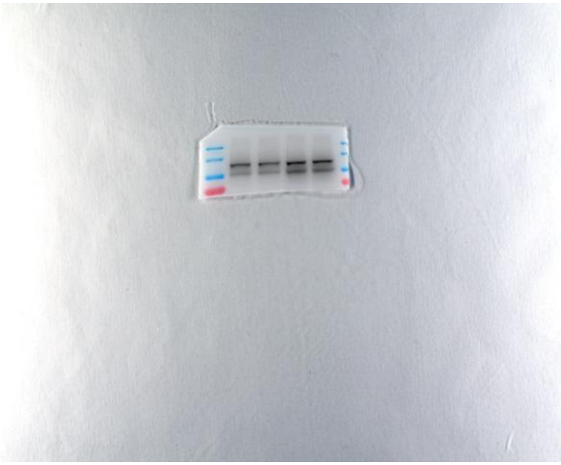

WT-1

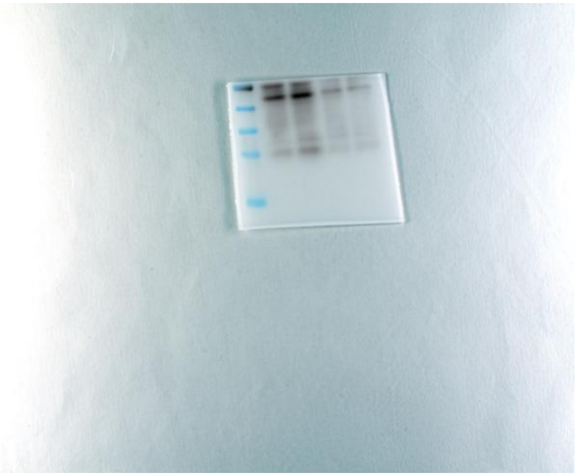

NPHS2

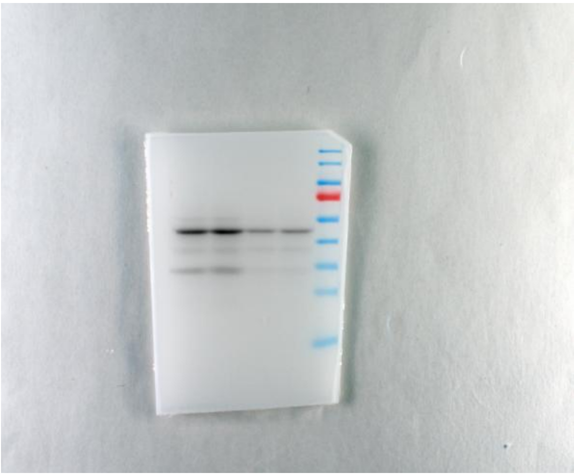

Desmin

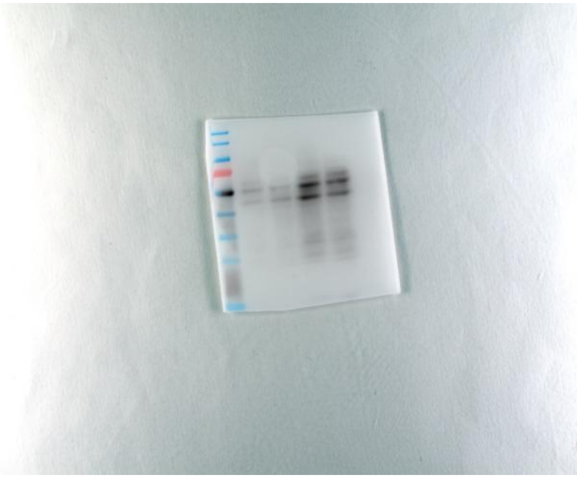

p38

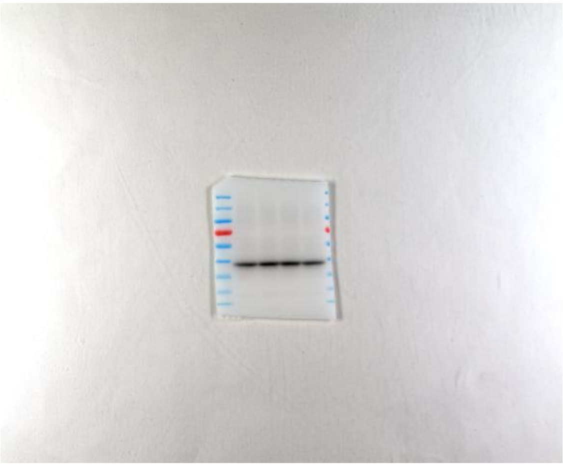

p-p38

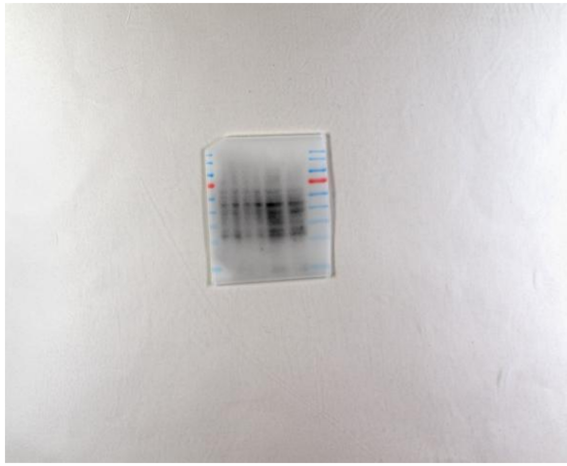

GAPDH

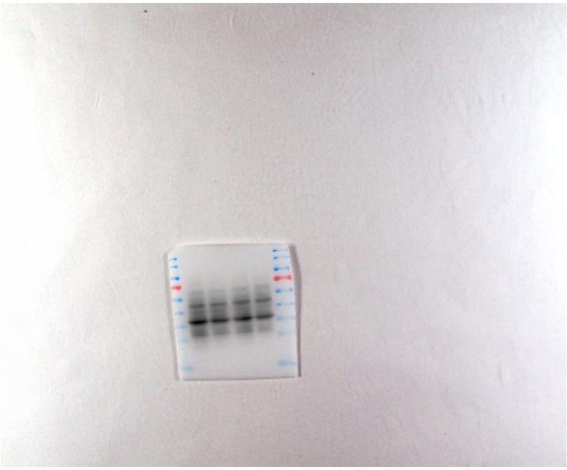

Figure 7A

MYO1C

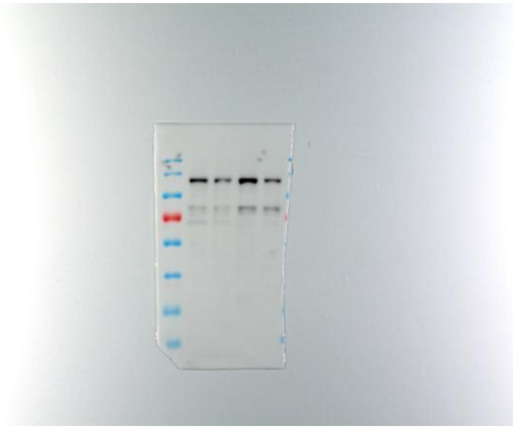

NPHS2

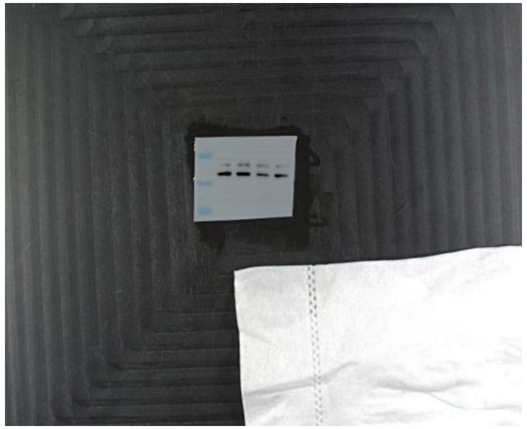

SYPNO

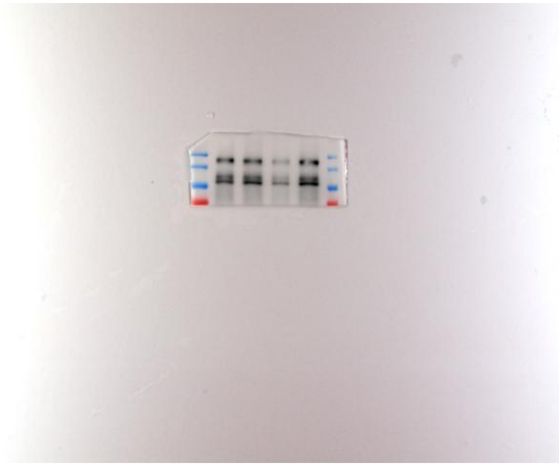

WT-1

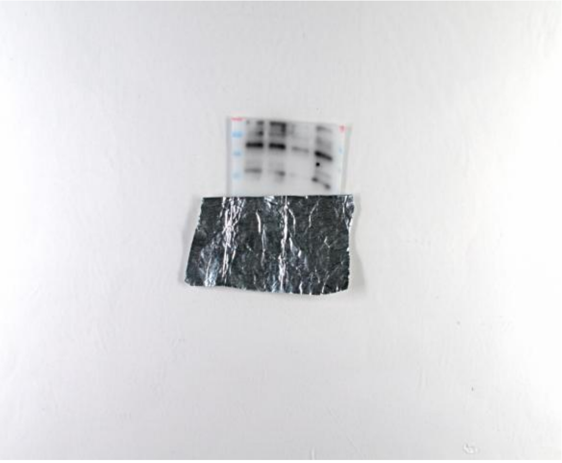

TNF-α

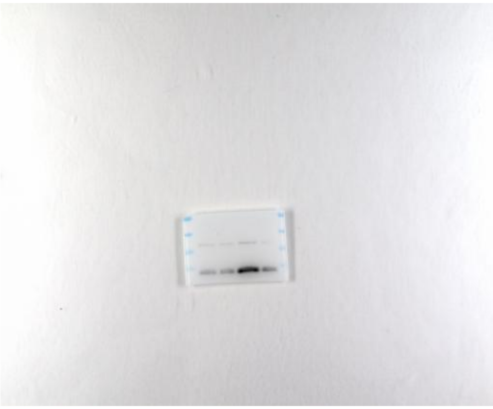

Cleaved caspased-3

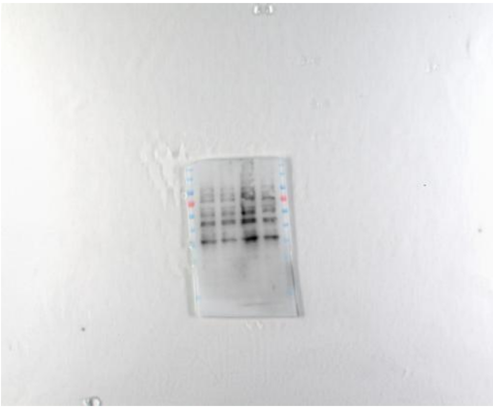

GAPDH

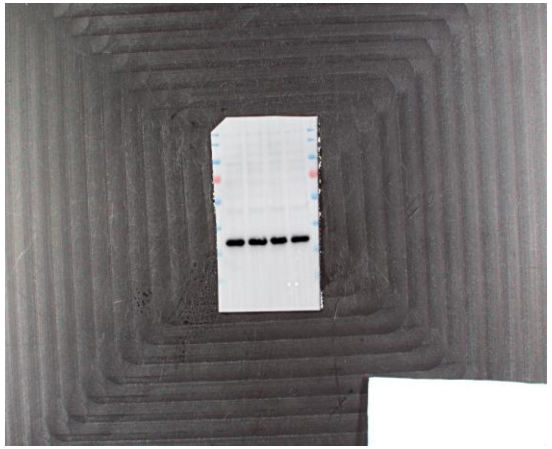

Figure 8G

MYO1C

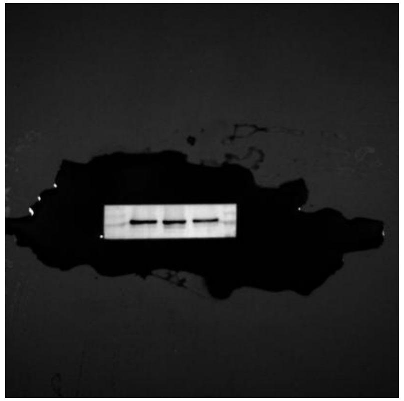

NPHS2

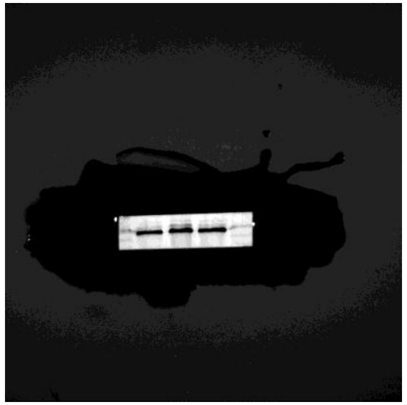

p38

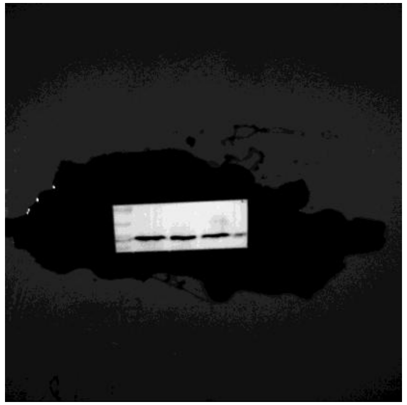

p-p38

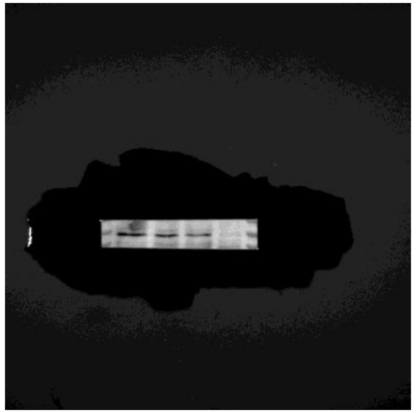

pCREB

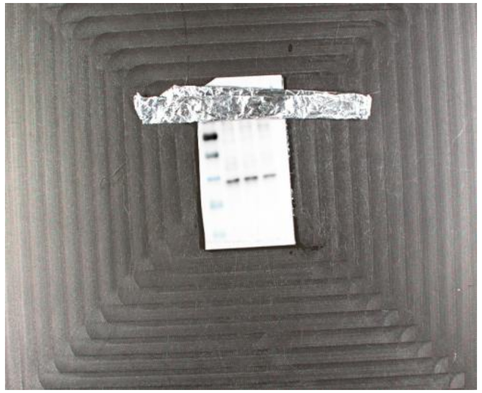

Cleaved caspased-3

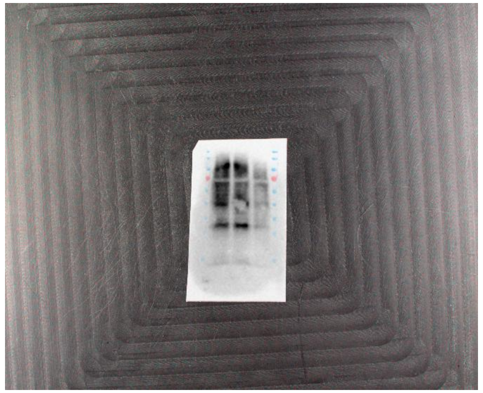

GAPDH

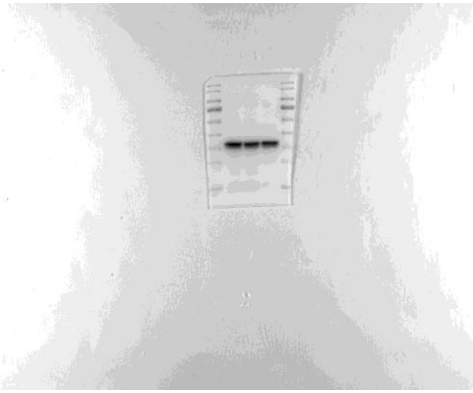

Figure 9C

MYO1C

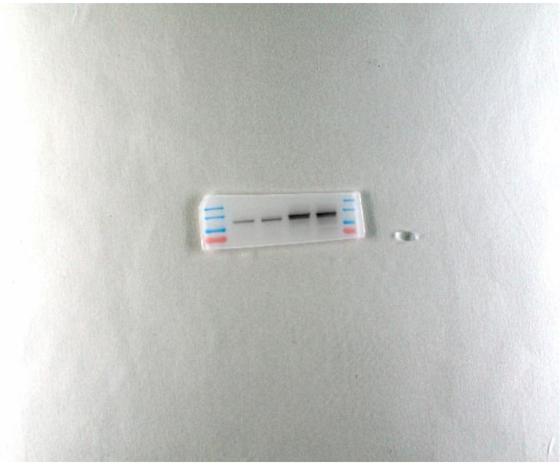

SYPNO

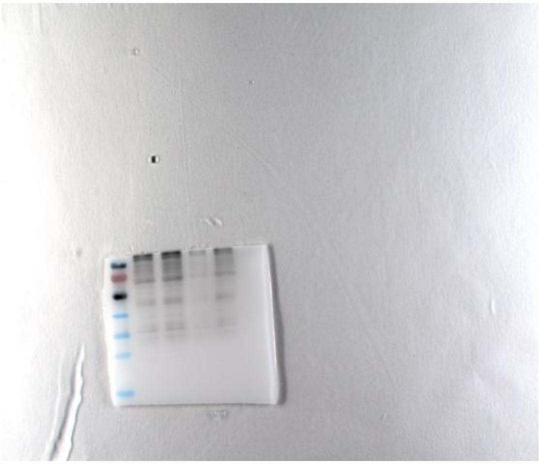

p38

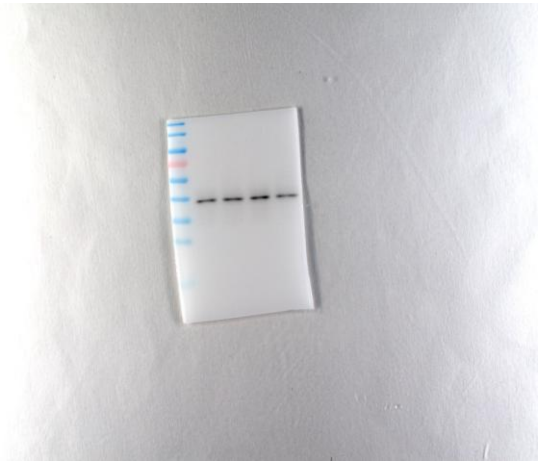

p-p38

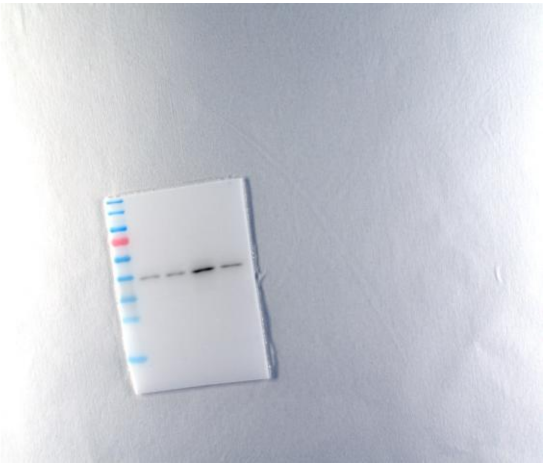

pCREB

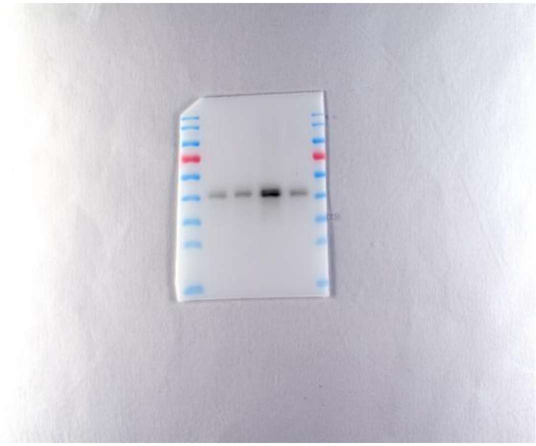

TNF-α

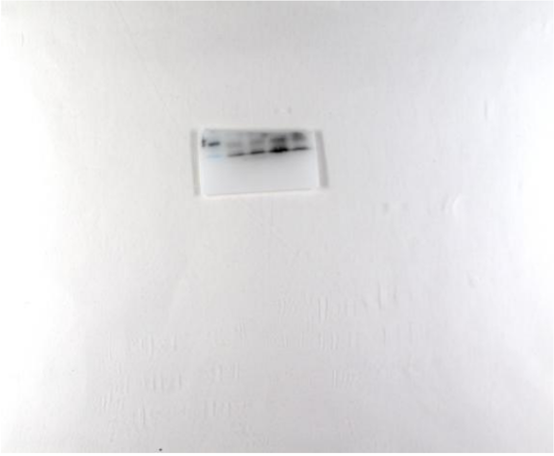

GAPDH

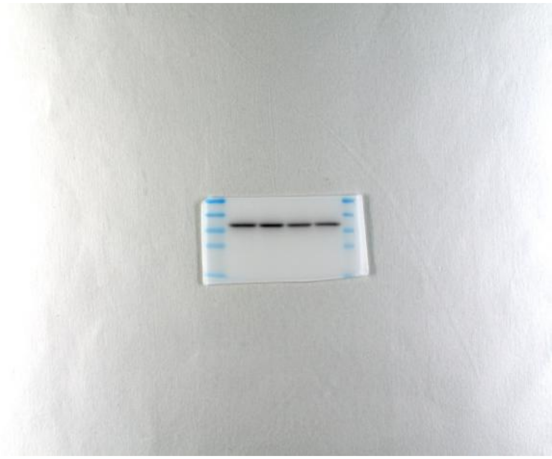

Figure 9E

MYO1C

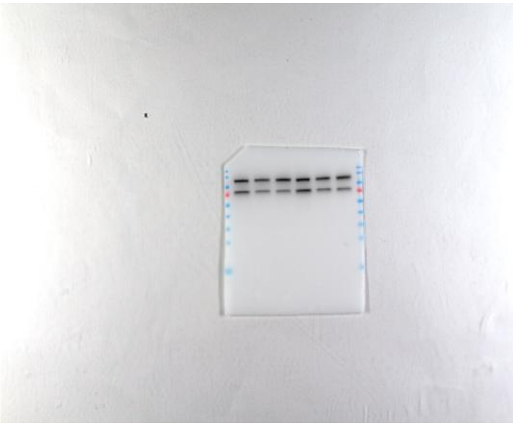

SYNPO

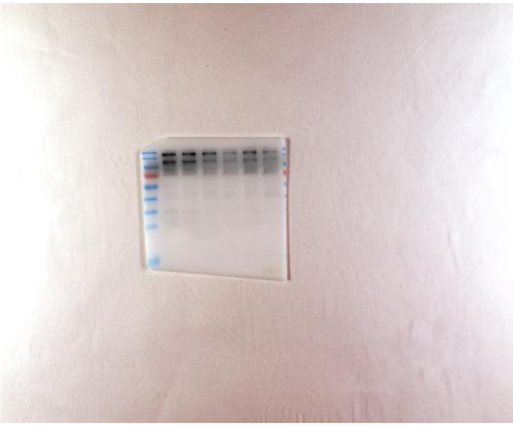

P38

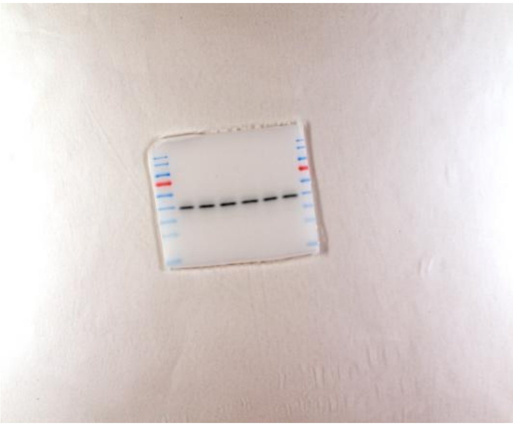

P-P38

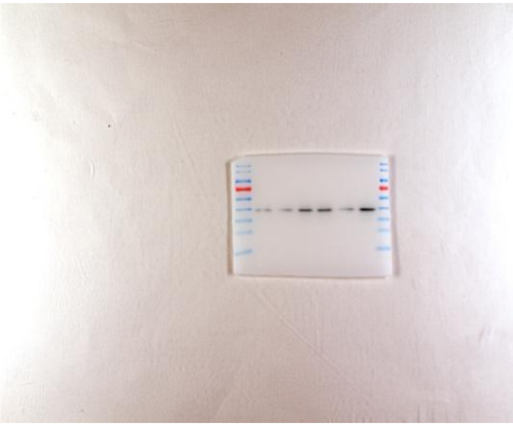

pCREB

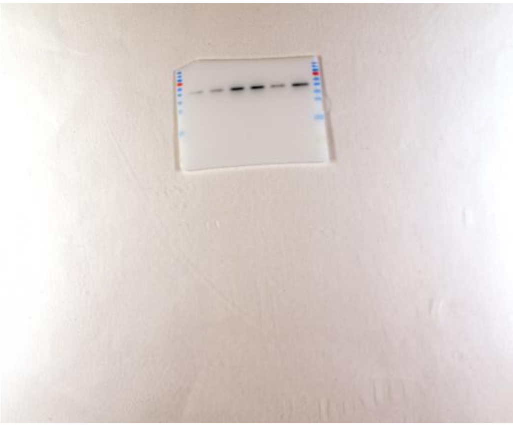

Cleaved caspased-3

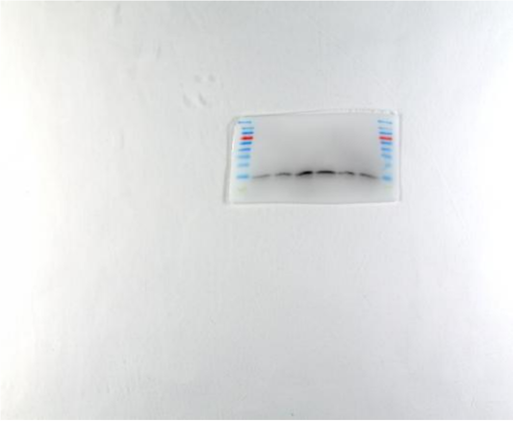

GAPDH

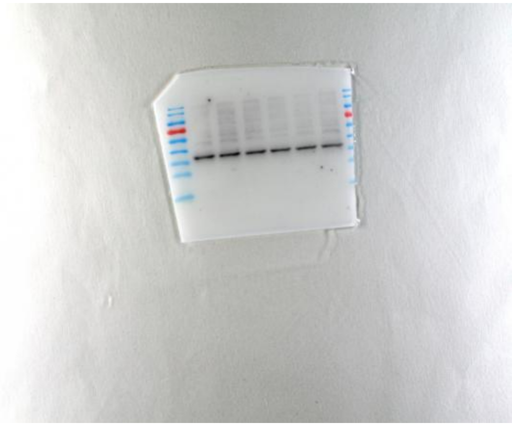

Supplement: Unedited blot and gel images [file jciinsight-11-194604-s266.pdf]
